# Economic Impact of Periodontitis: Global, Regional and National Estimates of Periodontal Expenditure, With Forecasts to 2050

**DOI:** 10.1111/jre.70104

**Published:** 2026-05-09

**Authors:** Dian Yi Chow, Robin D. Blythe, John Rong Hao Tay, Gustavo G. Nascimento, Mario Romandini

**Affiliations:** ^1^ Department of Restorative Dentistry National Dental Centre Singapore Singapore Singapore; ^2^ Programme in Health Services Research & Population Health Duke‐NUS Medical School Singapore Singapore; ^3^ National Dental Research Institute Singapore National Dental Centre Singapore Singapore Singapore; ^4^ School of Dentistry University of Utah Salt Lake City Utah USA; ^5^ Ninth People's Hospital, Shanghai Jiao Tong University School of Medicine Shanghai China

**Keywords:** dental expenditure, forecasting, global burden of disease 2021 (GBD 2021), global health, health expenditures, healthcare utilisation, Monte Carlo simulation, periodontal diseases, universal health coverage, World Health Organisation (WHO)

## Abstract

**Aim:**

To estimate the global, regional and national economic impact of periodontitis in 2021 (including expenditure on prevention, diagnosis, treatment, maintenance and rehabilitation), and to forecast expenditures through 2050 under two utilisation scenarios: continuation of current treatment coverage trends and an illustrative expanded coverage scenario.

**Methods:**

A cohort‐based gross‐costing Monte Carlo model was developed to estimate global, regional and national expenditure on periodontitis in 2021. Model estimates were validated against known national periodontal spending and aggregate dental expenditure. The model was then applied to generate forecasts for 2025–2050 at five‐year intervals under two utilisation scenarios: (i) continuation of current utilisation patterns of periodontal care and (ii) an illustrative expanded coverage scenario in which 80% of the population receives periodontal care by 2030.

**Results:**

In 2021, global expenditure on periodontitis was US$168.1 billion (95% UI: 133.8–202.4), with marked regional disparities; five countries (the United States, China, Germany, Japan and France) accounted for more than two‐thirds of global spending. Under current utilisation trends, global expenditure is projected to increase modestly to US$174.9 billion by 2050 (95% UI: 137.4–212.3), despite a projected 44.3% increase in severe periodontitis prevalence. Under the expanded coverage scenario, expenditure would instead increase threefold to US$500.6 billion (95% UI: 389.2–612.1) by 2050, implying an annual financing gap of US$325.7 billion. Global per‐capita expenditure was US$21.32 (95% UI: 16.97–25.67) in 2021, projected to decline to US$18.36 (95% UI: 14.43–22.29) in 2050 under current utilisation, but to rise to US$52.56 (95% UI: 40.86–64.26) under the expanded coverage scenario.

**Conclusion:**

Periodontitis imposes a substantial and unequally distributed economic impact worldwide. Under current utilisation patterns, the projected rise in prevalence by 2050 will primarily translate into unmet care needs. Achieving universal coverage under WHO targets would require tripling global periodontal expenditure—a level unlikely to be feasible—highlighting the need to rethink current models of periodontal care delivery.

## Clinical Relevance

**Background:**

Quantifying the economic impact of periodontitis is essential for understanding its societal burden, guiding resource allocation, informing prevention and policy strategies, and assessing the value of disease prevention. Yet no comprehensive global, regional and national estimates of periodontal expenditure currently exist.

**Added Value of This Study:**

This study provides the first global, regional and national estimates of expenditure on periodontitis from 2021 to 2050. A new cohort‐based gross‐costing model incorporating the full spectrum of periodontal care—including prevention, diagnosis, active treatment, maintenance and rehabilitation—was developed and validated, allowing comprehensive cost estimation for all countries worldwide. In addition to projecting expenditure under continuation of current utilisation patterns, an illustrative expanded coverage scenario was modelled in which periodontal care reaches 80% population coverage by 2030.

**Clinical Implications:**

These findings are intended to inform population‐level planning and policy development, rather than clinical decision‐making for individual patients. With periodontitis prevalence projected to rise, maintaining current patterns of periodontal care utilisation will lead to rising unmet needs, whereas uncritical expansion of coverage under existing care models is unlikely to be financially feasible. A reorientation of periodontal care delivery beyond predominantly professional‐led approaches is therefore necessary. Strategies centred on common risk factor prevention in the context of multimorbidity, more targeted diagnosis, and novel pharmacological therapies may offer more scalable and cost‐effective ways to reduce the future burden of periodontitis.

## Graphical abstract

This study provides the first global, regional and national estimates of periodontal expenditure, with projections to 2050. Costs related to prevention, diagnosis, treatment, maintenance and rehabilitation are quantified across all countries worldwide. Despite a rising disease burden, most patients with periodontitis are projected to remain untreated under current care models, whereas universal coverage would require a threefold increase in global spending.

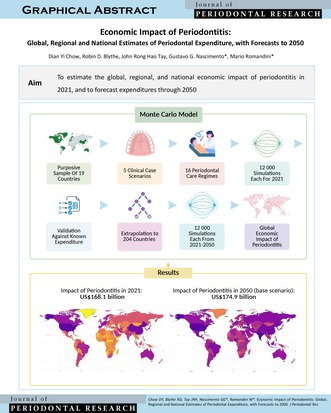

## 1 Introduction

Periodontitis is an inflammatory disease of the tooth‐supporting tissues that results from complex and still incompletely deciphered interactions between the oral microbiome and the host immune response [1, 2, 3, 4, 5, 6]. In its severe forms, progressive destruction of periodontal structures leads to tooth loss, impaired mastication with potential nutritional consequences [7], reduced self‐esteem related to aesthetic sequelae [8], and poorer oral health‐related quality of life [9]. Beyond the oral cavity, periodontitis contributes to and exacerbates multi‐morbidity [10, 11, 12, 13, 14], carries important social implications [15], and generates substantial costs related to prevention, diagnosis, treatment, maintenance and rehabilitation [16, 17, 18, 19]. In 2021, severe periodontitis affected more than one billion people worldwide, with projections indicating a rise to 1.5 billion by 2050 [20]. These figures underscore its status as a major, yet largely unresolved, global public health challenge.

In 2024, the World Health Organisation (WHO) launched the Global Oral Health Action Plan (GOHAP) [21], setting an ambitious target to extend public financial coverage for essential oral health services to 80% of the global population by 2030, compared with 23% in 2020 [22]. The Action Plan does not specify disease‐specific coverage targets and deliberately leaves the definition of essential oral health services to individual countries. Notably, the WHO's illustrative service package excludes, however, periodontal care, prioritising instead dental caries and oral cancer [21]. This position contrasts with recommendations from other stakeholders. In particular, the European Federation of Periodontology (EFP) has recently called for a substantial expansion of preventive, diagnostic and therapeutic care for periodontitis [23].

Such prioritisation decisions require robust estimates of both current and future costs. Although cost estimates alone cannot determine allocative efficiency or rank interventions across competing health priorities, they are a necessary input for assessing the fiscal implications of current and alternative coverage scenarios [24], and for evaluating whether currently prevailing models of care delivery are compatible with population‐level coverage. Moreover, quantifying the economic impact of periodontitis is essential to capture its full societal impact, and to inform policy and prevention strategies.

Despite this need, comprehensive estimates of the global economic impact of periodontitis management remain limited. Previous studies have either reported aggregated oral health expenditures [25] or focused on specific countries and regions [18]. Existing analyses have also typically been limited to non‐surgical periodontal treatment, without accounting for the broader cost spectrum that includes prevention, diagnosis, surgical treatment, maintenance and rehabilitation of periodontitis‐related tooth loss. In addition, available studies have relied on top‐down proportional methods, which may obscure country‐specific demographic characteristics and patterns of service utilisation. Finally, projections of future periodontitis‐related expenditures and associated financing gaps are currently unavailable, limiting policymakers' ability to assess the long‐term financial implications of current and alternative coverage scenarios.

This study addresses these gaps by:

- Estimating the global, regional and national economic impact of periodontitis in 2021 using a bottom‐up, procedure‐based costing framework encompassing prevention, diagnosis, active treatment, maintenance and rehabilitation; and
- Forecasting expenditures through 2050 under two utilisation scenarios: continuation of current treatment coverage trends and an illustrative expanded coverage scenario achieving 80% population coverage for periodontal care by 2030.

## 2 Methods

This manuscript adheres to the Consolidated Health Economic Evaluation Reporting Standards 2022 (CHEERS 2022) [26]. Model development followed established best practices for health economic microsimulation modelling, including internal consistency checks, assessment of parameter plausibility and scenario‐based validation against external data sources where available.

**FIGURE 1:**
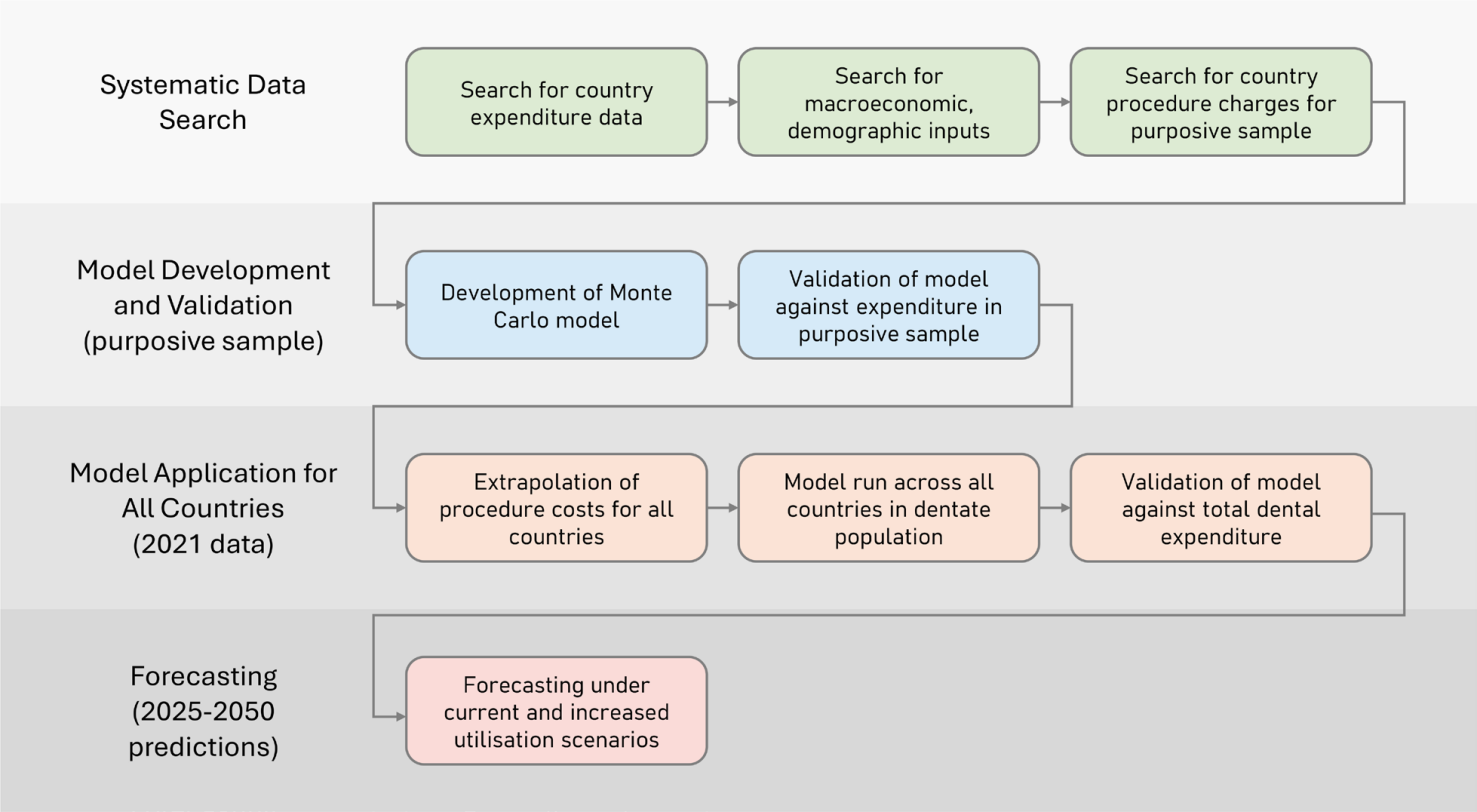
Study workflow.

Figure 1 presents an overview of the study workflow. A cohort‐based gross costing approach was adopted [27], whereby each national periodontal expenditure was estimated as the sum of individual procedural costs applied across its population. Model inputs were derived from a systematic search aimed at identifying national periodontal expenditure data for all 204 countries included in the Global Burden of Disease (GBD) study, where available, together with detailed procedure‐level charges from a purposive sample of 19 countries. The purposive sample, informed by the search results, prioritised countries with available national periodontal expenditure estimates, while capturing a substantial share of global dental expenditure and ensuring representation across income levels. Data from this sample informed the development and validation of a Monte Carlo model. Procedure costs derived from the purposive sample were then extrapolated to the remaining 185 countries, with adjustments based on Gross Domestic Product (GDP) per capita. Where country‐level periodontal expenditure data were available, these were used for model validation and calibration during model development but were not directly incorporated as model inputs. Using these country‐specific cost inputs, the model was then applied to estimate periodontal expenditure in 2021 (based on observed data) and to generate forecasts for 2025–2050 at five‐year intervals. Estimates for 2025 remain model‐based projections, as comprehensive country‐level data were not yet available at the time of manuscript preparation.

The analysis followed a prospectively agreed plan within a structured modelling framework. Details of model development, assumptions, diagnostics and validation procedures are reported below and in the Supporting Information. All analyses were conducted using R version 4.5.1 [28]; full analytical codes are provided in Methods S1 and in a public repository [29]. All costs are reported in 2025 US dollars.

### 2.1 Data Sources

#### 2.1.1 Periodontitis and Aggregate Dental Expenditure Data

Country‐level periodontitis expenditure data were systematically sought for all 204 GBD countries through a search of PubMed and Embase via OVID. As many relevant data sources are contained in official government documents not indexed in scholarly databases, this search was supplemented with structured searches on Google, the WHO Global Health Expenditure Database [30], the OECD Data Explorer [31] and the FDI Oral Health Atlas [32], conducted between 25 December 2024 and 15 January 2025. Search strings were tailored to each country based on the national language, the official name of the Ministry of Health and the relevant public health insurance scheme. The full search strategy is detailed in Methods S2, and its results are reported in Methods S3. In addition, government health databases were hand‐searched to identify additional records, including data available only upon request to country‐level researchers and health officials. Aggregate dental expenditure data (i.e., not limited to periodontitis) were additionally sourced from Jevdjevic & Listl [25].

#### 2.1.2 Charges for Individual Periodontal Procedures

Charges for 16 periodontal interventions related to prevention, diagnosis, treatment, maintenance and rehabilitation of periodontitis‐related tooth loss were compiled from a purposive sample of 19 countries. This sample was selected based on the systematic search results to capture major contributors to global dental expenditure, while also reflecting heterogeneity in economic context and dental care utilisation patterns. It included 2 low‐income (Rwanda and Tanzania), 4 middle‐income (Brazil, Botswana, Chile and China) and 13 high‐income countries (Australia, Canada, France, Germany, Italy, Japan, the Republic of Korea, Spain, Sweden, Switzerland, Taiwan, the United Kingdom and the United States) according to the GBD classification and collectively accounted for approximately 89% of global dental expenditure.

Countries in the purposive sample also reflected diversity in periodontal care delivery and financing models relevant to costing. These included settings with near‐universal public coverage (e.g., Sweden, Brazil and Taiwan) [33, 34], strong social insurance systems with negotiated fee schedules (e.g., Japan, Germany and Korea) [35] and predominantly private or out‐of‐pocket financing (e.g., Australia, Switzerland and the United States) [36]. This diversity captures important differences in financing structures and utilisation incentives shaping periodontal care delivery. Specialist availability also varied substantially, ranging from minimal documentation of periodontists in Botswana, Rwanda and Tanzania to the United States, where 73% of adults live within 10 miles of a periodontist [37].

Tariff schedules were retrieved from public health insurers, private insurers and public tertiary care centres. This triangulated approach was used to derive representative national‐level estimates of procedure charges. Detailed country‐specific sources are reported in Methods S4. Searches for procedure charges were not restricted to English‐language sources. Tariff schedules were frequently poorly indexed and accessible only through official national websites in local languages, necessitating language proficiency during the search and data extraction processes. Documents in English, Chinese, Spanish, Danish, Norwegian, German, Portuguese and Italian were reviewed directly by the authors. For other languages represented in the sample (French, Japanese and Korean), tariff information was translated by fluent collaborators and cross‐checked against automated translation tools.

#### 2.1.3 Demographic and Epidemiological Data

Country‐level estimates of population size, GDP per capita and the prevalence of severe periodontitis and edentulism in 2021 were sourced from the Global Health Data Exchange [38, 39, 40]. Forecasts of population size and prevalence of severe periodontitis and edentulism to 2050 were obtained from the GBD 2021 Forecasting Collaborators, as part of a prior study [20].

### 2.2 Phase 1: Monte Carlo Simulation Model Development Based on 19 Countries

A Monte Carlo model was first developed using a purposive sample of 19 countries that contributed data on 16 periodontal procedures and their associated fees. A formal health‐care sector perspective was adopted, consistent with the study aim of quantifying the financial gap between current coverage patterns and expanded coverage scenarios for periodontal care. A one‐year time horizon was applied, reflecting the typical duration of comprehensive periodontal treatment [41].

#### 2.2.1 Model Structure: Clinical Case Scenarios, Periodontal Care Statuses and Periodontal Care Regimens

A cohort‐based gross‐costing model was constructed. Within each of the 19 countries, the population was divided into multiple cohorts, each representing a distinct clinical case‐scenario. For each cohort, treatment costs were estimated by summing the costs of all procedures included in the corresponding standardised care regimen. National periodontal expenditure was then derived by aggregating costs across all cohorts.

Model estimates stabilised rapidly across all super‐regions except South Asia. Based on the rate of decline of the Monte Carlo standard error (MCSE) for South Asia, 12 000 iterations were selected [42]. Evidence of model convergence is presented in Methods S5.

After exclusion of the edentulous population, each country's dentate population was classified into five mutually exclusive clinical case scenarios, based on prevailing case definitions for periodontitis [8]. Stage IV periodontitis was further stratified into case types reflecting progressively greater rehabilitative needs [41]. These clinical case scenarios corresponded to standardised treatment regimens and comprised:

1. Health/gingivitis;
2. Stage I‐II periodontitis;
3. Stage III and stage IV periodontitis, case types 1–2;
4. Stage IV periodontitis, case type 3;
5. Stage IV periodontitis, case type 4.

Because population‐level epidemiological data using these detailed case definitions are not available, prevalence estimates from GBD 2021 were used as the starting point to derive case‐scenario distributions. GBD 2021 reports prevalence estimates only for severe periodontitis [20]; prior studies have shown this category to correspond broadly to stage III and stage IV periodontitis in an approximate 60:40 ratio [43, 44]. Stage IV case types 1–4 were assumed to be equally distributed, such that each represents 10% of severe cases. On this basis, individuals with severe periodontitis were probabilistically allocated across the three advanced clinical case scenarios using a Dirichlet distribution with relative weights of 8:1:1, corresponding to central estimates of 80% for stage III and stage IV case types 1–2, and 10% each for stage IV case types 3 and 4. Wide uncertainty intervals were specified (95% CI: 52%–97%, 0.3%–34%, and 0.3%–34%) to reflect limited evidence on the true population distribution. The remaining dentate population without severe periodontitis was allocated equally between health/gingivitis and stage I–II periodontitis, consistent with prospective [44] and retrospective [43, 45] applications of the ACES framework [43]. Uncertainty was incorporated by sampling from a Dirichlet distribution with relative weights 5:5, corresponding to central estimates of 50% each and wide uncertainty intervals (95% CI: 21%–78%) reflecting limited evidence on the true distribution.

Each clinical case‐scenario was then stratified into three periodontal care statuses:

1. No periodontal care (no diagnostic, preventive, or therapeutic procedures received during the year),
2. Active periodontal care (comprehensive diagnostic, preventive and therapeutic procedures as indicated, including rehabilitation of periodontitis‐related tooth loss),
3. Maintenance (supportive periodontal care).

Proportions of individuals in each care status were derived from epidemiological studies [46, 47] with uncertainty incorporated using a Dirichlet distribution.

For each clinical case‐scenario, a standardised periodontal care regimen was defined. Regimens comprised procedures delivered during the preventive, diagnostic and extraction phase (e.g., consultations, radiographs, prophylaxis, extractions), active periodontal treatment (e.g., oral hygiene instruction, supra‐ and sub‐gingival instrumentation, periodontal surgery), maintenance (supportive periodontal care) and rehabilitative care (e.g., implant placement, implant‐supported restorations, fabrication and repair of fixed or removable prostheses). Regimens were structured to align with how procedures are typically grouped within dental coding systems, facilitating validation against country‐level aggregated expenditure reports.

For each country, the number of procedures performed per clinical case‐scenario was sampled from normal distributions informed by clinical practice guidelines [41, 48], population‐based cohort studies [49, 50] and insurance claims databases [51]. Procedure‐specific unit costs were sampled from Gamma distributions derived from national dental fee schedules.

#### 2.2.2 Dental Utilisation Scenarios

To reflect substantial cross‐country variation in access to and utilisation of periodontal care, three dental utilisation scenarios were defined and applied to both the purposive sample (as described in this section) and the remaining countries (as described in Section 2.3): low, medium and high. The decision to model three discrete categories was based on the trimodal, right‐skewed distribution of national direct dental expenditures reported previously in Jevdjevic & Listl [25]. Scenarios differed with respect to both periodontal care status distributions (no care, active care, maintenance) and the composition of periodontal care regimens (number and type of procedures). Countries in the low‐utilisation scenario were modelled as relying more heavily on non‐surgical care and extractions, whereas those in the high‐utilisation scenario incorporated greater use of rehabilitative procedures.

The high‐utilisation scenario corresponded to the default periodontal care statuses and regimens described in Section 2.2.1.

The medium‐utilisation scenario retained the same care regimens but adjusted care status distributions to reflect real‐world utilisation patterns in countries with intermediate access to dental care, informed by epidemiological surveys from Brazil [52], Malaysia [53], Iran [54, 55] and Thailand [56].

The low‐utilisation scenario represented predominantly acute, problem‐driven dental attendance. Care regimens were modified to prioritise extractions [57], reflecting barriers to comprehensive care including cost [58], limited equipment availability [59], and insufficient specialist expertise and laboratory support [60, 61]. Periodontal care status distributions for this scenario were informed by epidemiological data from Burkina Faso [62], Sudan [63], and a scoping review encompassing 27 low‐income countries [64].

Parameters and assumptions underpinning each utilisation scenario are detailed in Methods S6.

#### 2.2.3 Estimation Process

For each iteration, random draws were made from all parameter distributions. For each country in the purposive sample, sub‐national cohort sizes were generated based on sampled population counts, clinical case‐scenario distributions and periodontal care status distributions. Treatment costs for each cohort were calculated using sampled numbers of procedures and their associated unit costs. Annual country‐level periodontal expenditure was calculated by summing the costs across all cohorts.

Across iterations, median values and standard errors of the mean were summarised. This process was conducted separately for countries assigned to each of the three dental utilisation scenarios.

To prevent implausible estimates, 75% of total dental expenditure was imposed as the upper bound for model‐predicted periodontal expenditure. This threshold was informed by evidence indicating that periodontitis accounts for approximately 48% of the global burden of oral diseases [65]. Considering frequent co‐occurrence with other oral conditions, an uncertainty margin of approximately ±50% around this value was deemed reasonable in the absence of published expenditure‐based proportions. This constraint should be interpreted as a pragmatic structural safeguard against implausible extremes, rather than a direct proxy for true expenditure. One‐way sensitivity analyses were performed to assess the impact of reducing the upper bounds to 50% and 60%.

Model validation within the purposive sample was performed by comparing model‐estimated periodontal expenditures against known country‐specific data (Methods S7). Additional one‐way sensitivity analyses evaluated the impact of alternative assumptions regarding clinical case‐scenario distributions and periodontal care regimens.

### 2.3 Phase 2: Extrapolation to the Remaining Countries

Following internal validation, the model was extrapolated to the remaining 185 countries included in the GBD study. During extrapolation, each country was assigned to one of the three dental utilisation scenarios (low, medium, high), as defined in Section 2.2.2.

To account for cross‐country variation in procedure costs, a cost‐adjustment factor based on GDP per capita was derived. Professional supra‐gingival instrumentation was selected as the reference procedure, as it demonstrated stable cost ratios relative to other procedures and consistent behaviour across countries.

Procedure costs were extrapolated in two steps. First, the cost of supra‐gingival instrumentation was modelled using a log–log relationship with nominal GDP per capita. Nominal GDP per capita was preferred over Purchasing Power Parity (PPP) adjustments to better reflect implications for national health budgets under expanded coverage. Second, costs for all other procedures were expressed as multiples of the cost of supra‐gingival instrumentation. This two‐step approach preserved the multiplicative relationship between GDP per capita and dental fees while avoiding excessive variance inflation for procedures with wide cost ranges, such as full‐arch fixed restorations. The resulting extrapolated cost distributions were then converted into Gamma distributions to ensure all sampled costs remained positive. Model diagnostics for both extrapolation steps are reported in Methods S8 and S9 and were used to assess gross model misspecification rather than inferential validity.

The full extrapolated model was validated against known national dental expenditure data [25]. Validation results are presented in Methods S10.

### 2.4 Phase 3: Forecasting to 2050 Under Two Utilisation Scenarios

The validated model was finally applied to all 204 countries to project periodontal expenditure from 2025 to 2050 at 5‐year intervals, using 12 000 iterations per time point. Forecasts incorporated anticipated demographic changes and projected trends in severe periodontitis prevalence [20]. All forecasted estimates are reported in 2025 US dollars at current prices, reflecting the assumption that medical inflation tracks general inflation; consequently, results are not sensitive to the choice of discount rate.

Two dental utilisation scenarios were modelled. The base, current‐usage scenario assumed that existing patterns of periodontal care utilisation remain unchanged over time. The expanded coverage scenario simulated a situation in which 80% of individuals receive periodontal care by 2030, using the WHO GOHAP ambition as a conceptual benchmark. This scenario does not represent a WHO‐endorsed service package but serves as an illustrative analytical stress test to quantify potential expenditure requirements and financing gaps.

For the expanded coverage scenario, a conservative assumption was adopted whereby increases in dental utilisation were reflected primarily in a higher proportion of individuals receiving maintenance (supportive periodontal care), while the proportion requiring the more resource‐intensive active treatment phase remained constant. Because robust evidence on the population‐level effectiveness of preventive interventions is lacking [66], the model does not incorporate potential reductions in disease incidence or recurrence associated with increasing utilisation.

## 3 Results

Global and super‐region estimates of periodontitis expenditure for 2021 and 2050 are summarised in Table 1 (total expenditure) and Table 2 (per‐capita expenditure). Region‐ and country‐level results are reported in Appendix 1 (total expenditure) and Appendix 2 (per‐capita expenditure).

**TABLE 1:** Global and super‐region total periodontal expenditure (2025 US$ billions) in 2021, with projections to 2050 under current‐usage and expanded coverage scenarios.

Super‐region	2021 total Expenditure (US$ billions [95% UI])	2021 total Preventive, Diagnostic and Extraction Phase Expenditure (US$ billions [95% UI])	2021 total Active Treatment Expenditure (US$ billions [95% UI])	2021 total Maintenance Expenditure (US$ billions [95% UI])	2021 total Rehabilitative Care Expenditure (US$ billions [95% UI])
**2021**					
Global	168.1 (133.8–202.4)	112.2 (83.0–141.5)	9.5 (4.1–15.0)	21.5 (13.1–29.9)	24.9 (10.1–39.7)
GBD super‐region					
Central Europe, Eastern Europe and Central Asia	2.0 (0.7–3.3)	1.0 (0.0–2.1)	0.2 (0.0–0.5)	0.2 (0.0–0.6)	0.5 (0.0–1.1)
High‐income	129.4 (98.2–160.6)	88.0 (61.3–114.7)	6.7 (2.0–11.3)	16.6 (9.0–24.2)	18.2 (4.8–31.6)
Latin America and Caribbean	3.2 (0.5–5.9)	2.1 (0.0–4.6)	0.3 (0.0–0.8)	0.3 (0.0–0.9)	0.5 (0.0–1.1)
North Africa and Middle East	3.0 (1.0–5.1)	1.7 (0.0–3.3)	0.3 (0.0–0.8)	0.3 (0.0–0.8)	0.7 (0.0–1.7)
South Asia	0.9 (0.0–5.0)	0.5 (0.0–3.9)	0.1 (0.0–1.2)	0.2 (0.0–2.2)	0.1 (0.0–0.8)
Southeast Asia, East Asia and Oceania	29.0 (15.8–42.1)	18.5 (7.5–29.5)	1.9 (0.0–4.5)	3.7 (0.8–6.6)	4.8 (0.0–10.8)
Sub‐Saharan Africa	0.7 (0.0–1.4)	0.5 (0.0–1.2)	0.0 (0.0–0.1)	0.1 (0.0–0.3)	0.1 (0.0–0.3)

Super‐region	2050 total Expenditure (US$ billions [95% UI])	2050 total Preventive, Diagnostic and Extraction Phase Expenditure (US$ billions [95% UI])	2050 total Active Treatment Expenditure (US$ billions [95% UI])	2050 total Maintenance Expenditure (US$ billions [95% UI])	2050 total Rehabilitative Care Expenditure (US$ billions [95% UI])	Total % change 2021–2050 (% [95% UI])
**2050 (current‐usage scenario)**						
Global	174.9 (137.4–212.3)	113.0 (81.4–144.6)	10.6 (4.6–16.7)	23.7 (14.0–33.5)	27.5 (11.1–44.0)	4.0% (−22.6%–39.8%)
GBD super‐region						
Central Europe, Eastern Europe and Central Asia	1.7 (0.6–2.9)	0.9 (0.0–1.7)	0.2 (0.0–0.5)	0.5 (0.0–1.0)	0.5 (0.0–1.0)	−11.5% (−64.9%–123.2%)
High‐income	137.0 (102.4–171.6)	91.6 (62.1–121.1)	7.4 (2.1–12.6)	19.9 (4.9–35.0)	19.9 (4.9–35.0)	5.9% (−25.3%–50.1%)
Latin America and Caribbean	3.6 (0.8–6.4)	2.1 (0.0–4.7)	0.4 (0.0–1.0)	0.7 (0.0–1.5)	0.7 (0.0–1.5)	11.3% (−64.7%–251.6%)
North Africa and Middle East	3.9 (1.1–6.7)	1.9 (0.0–4.1)	0.5 (0.0–1.1)	1.1 (0.0–2.5)	1.1 (0.0–2.5)	29.1% (−51.7%–245.2%)
South Asia	1.1 (0.0–6.0)	0.5 (0.0–3.9)	0.1 (0.0–1.1)	0.1 (0.0–1.1)	0.1 (0.0–1.1)	28.9% (−99.8%–77 823.5%)
Southeast Asia, East Asia and Oceania	26.3 (13.6–39.1)	15.2 (5.0–25.5)	2.0 (0.0–4.6)	5.1 (0.0–11.5)	5.1 (0.0–11.5)	−9.0% (−53.1%–76.6%)
Sub‐Saharan Africa	1.1 (0.0–2.5)	0.7 (0.0–1.9)	0.1 (0.0–0.3)	0.2 (0.0–0.6)	0.2 (0.0–0.6)	70.4% (−66.4%–765.0%)

Super‐region	2050 total Expenditure (US$ billions [95% UI])	2050 total Preventive, Diagnostic and Extraction Phase Expenditure (US$ billions [95% UI])	2050 total Active Treatment Expenditure (US$ billions [95% UI])	2050 total Maintenance Expenditure (US$ billions [95% UI])	2050 total Rehabilitative Care Expenditure (US$ billions [95% UI])	Total % change 2021–2050 (% [95% UI])
**2050 (expanded coverage scenario)**						
Global	500.6 (389.2–612.1)	254.9 (194.6–315.3)	23.6 (9.2–38.0)	151.2 (68.4–234.0)	70.9 (29.5–112.3)	197.8% (120.2%–302.8%)
GBD super‐region						
Central Europe, Eastern Europe and Central Asia	29.7 (21.7–37.6)	15.1 (10.0–20.2)	1.4 (0.4–2.3)	9.7 (4.3–15.0)	3.5 (0.7–6.3)	1414.7% (642.6%–2989.4%)
High‐income	148.1 (113.4–182.8)	97.5 (68.0–127.0)	7.8 (2.5–13.1)	22.0 (13.2–30.9)	20.7 (5.7–35.8)	14.5% (−18.2%–60.2%)
Latin America and Caribbean	43.5 (31.5–55.5)	22.6 (15.0–30.1)	1.9 (0.5–3.4)	14.3 (6.1–22.6)	4.7 (0.6–8.7)	1246.9% (457.7%–3152.5%)
North Africa and Middle East	53.9 (41.0–66.7)	26.3 (17.7–34.9)	2.5 (1.0–4.0)	18.4 (9.9–26.9)	6.7 (2.5–10.9)	1676.2% (768.6%–3532.3%)
South Asia	109.9 (10.4–209.5)	35.5 (0.0–81.3)	4.9 (0.0–17.6)	50.9 (0.0–130.4)	18.6 (0.0–55.2)	12511.1% (3.6%–1 535 410.7%)
Southeast Asia, East Asia and Oceania	66.2 (44.4–88.1)	34.9 (20.3–49.6)	3.7 (0.3–7.1)	17.6 (4.3–30.9)	10.0 (1.3–18.8)	128.7% (30.5%–301.0%)
Sub‐Saharan Africa	49.3 (27.9–70.8)	23.0 (5.7–40.4)	1.4 (0.0–3.1)	18.2 (7.0–29.5)	6.7 (1.2–12.2)	7238.5% (2094.9%–24 436.3%)

*Note:* Preventive, diagnostic and extraction phase care comprised consultations, radiographs, prophylaxis and extractions. Active periodontal treatment comprised oral hygiene instructions, supra‐ and subgingival instrumentation and periodontal surgery. Maintenance corresponded to supportive periodontal care. Rehabilitative phase comprised implant placement, implant‐supported restorations and the fabrication and repair of fixed or removable prostheses.

**TABLE 2:** Global and super‐region per‐capita periodontal expenditure (2025 US$) in 2021, with projections to 2050 under current‐usage and expanded coverage scenarios.

Super‐region	2021 per‐capita Expenditure (US$ [95% UI])	2021 per‐capita Preventive, Diagnostic and Extraction Phase Expenditure (US$ [95% UI])	2021 per‐capita Active Treatment Expenditure (US$ [95% UI])	2021 per‐capita Maintenance Expenditure (US$ [95% UI])	2021 per‐capita Rehabilitative Care Expenditure (US$ [95% UI])
**2021**					
Global	21.3 (17.0–25.7)	14.2 (10.5–17.9)	1.2 (0.5–1.9)	2.7 (1.7–3.8)	3.2 (1.2–5.2)
GBD super‐region					
Central Europe, Eastern Europe, and Central Asia	4.7 (1.6‐7.8)	2.4 (0.0‐4.9)	0.5 (0.0‐1.3)	0.6 (0.0‐1.5)	1.2 (0.0‐2.6)
High‐income	118.6 (90.0–147.1)	80.6 (56.1–105.1)	6.1 (1.9–10.3)	15.2 (8.2–22.1)	16.7 (4.4–28.9)
Latin America and Caribbean	5.4 (0.9–10.0)	3.5 (0.0–7.8)	0.5 (0.0–1.4)	0.6 (0.0–1.5)	0.9 (0.0–1.9)
North Africa and Middle East	4.9 (1.6–8.2)	2.7 (0.0–5.4)	0.5 (0.0–1.2)	0.5 (0.0–1.3)	1.2 (0.0–2.7)
South Asia	0.5 (0.0–2.7)	0.3 (0.0–2.1)	0.0 (0.0–0.6)	0.1 (0.0–1.2)	0.0 (0.0–0.4)
Southeast Asia, East Asia and Oceania	13.3 (7.2–19.3)	8.5 (3.4–13.6)	0.9 (0.0–2.1)	1.7 (0.4–3.0)	2.2 (0.0–5.0)
Sub‐Saharan Africa	0.6 (0.0–1.3)	0.4 (0.0–1.0)	0.0 (0.0–0.1)	0.1 (0.0–0.2)	0.1 (0.0–0.3)

Super‐region	2050 per‐capita Expenditure (US$ [95% UI])	2050 per‐capita Preventive, Diagnostic and Extraction Phase Expenditure (US$ [95% UI])	2050 per‐capita Active Treatment Expenditure (US$ [95% UI])	2050 per‐capita Maintenance Expenditure (US$ [95% UI])	2050 per‐capita Rehabilitative Care Expenditure (US$ [95% UI])	Per‐capita % change 2021–2050 (% [95% UI])
**2050 (current‐usage scenario)**						
Global	18.4 (14.5–22.3)	11.9 (8.6–15.2)	1.1 (0.5–1.7)	2.5 (1.5–3.5)	2.9 (1.2–4.7)	−13.6 (−35.6–15.9)
GBD super‐region						
Central Europe, Eastern Europe and Central Asia	4.3 (1.6–7.0)	2.2 (0.0–4.4)	0.5 (0.0–1.2)	0.6 (0.0–1.4)	1.1 (0.0–2.5)	−8.5 (−63.7–130.3)
High‐income	121.7 (90.9–152.5)	81.4 (55.1–107.7)	6.6 (1.9–11.2)	16.0 (8.4–23.7)	17.7 (4.4–31.0)	2.6 (−27.7–45.5)
Latin America and Caribbean	5.1 (1.2–9.0)	3 (0.0–6.5)	0.5 (0.0–1.5)	0.6 (0.0–1.6)	0.9 (0.0–2.1)	−5.6 (−69.6–193.8)
North Africa and Middle East	4.4 (1.3–7.5)	2.1 (0.0–4.5)	0.5 (0.0–1.3)	0.6 (0.0–1.4)	1.2 (0.0–2.8)	−10.2 (−66.5–140.5)
South Asia	0.5 (0.0–2.9)	0.3 (0.0–1.9)	0.0 (0.0–0.5)	0.2 (0.0–1.7)	0.1 (0.0–0.5)	0.0 (−99.8–58969.4)
Southeast Asia, East Asia and Oceania	12.4 (6.3–18.5)	7.2 (2.3–12.1)	1.0 (0.0–2.2)	1.9 (0.4–3.3)	2.4 (0.0–5.3)	−6.8 (−52.3–82.2)
Sub‐Saharan Africa	0.5 (0.0–1.1)	0.3 (0.0–0.9)	0.0 (0.0–0.1)	0.1 (0.0–0.3)	0.1 (0.0–0.3)	−16.7 (−82.0–285.2)

Super‐region	2050 per‐capita Expenditure (US$ [95% UI])	2050 per‐capita Preventive, Diagnostic and Extraction Expenditure (US$ [95% UI])	2050 per‐capita Active Treatment Expenditure (US$ [95% UI])	2050 per‐capita Maintenance Expenditure (US$ [95% UI])	2050 per‐capita Rehabilitative Care Expenditure (US$ [95% UI])	Per‐capita % change 2021–2050 (% [95% UI])
**2050 (expanded coverage scenario)**						
Global	52.6 (40.8–64.4)	26.8 (20.5–33.1)	2.5 (1.0–4.0)	15.9 (7.2–24.6)	7.4 (3.1–11.7)	146.9 (82.6–233.9)
GBD Super‐region						
Central Europe, Eastern Europe and Central Asia	74.3 (54.3–94.3)	37.8 (25.1–50.5)	3.5 (1.1–5.9)	24.2 (10.9–37.6)	8.8 (1.7–15.9)	1480.9 (669.9–3145.9)
High‐income	131.6 (100.8–162.4)	86.6 (60.3–112.9)	6.9 (2.3–11.6)	19.6 (11.7–27.5)	18.4 (5.1–31.7)	11.0 (−20.7–55.3)
Latin America and Caribbean	61.1 (44.2–78.0)	31.7 (21.1–42.3)	2.7 (0.7–4.8)	20.1 (8.5–31.7)	6.6 (0.9–12.3)	1031.5 (369.7–2625.8)
North Africa and Middle East	60.0 (45.7–74.3)	29.3 (19.7–38.9)	2.8 (1.1–4.4)	20.5 (11.1–30.0)	7.4 (2.7–12.1)	1124.5 (495.7–2417.1)
South Asia	51.9 (4.9–98.9)	16.8 (0.0–38.4)	2.3 (0.0–8.3)	24.1 (0.0–61.6)	8.8 (0.0–26.0)	10280.0 (26.6–850640.5)
Southeast Asia, East Asia and Oceania	31.2 (20.8–41.6)	16.4 (9.5–23.3)	1.7 (0.1–3.3)	8.3 (2.0–14.5)	4.7 (0.6–8.8)	134.6 (33.3–312.9)
Sub‐Saharan Africa	23.0 (13.0–33.0)	10.7 (2.7–18.7)	0.6 (0.0–1.4)	8.5 (3.2–13.7)	3.1 (0.6–5.6)	3733.3 (1212.2–11098.5)

*Note:* Preventive, diagnostic and extraction phase care comprised consultations, radiographs, prophylaxis and extractions. Active periodontal treatment comprised oral hygiene instructions, supra‐ and subgingival instrumentation and periodontal surgery. Maintenance corresponded to supportive periodontal care. Rehabilitative phase comprised implant placement, implant‐supported restorations and the fabrication and repair of fixed or removable prostheses.

### 3.1 Periodontitis Expenditure in 2021

In 2021, global total expenditure on periodontitis was estimated at US$168.1 billion (95% uncertainty interval [UI]: 133.8‐202.4). Preventive, diagnostic and extraction phase accounted for the largest share of spending (US$112.2 billion), followed by maintenance (US$ 21.5 billion), rehabilitative care (US$ 24.9 billion) and active periodontal treatment (US$ 9.5 billion).

**FIGURE 2:**
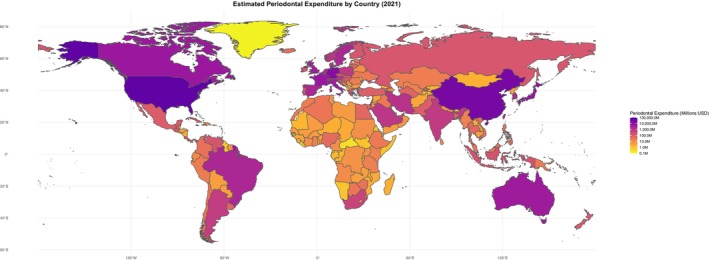
Map illustrating periodontal expenditure in 2021.

Substantial variation in total periodontal expenditure was observed across super‐regions. The High‐Income super‐region accounted for the largest share, with total expenditure of US$129.4 billion, whereas Sub‐Saharan Africa accounted for US$0.7 billion. At the country level, five countries accounted for more than two‐thirds of global expenditure in 2021: the United States (US$65.8 billion), China (US$26.5 billion), Germany (US$13.5 billion), Japan (US$8.4 billion) and France (US$5.8 billion) (Figure 2). Together, these countries represented 71.4% of global spending.

Global per‐capita expenditure in 2021 was US$21.32 (95% UI: 16.97–25.67), with marked disparities across super‐regions and countries. At the super‐region level, the highest per‐capita spending was observed in the High‐Income super‐region (US$118.56), while the lowest occurred in South Asia (US$0.47). At the country level, the highest per‐capita expenditure was observed in Sweden (US$238.06), the United States (US$197.92), Luxembourg (US$195.22), Australia (US$172.95) and Switzerland (US$172.93). More than half of all countries had per‐capita expenditure below US$1. The lowest per‐capita estimates were observed in Somalia (US$0.04), the Central African Republic (US$0.05), Rwanda (US$0.06), Burundi (US$0.06) and Madagascar (US$0.07).

### 3.2 Forecasted Periodontitis Expenditure to 2050 (Base, Current‐Usage Scenario)

**FIGURE 3:**
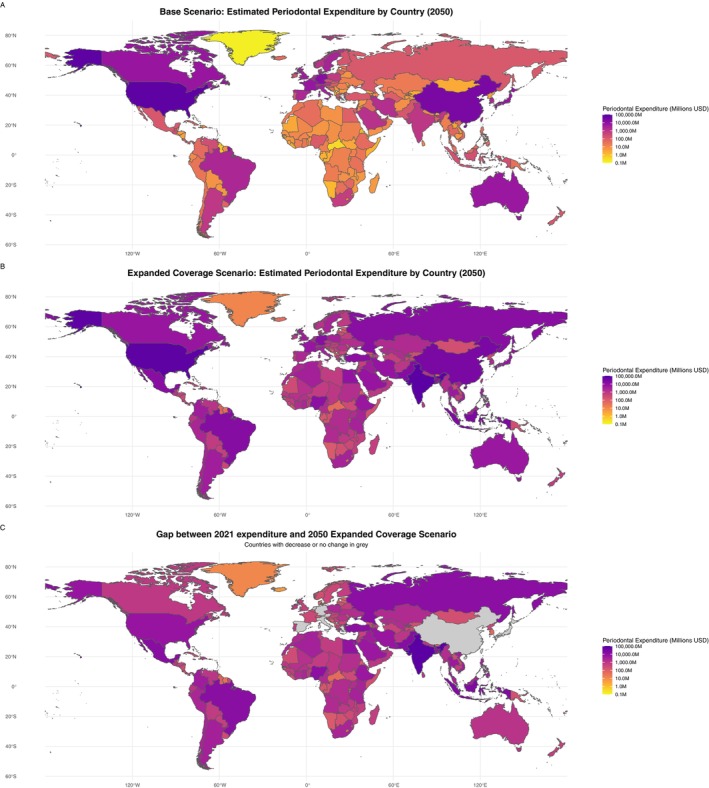
Maps illustrating periodontal expenditure in 2050 under the base scenario (A) and the expanded coverage scenario (B), and the gap between 2021 expenditure and 2050 expenditure under the expanded coverage scenario (C).

Under the base, current‐usage scenario–assuming that patterns of dental utilisation remain unchanged–global total expenditure on periodontitis was projected to increase by 4.2% to US$174.9 billion (95% UI: 137.4‐212.3) by 2050 (Figure 3A). Over the same period, the global number of individuals with severe periodontitis is projected to increase by 44.3% [20].

Projected trends in total expenditure varied substantially across super‐regions. Decreases in total spending were projected for Southeast Asia, East Asia and Oceania, as well as for Central Europe, Eastern Europe and Central Asia. In contrast, increases were projected for the High‐Income, Latin America, South Asia, North Africa and Middle East and Sub‐Saharan Africa super‐regions. At the country level, the highest total expenditure in 2050 was projected for the United States (US$72.2 billion), while the lowest was projected for Vanuatu (US$0.005 billion).

Under the current‐usage scenario, global per‐capita expenditure was projected to decrease by 14% to US$18.36 (95% UI: 14.43–22.29) by 2050. The super‐region with the highest projected per‐capita expenditure was the High‐Income super‐region (US$121.71), whereas the lowest was Sub‐Saharan Africa (US$0.53). At the country level, the highest per‐capita expenditure was projected for Sweden (US$237.13), while the lowest was projected for Somalia (US$0.04).

### 3.3 Forecasted Periodontitis Expenditure to 2050 (Expanded Coverage Scenario)

Under the expanded coverage scenario–in which access to periodontal care increases to 80% of the population by 2030–global total expenditure on periodontitis in 2050 was projected to reach US$500.6 billion (95% UI: 389.2‐612.1) (Figure 3B). This corresponds to a 198% increase compared with 2021 levels (Figure 3C).

**FIGURE 4:**
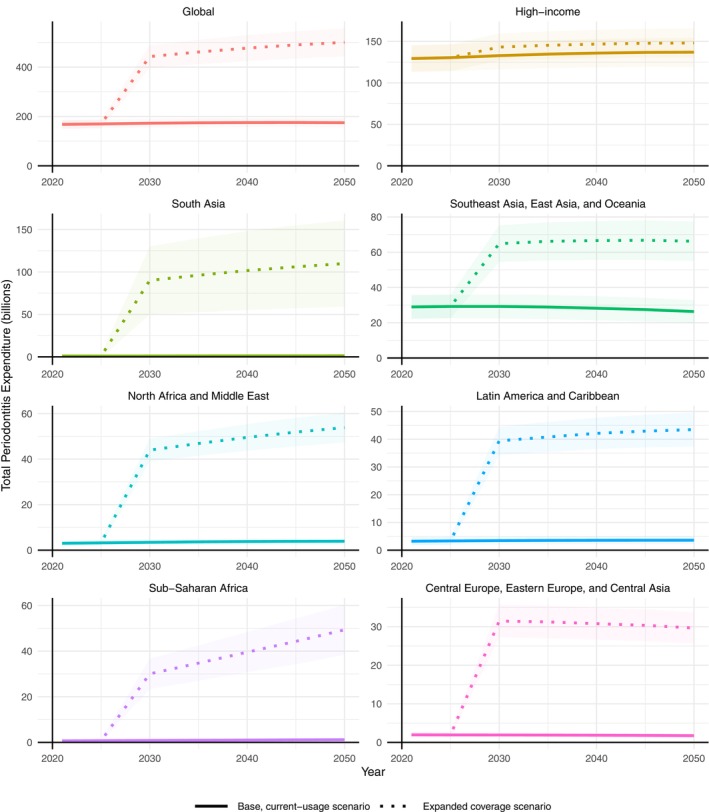
Periodontitis expenditure from 2021 to 2050 under both the base scenario and the expanded coverage scenario, shown globally and by super‐region.

Expenditure under the expanded coverage scenario was higher than under the base scenario in all super‐regions (Figure 4). The increase was relatively modest in the High‐Income super‐region (+15% to US$148 billion), but substantial in Southeast Asia, East Asia and Oceania (+129% to US$66.2 billion). All other super‐regions were projected to experience exponential increases in expenditure, ranging from +1247% in Latin America and the Caribbean to +12 511% in South Asia.

At the country level, the five countries with the highest periodontitis expenditure in 2050 differed markedly from those in 2021 (Results S1), with India, Indonesia and Brazil showing the largest absolute increases. Across all countries, the median absolute increase in expenditure was US$0.35 billion, corresponding to a median percentage increase of 9248.8% relative to 2021 (Figure 3C). The five countries with the largest absolute increases were India (+12 045%, US$84.5 billion), Indonesia (+10 646%, US$18.8 billion), Pakistan (+15 232%, US$14.9 billion), Mexico (+12 296%, US$12.7 billion) and Brazil (+516%, US$12.4 billion).

Under the expanded coverage scenario, global per‐capita expenditure was projected to increase by 147% to US$52.56 (95% UI: 40.86–64.26). The super‐region with the highest projected per‐capita expenditure was High‐Income (US$131.56), while the lowest was Sub‐Saharan Africa (US$22.98). At the country level, the highest per‐capita expenditure was projected for Qatar (US$326.00) and the lowest for Rwanda (US$5.03).

### 3.4 Sensitivity Analyses

Sensitivity analyses indicated that model outputs were robust to key structural assumptions. Reducing the expenditure cap from 75% to 50% of total dental expenditure decreased estimated global periodontal expenditure in 2021 by 7.7% (Results S2). Similarly, varying the allocation of the dentate population between health/gingivitis and stage I–II periodontitis from 50:50 to either 60:40 or 40:60 changed total expenditure by 4.5% (Results S3). The model was least sensitive to assumptions regarding the mean number of periodontal surgeries per patient: reversing the baseline assumption—such that Stage IV case types 3–4 (with fewer remaining teeth) received fewer surgeries than Stage III and Stage IV case types 1–2—altered global expenditure by only 2.1% (Results S4).

## 4 Discussion

In 2021, global total expenditure on periodontitis was estimated at US$168.1 billion (US$21.32 per capita) and is projected to reach US$174.9 billion (US$18.36 per capita) by 2050 under current levels of dental utilisation. If all countries were to extend periodontal care services to 80% of the population by 2030, total expenditure in 2050 would need to increase threefold to US$500.6 billion (US$52.56 per capita), corresponding to an annual financing gap of US$325.7 billion.

The projected stability in global total periodontitis expenditure under current utilisation contrasts sharply with the expected 44.3% increase in severe periodontitis prevalence over the same period, largely driven by Sub‐Saharan Africa (159 million additional cases) and South Asia (193 million additional cases) [20]. This apparent paradox is explained by demographic shifts: population growth and the resulting increase in severe periodontitis are concentrated in lower‐income countries with low periodontal expenditure, whereas higher‐income countries with greater spending show relative population and disease prevalence stagnation. In low‐income regions, per‐capita expenditure on periodontal care is already well below the global median, and maintaining current low levels of dental utilisation implies that the growing disease burden will translate primarily into greater unmet treatment need rather than increased spending. By contrast, the High‐Income super‐region and China, which together account for the majority of current global spending on periodontitis, are projected to experience stable or declining prevalence through 2050 [20]. These divergent trajectories highlight the financial and resource constraints that limit the global expansion of periodontal care worldwide.

### 4.1 Financing Gaps as a Barrier to Inclusion of Periodontal Care Under GOHAP

These findings have important implications for discussions on expanded oral health coverage and the implementation of the WHO GOHAP. The expanded coverage scenario modelled in this study does not represent an official WHO target for periodontal care. Rather, it serves as an illustrative stress test, representing a high‐access benchmark under prevailing care models, anchored to the GOHAP's stated ambition to increase access to oral health services globally from 23% to 80% by 2030. Applying this benchmark to periodontitis reveals a fundamental tension between two prevailing policy positions.

On one hand, the GOHAP does not explicitly include periodontitis within its illustrative package of universal oral health services, nor does it define treatment or prevalence targets for the condition, reflecting historical prioritisation and the limited evidence on population‐level effectiveness and scalable delivery models for periodontal care [21]. On the other hand, professional and stakeholder groups, including the European Federation of Periodontology, have called for a substantial expansion of preventive, diagnostic and therapeutic periodontal services within health systems [23].

Our findings quantify the structural trade‐offs between these positions. Continued omission of periodontal care from essential service frameworks, in the context of rising prevalence and population ageing [67], is likely to result in increasing unmet need, particularly in low‐ and middle‐income countries. Conversely, extending periodontal coverage to 80% of the population using prevailing treatment paradigms would require a threefold increase in global expenditure, generating a financing gap of US$325.7 billion annually.

Importantly, the scale and distribution of this projected financing gap underscore the constraints of expanding coverage within current delivery models. Because the GOHAP emphasises public financing as a means to promote equity and financial protection [24], closing such a gap would place substantial pressure on public budgets. This pressure is further compounded by the fact that the shortfall is disproportionately concentrated in super‐regions where disease burden and inequity are greatest and public spending capacity is lowest. Even with substantial Official Development Assistance, meeting these increases in public expenditure would be difficult, particularly in the context of declining assistance in recent years [68]. In several super‐regions, oral health may also be deprioritised due to competing health needs, including communicable, maternal, neonatal and nutritional diseases [69], as exemplified by Sub‐Saharan Africa, where life‐threatening conditions such as HIV, tuberculosis, malaria and neonatal mortality remain major public health concerns [70].

The central challenge, therefore, is not whether periodontitis should be addressed within global oral health strategies, but how it can be addressed sustainably. Continued exclusion of periodontitis from essential service frameworks under the GOHAP risks perpetuating rising unmet need, while uncritical expansion under existing delivery models entails substantial and potentially prohibitive financial trade‐offs between disease burden, access and expenditure.

### 4.2 Policy Directions

One possible approach to addressing the projected financing shortfall under the expanded coverage scenario is to re‐evaluate the scope of universal oral healthcare with respect to periodontitis.

From a prevention perspective, population‐level common risk factor approaches–such as policies targeting diabetes and smoking–have been proposed as cost‐effective strategies for the joint prevention of periodontitis and related comorbidities [71]. However, robust supporting evidence remains limited, with most recommendations based on expert opinion or consultative workshops [66]. High‐quality evaluations of both efficacy and cost‐effectiveness of public health approaches to periodontitis prevention are therefore urgently needed to inform future policy revisions.

For the management of established periodontitis, financial sustainability may require limiting publicly funded coverage to stage III‐IV periodontitis as part of essential care, where treatment needs and potential health impacts are greatest. In contrast, treatment of stage I‐II periodontitis, which accounts for the majority of cases according to epidemiological data [43], could remain primarily the responsibility of individuals. This approach is supported by evidence indicating that only a minority of mild‐to‐moderate cases progress to severe disease [72]. Nevertheless, this strategy carries potential risks, particularly that individuals may delay care until disease reaches more advanced stages. A phased approach, whereby service coverage is gradually expanded to include milder disease categories, may therefore represent a more balanced alternative.

Complementary strategies could focus on maximising the value of existing resources through payment reforms and innovative financing and procurement models. Payment reforms may include performance‐based payment systems [73] and a shift toward greater reliance on publicly funded healthcare models rather than inefficient private insurance arrangements [74]. In parallel, task‐shifting and expanded skill mix—including enhanced roles for dental hygienists, dental nurses and other allied health professionals in preventive and early periodontal care—offer promising opportunities to improve access, sustainability and efficiency. Regional approaches to financing and procurement may also contribute to addressing funding constraints. These could include identifying shared financing and payment barriers across super‐regions, establishing partnerships with regional development banks, and implementing pooled purchasing and procurement mechanisms, as demonstrated by the Africa Medical Supplies Platform [75]. Such coordinated regional strategies may be particularly effective in mitigating persistent financing gaps in regions such as Sub‐Saharan Africa.

### 4.3 Comparisons With Existing Research

The estimated global expenditure on periodontitis of US$168.1 billion in 2021 is broadly consistent with the estimate reported by Pattamatta et al. (2024) [65], who calculated global direct treatment costs of US$186 billion in 2019. Their estimate, however, was based on an informal extrapolation from total dental expenditure, using the proportion of DALYs attributable to periodontitis relative to all oral conditions. In addition, the absence of regional and country‐level estimates in their analysis precludes detailed direct comparison with the present study.

Botelho et al. [18] reported country‐level estimates of periodontal expenditure, although restricted to Europe and the United States. The estimates generated in the present study were substantially higher. For example, periodontal expenditure in the United States was estimated at US$3.49 billion by Botelho et al. [18] compared with US$65.8 billion in the present analysis; for Germany, the corresponding figures were €6.26 billion (US$7.30 billion) versus US$13.5 billion. These discrepancies are likely attributable to the broader scope of procedures included here, which encompasses surgical treatment, maintenance and rehabilitative care excluded in Botelho et al. [18]. Additional methodological differences also contribute. Specifically, Botelho et al. [18] applied a fixed proportion of total dental expenditure (3.45%) to approximate periodontal spending, whereas the current study modelled treatment needs based on population dynamics and periodontitis prevalence, resulting in more granular and context‐specific estimates.

To date, no previous studies have estimated periodontitis expenditure across all global regions, super‐regions and countries, nor forecasted future expenditure through to 2050. As a result, further comparisons with existing literature are not currently possible.

### 4.4 Limitations

Some limitations should be acknowledged. Although primary data on procedure costs and their distribution were collected for countries representing approximately 89% of global dental expenditure, extrapolation was required for the remaining countries. The analysis also relied on prevalence forecasts for 2050 from the GBD 2021 study, which are model‐based; consequently, and more broadly, the limitations inherent to GBD 2021 apply to the present analysis. Because this study focused exclusively on direct costs associated with periodontitis, the overall economic burden is likely underestimated, as indirect costs (i.e., productivity losses due to periodontitis and periodontal care) were not included. In addition, orthodontic and caries‐related interventions, which are frequently delivered alongside periodontal care [41, 76, 77], were not considered.

Furthermore, dental utilisation patterns, treatment pathways and the distribution of patients across case scenarios are not consistently well‐characterised at the country level. This limited empirical granularity, together with variability in procedure costs and epidemiological projections, contributed to the wide uncertainty intervals observed for some results, particularly at the country level. However, one‐way sensitivity analyses varying the distribution of clinical case scenarios, periodontal care statuses and periodontal care regimens indicated that the model is relatively robust to alternative structural assumptions. Specifically, the decision to evenly allocate the dentate population without severe periodontitis between health/gingivitis and stage I/II periodontitis could overestimate treatment costs if a substantial proportion of stage I/II periodontitis cases remain underdiagnosed; nevertheless, sensitivity analyses showed that this assumption does not materially affect the study's central conclusions.

Preventive efficacy was not modelled because of the absence of reliable population‐level evidence. Although the cost‐effectiveness of expanded periodontal coverage underpins the recommendations of the EFP‐commissioned White Paper [23], the transition probabilities applied in that framework—intended to represent the effectiveness of professional interventions in improving periodontal health—are not derived from population‐based data, where effectiveness may be lower than in convenience samples. If preventive effects of professional periodontal care were achieved at scale, projected expenditure for 2050 under the expanded coverage scenario may be overestimated. At the same time, expanding preventive services—often operationalised as increased screening—could initially increase active treatment demand, leading to higher short‐term costs before longer‐term benefits emerge. Moreover, the expanded coverage scenario itself may be conservative: if increased access is driven primarily by active treatment rather than maintenance, financing gaps could be larger and arise earlier than estimated.

Procedure costs were scaled by nominal GDP per capita rather than purchasing power parity (PPP) to reflect the implications of expanded coverage for national health budgets. This choice may potentially understate the real economic costs of periodontitis in low‐income regions; however, it does not affect the estimated financing gap when interpreted relative to each country's national health budget.

Despite these limitations, the study provides a transparent and reproducible framework for estimating global expenditure on periodontitis, addressing a major research gap and offering evidence to inform policy discussions. The applicability of this framework may extend beyond periodontology, as it can also be adapted to estimate expenditure for other human diseases.

### 4.5 Future Research

Future improvements in estimates of the economic impact of periodontitis should build on addressing the limitations identified above, particularly through the generation of currently missing data to further refine the accuracy of the Monte Carlo model employed in this study.

From a broader perspective, the present findings underscore that meaningful inclusion of periodontitis within the GOHAP universal oral health package would require a substantial reorientation of population‐level periodontitis management. To support this transition, robust economic evaluations of public health strategies for periodontitis prevention–including common risk factor approaches within the broader context of multi‐morbidity–are urgently needed. Existing evidence remains sparse and methodologically heterogeneous [78]. Formal allocative efficiency analyses that incorporate health outcomes and comparative effectiveness are particularly required. In the absence of such evidence, periodontitis may continue to be excluded from future definitions of universal oral health services, leaving its substantial global burden inadequately addressed.

More broadly, current strategies for the prevention, diagnosis and treatment of periodontitis are predominantly professional‐based and, therefore, time‐ and resource‐consuming. Improved understanding of disease etiopathogenesis is necessary to enable the development of cost‐effective pharmacologic preventive and therapeutic interventions. Advances in diagnostics may also facilitate earlier identification of individuals at higher risk of progression to severe disease, thereby improving resource allocation.

## 5 Conclusions

Periodontitis management currently imposes a substantial and unequally distributed economic impact worldwide. Under current utilisation patterns, projected increases in prevalence by 2050 are expected to translate primarily into unmet need rather than increased spending. Conversely, inclusion of periodontitis at GOHAP‐level coverage (80% of the population) under prevailing models of care would require a threefold increase in global periodontal expenditure, highlighting the structural tension between access and affordability. Addressing this challenge will likely require a fundamental rethinking of periodontal care delivery. Strategies that shift from predominantly professional‐led interventions toward common risk factor prevention within the context of multimorbidity, more targeted diagnosis and novel pharmacological therapies may offer more scalable and cost‐effective pathways to reducing the future burden of periodontitis.

## Author Contributions

D.Y.C. contributed to study design, data collection, analysis and interpretation, and manuscript drafting. R.D.B. and J.R.H.T. contributed to study design, data analysis and interpretation, and critically revised the manuscript. G.G.N. contributed to study design, data analysis and interpretation, and critically revised the manuscript. M.R. contributed to study conception and design, data analysis and interpretation, and manuscript drafting.

## Funding

This work was self funded.

## Conflicts of Interest

The authors declare no conflicts of interest related to this study, which was self‐funded. Mario Romandini serves as Editor‐in‐Chief and Gustavo Nascimento as Associate Editor of the *Journal of Periodontal Research*, and are also authors of this article. In accordance with Wiley's standard policies for submissions by Editors, they were excluded from the editorial decision‐making and remained blinded throughout the peer review process, with another journal Editor designated as acting Editor‐in‐Chief.

## Supporting information


**Methods S1:** R code for Monte Carlo model (please see Github repository for full code).
**Methods S2:** Detailed search strategy for country‐level expenditure.
**Methods S3**: Search results for country‐level expenditure.
**Methods S4**: Search results for country‐level procedure charges.
**Methods S5**: Monte Carlo standard errors for super‐region total periodontal expenditure estimates (2021).
**Methods S6**: Monte Carlo simulation structure.
**Methods S7**: Validation for Monte Carlo simulation model structure based on known periodontal treatment expenditure.
**Methods S8**: Diagnostics for extrapolation of prophylaxis cost.
**Methods S9**: Diagnostics for extrapolation of procedure costs.
**Methods S10**: Validation for Monte Carlo simulation model structure based on total dental expenditure.
**Results S1**: Country‐level total expenditure (A) and per capita expenditure (B) from 2021 to 2050 under the base and WHO target scenarios, highlighting the five countries with the highest expenditure in 2050 under the WHO target scenario.
**Results S2**: Sensitivity analysis of global and super‐regional total periodontal expenditure in 2021 under alternative expenditure cap assumptions.
**Results S3**: Sensitivity analysis of 2021 global and super‐regional periodontal expenditure under alternative health/gingivitis allocation assumptions.
**Results S4**: Sensitivity analysis of 2021 global and super‐regional periodontal expenditure under alternative assumptions on the number of periodontal surgeries by disease stage.

## Data Availability

The data that support the findings of this study are available from the corresponding author upon reasonable request.

## Appendix 1 Total Periodontitis Expenditure (2025 US$ Billions) in 2021, With Projections to 2050, Across 21 Regions and 204 Countries

**Table:**

Country/Region	Super‐region	Region	2021 Total expenditure (US$ billions [95% UI])	2050 Total expenditure (current‐usage scenario; US$ billions [95% UI])	Total % change 2021–2050 (current‐usage scenario; % [95% UI])	2050 Total expenditure (expanded coverage scenario; US$ billions [95% UI])	Total % change 2021–2050 (expanded coverage scenario; % [95% UI])	Dental utilisation scenario
**Global**	**NA**	**NA**	**168.099 (133.799–202.399)**	**174.857 (137.420–212.295)**	**4% (−23%–40%)**	**500.613 (389.168–612.058)**	**198% (120–303%)**	**NA**
**Central Europe, Eastern Europe and Central Asia**	**NA**	**Central Europe, Eastern Europe and Central Asia**	**1.958 (0.665–3.251)**	**1.733 (0.610–2.856)**	−**12% (**−**65–123%)**	**29.659 (21.695–37.623)**	**1415% (643–2989%)**	**NA**
**Central Asia**	**Central Asia**	**Central Europe, Eastern Europe and Central Asia**	**0.144 (0.010–0.290)**	**0.187 (0.010–0.378)**	**30% (**−**69–446%)**	**6.127 (3.989–8.266)**	**4146% (1355–12292%)**	**NA**
Armenia	Central Asia	Central Europe, Eastern Europe and Central Asia	0.030 (0.010–0.072)	0.030 (0.010–0.076)	1% (−88–731%)	0.147 (0.023–0.270)	396% (−6–2528%)	mid
Azerbaijan	Central Asia	Central Europe, Eastern Europe and Central Asia	0.007 (0.010–0.034)	0.008 (0.010–0.043)	19% (**−**100–34593%)	0.732 (0.193–1.270)	10376% (102–543368%)	low
Georgia	Central Asia	Central Europe, Eastern Europe and Central Asia	0.002 (0.010–0.012)	0.002 (0.010–0.011)	**−**3% (**−**100–63011%)	0.181 (0.037–0.325)	8456% (**−**35–1123925%)	low
Kazakhstan	Central Asia	Central Europe, Eastern Europe and Central Asia	0.017 (0.010–0.090)	0.022 (0.010–0.103)	24% (**−**100–34535%)	1.916 (0.822–3.010)	10921% (66–730833%)	low
Kyrgyzstan	Central Asia	Central Europe, Eastern Europe and Central Asia	0.002 (0.010–0.013)	0.002 (0.010–0.015)	37% (**−**100–1181116%)	0.229 (0.010–0.549)	14486% (**−**88–17970609%)	low
Mongolia	Central Asia	Central Europe, Eastern Europe and Central Asia	0.002 (0.010–0.009)	0.002 (0.010–0.014)	35% (**−**100–82969%)	0.212 (0.030–0.394)	11982% (41–1033799%)	low
Tajikistan	Central Asia	Central Europe, Eastern Europe and Central Asia	0.002 (0.010–0.020)	0.004 (0.010–0.028)	53% (**−**100–4825418%)	0.356 (0.010–0.872)	15302% (**−**94–40707209%)	low
Turkmenistan	Central Asia	Central Europe, Eastern Europe and Central Asia	0.067 (0.010–0.153)	0.097 (0.010–0.227)	45% (**−**77–820%)	0.504 (0.177–0.831)	650% (78–3064%)	mid
Uzbekistan	Central Asia	Central Europe, Eastern Europe and Central Asia	0.015 (0.010–0.090)	0.020 (0.010–0.114)	31% (**−**100–103707%)	1.850 (0.256–3.444)	11869% (**−**10–1593994%)	low
**Central Europe**	**Central Europe**	**Central Europe, Eastern Europe and Central Asia**	**1.498 (0.352–2.644)**	**1.275 (0.293–2.258)**	**−15% (–71–152%)**	**8.598 (6.370–10.826)**	**474% (156–1188%)**	**NA**
Albania	Central Europe	Central Europe, Eastern Europe and Central Asia	0.002 (0.010–0.008)	0.002 (0.010–0.008)	5% (**−**100–45215%)	0.150 (0.029–0.271)	9673% (19–803163%)	low
Bosnia and Herzegovina	Central Europe	Central Europe, Eastern Europe and Central Asia	0.037 (0.010–0.091)	0.032 (0.010–0.079)	**−**13% (−89–614%)	0.155 (0.031–0.279)	323% (**−**22–2190%)	mid
Bulgaria	Central Europe	Central Europe, Eastern Europe and Central Asia	0.005 (0.010–0.026)	0.004 (0.010–0.019)	**−**25% (−100–16665%)	0.354 (0.135–0.574)	6473% (32–327584%)	low
Croatia	Central Europe	Central Europe, Eastern Europe and Central Asia	0.088 (0.010–0.208)	0.072 (0.010–0.174)	**−**19% (−89–493%)	0.334 (0.125–0.543)	280% (**−**15–1611%)	mid
Czechia	Central Europe	Central Europe, Eastern Europe and Central Asia	0.234 (0.010–0.534)	0.207 (0.010–0.472)	**−**11% (**−**86–442%)	1.025 (0.479–1.572)	338% (9–1657%)	mid
Hungary	Central Europe	Central Europe, Eastern Europe and Central Asia	0.134 (0.010–0.298)	0.114 (0.010–0.254)	**−**15% (**−**85–381%)	0.622 (0.292–0.953)	364% (22–1660%)	mid
Montenegro	Central Europe	Central Europe, Eastern Europe and Central Asia	0.000 (0.010–0.002)	0.000 (0.010–0.002)	**−**5% (**−**100–26172%)	0.040 (0.013–0.068)	8563% (67–449736%)	low
North Macedonia	Central Europe	Central Europe, Eastern Europe and Central Asia	0.001 (0.010–0.007)	0.001 (0.010–0.007)	**−**1% (**−**100–52680%)	0.119 (0.027–0.212)	8612% (**−**4–791315%)	low
Poland	Central Europe	Central Europe, Eastern Europe and Central Asia	0.811 (0.010–1.883)	0.685 (0.010–1.602)	**−**15% (−87–454%)	3.304 (1.410–5.198)	307% (**−**4–1620%)	mid
Romania	Central Europe	Central Europe, Eastern Europe and Central Asia	0.019 (0.010–0.089)	0.015 (0.010–0.072)	**−**20% (**−**100–16309%)	1.325 (0.529–2.121)	6924% (59–310128%)	low
Serbia	Central Europe	Central Europe, Eastern Europe and Central Asia	0.006 (0.010–0.030)	0.005 (0.010–0.027)	**−**12% (**−**100–22996%)	0.499 (0.145–0.854)	7895% (55–413207%)	low
Slovakia	Central Europe	Central Europe, Eastern Europe and Central Asia	0.107 (0.010–0.247)	0.093 (0.010–0.215)	**−**13% (**−**86–450%)	0.458 (0.207–0.709)	327% (4–1653%)	mid
Slovenia	Central Europe	Central Europe, Eastern Europe and Central Asia	0.053 (0.010–0.124)	0.044 (0.010–0.103)	**−**17% (**−**87–450%)	0.212 (0.094–0.329)	299% (**−**6–1601%)	mid
Eastern Europe	Eastern Europe	Central Europe, Eastern Europe and Central Asia	0.316 (0.010–0.896)	0.270 (0.010–0.779)	**−**14% (**−**94–1086%)	14.934 (7.593–22.275)	4626% (607–31496%)	NA
Belarus	Eastern Europe	Central Europe, Eastern Europe and Central Asia	0.008 (0.010–0.040)	0.007 (0.010–0.034)	**−**16% (**−**100–20691%)	0.614 (0.203–1.025)	7437% (47–387349%)	low
Estonia	Eastern Europe	Central Europe, Eastern Europe and Central Asia	0.030 (0.010–0.068)	0.027 (0.010–0.061)	**−**10% (**−**85–441%)	0.131 (0.059–0.203)	337% (9–1655%)	mid
Latvia	Eastern Europe	Central Europe, Eastern Europe and Central Asia	0.039 (0.010–0.091)	0.027 (0.010–0.063)	**−**32% (**−**90–359%)	0.128 (0.052–0.204)	228% (**−**24–1315%)	mid
Lithuania	Eastern Europe	Central Europe, Eastern Europe and Central Asia	0.065 (0.010–0.150)	0.057 (0.010–0.131)	**−**14% (**−**86–448%)	0.271 (0.119–0.422)	313% (1–1593%)	mid
Republic of Moldova	Eastern Europe	Central Europe, Eastern Europe and Central Asia	0.002 (0.010–0.011)	0.002 (0.010–0.009)	**−**12% (**−**100–100236%)	0.149 (0.004–0.294)	8386% (**−**47–1363141%)	low
Russian Federation	Eastern Europe	Central Europe, Eastern Europe and Central Asia	0.149 (0.010–0.709)	0.134 (0.010–0.626)	**−**10% (**−**100–17029%)	11.968 (4.802–19.133)	7916% (80–357620%)	low
Ukraine	Eastern Europe	Central Europe, Eastern Europe and Central Asia	0.022 (0.010–0.124)	0.018 (0.010–0.106)	**−**20% (**−**100–66104%)	1.673 (0.149–3.197)	7426% (**−**28–784055%)	low
**High‐income**	**NA**	**High‐income**	**129.381 (98.186–160.577)**	**136.998 (102.402–171.594)**	**6% (−25–50%)**	**148.086 (113.359–182.812)**	**14% (−18–60%)**	**NA**
**Australasia**	**Australasia**	**High‐income**	**4.588 (2.726–6.449)**	**5.740 (3.389–8.092)**	**25% (−30–123%)**	**6.273 (3.900–8.646)**	**37% (−21–138%)**	**NA**
Australia	Australasia	High‐income	4.461 (2.607–6.315)	5.604 (3.259–7.949)	26% (**−**30–127%)	5.604 (3.259–7.949)	26% (**−**30–127%)	high
New Zealand	Australasia	High‐income	0.127 (0.010–0.288)	0.136 (0.010–0.312)	8% (**−**82–556%)	0.669 (0.306–1.032)	428% (33–1999%)	mid
**High‐income Asia Pacific**	**High‐income Asia Pacific**	**High‐income**	**10.688 (7.007‐14.369)**	**9.130 (6.116–12.144)**	**−15% (−47–38%)**	**10.629 (7.546–13.713)**	**−1% (−37–56%)**	**NA**
Brunei Darussalam	High‐income Asia Pacific	High‐income	0.001 (0.010–0.004)	0.001 (0.010–0.005)	29% (**−**99–19290%)	0.096 (0.053–0.139)	10958% (183–431452%)	low
Japan	High‐income Asia Pacific	High‐income	8.366 (4.847–11.886)	6.654 (3.888–9.419)	**−**20% (**−**56–44%)	6.654 (3.888–9.419)	**−**20% (**−**56–44%)	high
Republic of Korea	High‐income Asia Pacific	High‐income	2.072 (1.034–3.110)	2.124 (1.003–3.245)	3% (**−**50–112%)	2.124 (1.003–3.245)	3% (**−**50–112%)	high
Singapore	High‐income Asia Pacific	High‐income	0.249 (0.010–0.545)	0.351 (0.010–0.779)	41% (**−**74–673%)	1.756 (0.981–2.531)	605% (98–2409%)	mid
**High‐income North America**	**High‐income North America**	**High‐income**	**71.069 (41.273–100.866)**	**78.272 (44.874–111.671)**	**10% (−39–100%)**	**78.284 (44.886–111.683)**	**10% (−39–100%)**	**NA**
Canada	High‐income North America	High‐income	5.231 (2.503–7.960)	6.040 (2.900–9.180)	15% (**−**45–141%)	6.040 (2.900–9.180)	15% (**−**45–141%)	high
Greenland	High‐income North America	High‐income	0.000 (0.010–0.001)	0.000 (0.010–0.001)	10% (**−**99–13024%)	0.012 (0.007–0.018)	8913% (184–286112%)	low
United States of America	High‐income North America	High‐income	65.838 (36.167–95.510)	72.232 (38.981–105.483)	10% (**−**42–109%)	72.232 (38.981–105.483)	10% (**−**42–109%)	high
**Southern Latin America**	**Southern Latin America**	**High‐income**	**0.724 (0.010–1.581)**	**0.965 (0.010–2.232)**	**33% (−77–681%)**	**7.162 (4.080–10.244)**	**889% (181–3388%)**	**NA**
Argentina	Southern Latin America	High‐income	0.632 (0.010–1.479)	0.860 (0.010–2.117)	36% (**−**81–889%)	4.120 (1.264–6.975)	552% (44–2849%)	mid
Chile	Southern Latin America	High‐income	0.027 (0.010–0.121)	0.032 (0.010–0.145)	17% (**−**99–15695%)	2.697 (1.562–3.832)	9715% (216–305094%)	low
Uruguay	Southern Latin America	High‐income	0.065 (0.010–0.157)	0.073 (0.010–0.181)	13% (**−**86–786%)	0.346 (0.105–0.586)	436% (9–2533%)	mid
**Western Europe**	**Western Europe**	**High‐income**	**42.313 (34.093–50.532)**	**42.890 (34.816–50.965)**	**1% (−23–33%)**	**45.737 (37.626–53.849)**	**8% (−17–41%)**	**NA**
Andorra	Western Europe	High‐income	0.010 (0.005–0.015)	0.008 (0.004–0.012)	**−**21% (**−**63–68%)	0.008 (0.004–0.012)	**−**21% (**−**63–68%)	high
Austria	Western Europe	High‐income	1.016 (0.502–1.530)	0.978 (0.480–1.477)	**−**4% (**−**53–97%)	0.978 (0.480–1.477)	**−**4% (**−**53–97%)	high
Belgium	Western Europe	High‐income	1.416 (0.624–2.208)	1.588 (0.719–2.458)	12% (**−**49–145%)	1.588 (0.719–2.458)	12% (**−**49–145%)	high
Cyprus	Western Europe	High‐income	0.002 (0.010–0.007)	0.002 (0.010–0.007)	1% (**−**99–14789%)	0.136 (0.064–0.208)	8716% (141–322524%)	low
Denmark	Western Europe	High‐income	0.893 (0.364–1.423)	0.929 (0.383–1.474)	4% (**−**55–140%)	0.929 (0.383–1.474)	4% (**−**55–140%)	high
Finland	Western Europe	High‐income	0.158 (0.010–0.366)	0.163 (0.010–0.378)	3% (**−**84–565%)	0.782 (0.374–1.190)	395% (20–1936%)	mid
France	Western Europe	High‐income	5.789 (3.210–8.367)	6.099 (3.398–8.799)	5% (**−**44–97%)	6.099 (3.398–8.799)	5% (**−**44–97%)	high
Germany	Western Europe	High‐income	13.491 (7.362–19.620)	12.945 (7.061–18.828)	**−**4% (**−**50–82%)	12.945 (7.061–18.828)	**−**4% (**−**50–82%)	high
Greece	Western Europe	High‐income	0.172 (0.010–0.397)	0.149 (0.010–0.347)	**−**13% (**−**87–461%)	0.744 (0.302–1.186)	333% (3–1725%)	mid
Iceland	Western Europe	High‐income	0.040 (0.020–0.060)	0.045 (0.022–0.068)	13% (**−**45–132%)	0.045 (0.022–0.068)	13% (**−**45–132%)	high
Ireland	Western Europe	High‐income	0.158 (0.010–0.341)	0.186 (0.010–0.399)	18% (**−**77–498%)	1.013 (0.559–1.468)	541% (86–2108%)	mid
Israel	Western Europe	High‐income	0.911 (0.428–1.393)	1.357 (0.639–2.076)	49% (**−**30–215%)	1.357 (0.639–2.076)	49% (**−**30–215%)	high
Italy	Western Europe	High‐income	4.427 (1.264–7.591)	3.803 (1.086–6.521)	**−**14% (**−**69–136%)	3.803 (1.086–6.521)	**−**14% (**−**69–136%)	high
Luxembourg	Western Europe	High‐income	0.126 (0.065–0.186)	0.145 (0.076–0.213)	15% (**−**41–126%)	0.145 (0.076–0.213)	15% (**−**41–126%)	high
Malta	Western Europe	High‐income	0.011 (0.010–0.023)	0.010 (0.010–0.021)	**−**9% (**−**84–414%)	0.049 (0.025–0.073)	364% (23–1645%)	mid
Monaco	Western Europe	High‐income	0.000 (0.010–0.001)	NA	NA	NA	NA	low
Netherlands	Western Europe	High‐income	2.022 (1.006–3.038)	1.961 (1.003–2.918)	**−**3% (**−**52–95%)	1.961 (1.003–2.918)	−3% (**−**52–95%)	high
Norway	Western Europe	High‐income	0.931 (0.442–1.419)	1.141 (0.540–1.742)	23% (**−**42–158%)	1.141 (0.540–1.742)	23% (**−**42–158%)	high
Portugal	Western Europe	High‐income	0.193 (0.010–0.437)	0.157 (0.010–0.359)	**−**18% (**−**87–393%)	0.789 (0.347–1.231)	309% (3–1532%)	mid
San Marino	Western Europe	High‐income	0.004 (0.002–0.007)	NA	NA	NA	NA	high
Spain	Western Europe	High‐income	2.476 (1.350–3.601)	2.222 (1.227–3.217)	**−**10% (**−**53–70%)	2.222 (1.227–3.217)	**−**10% (**−**53–70%)	high
Sweden	Western Europe	High‐income	2.470 (1.412–3.527)	2.812 (1.645–3.980)	14% (**−**37–107%)	2.812 (1.645–3.980)	14% (**−**37–107%)	high
Switzerland	Western Europe	High‐income	1.543 (0.852–2.234)	1.676 (0.941–2.411)	9% (**−**42–103%)	1.676 (0.941–2.411)	9% (**−**42–103%)	high
United Kingdom	Western Europe	High‐income	4.055 (1.297–6.814)	4.515 (1.394–7.636)	11% (**−**58–194%)	4.515 (1.394–7.636)	11% (**−**58–194%)	high
**Latin America and Caribbean**	**NA**	**Latin America and Caribbean**	**3.229 (0.524–5.934)**	**3.595 (0.763–6.428)**	**11% (−65–252%)**	**43.491 (31.528–55.454)**	**1247% (458–3152%)**	**NA**
**Andean Latin America**	**Andean Latin America**	**Latin America and Caribbean**	**0.033 (0.010–0.128)**	**0.045 (0.010–0.179)**	**40% (−98–8858%)**	**4.241 (1.859–6.623)**	**12927% (549–261259%)**	**NA**
Bolivia (Plurinational State of)	Andean Latin America	Latin America and Caribbean	0.004 (0.010–0.027)	0.007 (0.010–0.050)	73% (**−**100–566625%)	0.683 (0.010–1.418)	16481% (**−**38–4405857%)	low
Ecuador	Andean Latin America	Latin America and Caribbean	0.009 (0.010–0.060)	0.011 (0.010–0.065)	24% (**−**100–181717%)	1.035 (0.075–1.994)	11237% (**−**60–3181051%)	low
Peru	Andean Latin America	Latin America and Caribbean	0.019 (0.010–0.097)	0.027 (0.010–0.142)	40% (**−**100–49514%)	2.523 (0.471–4.575)	12967% (110–812201%)	low
**Caribbean**	**Caribbean**	**Latin America and Caribbean**	**0.220 (0.010–0.443)**	**0.212 (0.011–0.414)**	**−3% (−76–289%)**	**2.600 (1.553–3.647)**	**1084% (296–3438%)**	**NA**
Antigua and Barbuda	Caribbean	Latin America and Caribbean	0.002 (0.010–0.004)	0.002 (0.010–0.005)	13% (**−**85–771%)	0.009 (0.003–0.015)	435% (11–2482%)	mid
Bahamas	Caribbean	Latin America and Caribbean	0.009 (0.010–0.020)	0.010 (0.010–0.025)	19% (**−**83–737%)	0.047 (0.018–0.076)	456% (27–2327%)	mid
Barbados	Caribbean	Latin America and Caribbean	0.004 (0.010–0.011)	0.004 (0.010–0.011)	0% (**−**89–859%)	0.020 (0.003–0.037)	372% (**−**22–2762%)	mid
Belize	Caribbean	Latin America and Caribbean	0.004 (0.010–0.010)	0.006 (0.010–0.016)	59% (**−**85–1627%)	0.028 (0.000–0.056)	682% (13–5310%)	mid
Bermuda	Caribbean	Latin America and Caribbean	0.000 (0.010–0.001)	0.000 (0.010–0.001)	**−**11% (–99–14539%)	0.013 (0.006–0.020)	7456% (85–309080%)	low
Cuba	Caribbean	Latin America and Caribbean	0.117 (0.010–0.328)	0.100 (0.010–0.283)	**−**15% (–93–1009%)	0.472 (0.010–0.968)	303% (**−**50–3133%)	mid
Dominica	Caribbean	Latin America and Caribbean	0.001 (0.010–0.002)	0.001 (0.010–0.002)	16% (**−**89–1112%)	0.004 (0.000–0.008)	456% (**−**17–3641%)	mid
Dominican Republic	Caribbean	Latin America and Caribbean	0.009 (0.010–0.042)	0.010 (0.010–0.049)	15% (**−**100–27589%)	0.902 (0.259–1.545)	10316% (105–530352%)	low
Grenada	Caribbean	Latin America and Caribbean	0.001 (0.010–0.003)	0.002 (0.010–0.004)	20% (**−**86–951%)	0.007 (0.001–0.014)	471% (2–3102%)	mid
Guyana	Caribbean	Latin America and Caribbean	0.001 (0.010–0.003)	0.001 (0.010–0.004)	18% (**−**100–30588%)	0.068 (0.024–0.112)	10142% (67–626728%)	low
Haiti	Caribbean	Latin America and Caribbean	0.002 (0.010–0.021)	0.003 (0.010–0.026)	32% (**−**100–19481808%)	0.316 (0.010–0.908)	14685% (**−**98–113619615%)	low
Jamaica	Caribbean	Latin America and Caribbean	0.025 (0.010–0.067)	0.027 (0.010–0.076)	8% (**−**91–1187%)	0.127 (0.010–0.261)	411% (**−**30–3638%)	mid
Puerto Rico	Caribbean	Latin America and Caribbean	0.005 (0.010–0.022)	0.004 (0.010–0.021)	**−**11% (**−**100–20394%)	0.371 (0.140–0.602)	7872% (91–332598%)	low
Saint Kitts and Nevis	Caribbean	Latin America and Caribbean	0.001 (0.010–0.003)	NA	NA	NA	NA	mid
Saint Lucia	Caribbean	Latin America and Caribbean	0.002 (0.010–0.006)	0.002 (0.010–0.007)	13% (**−**89–1043%)	0.012 (0.001–0.022)	435% (**−**16–3320%)	mid
Saint Vincent and the Grenadines	Caribbean	Latin America and Caribbean	0.001 (0.010–0.004)	0.001 (0.010–0.003)	**−**4% (**−**90–774%)	0.006 (0.001–0.012)	359% (**−**22–2609%)	mid
Suriname	Caribbean	Latin America and Caribbean	0.007 (0.010–0.017)	0.008 (0.010–0.021)	21% (**−**87–989%)	0.040 (0.006–0.074)	488% (**−**0–3376%)	mid
Trinidad and Tobago	Caribbean	Latin America and Caribbean	0.030 (0.010–0.070)	0.031 (0.010–0.077)	6% (**−**86–679%)	0.146 (0.049–0.244)	395% (7–2181%)	mid
United States Virgin Islands	Caribbean	Latin America and Caribbean	0.000 (0.010–0.001)	0.000 (0.010–0.001)	3% (**−**100–22782%)	0.011 (0.004–0.018)	8866% (95–413083%)	low
**Central Latin America**	**Central Latin America**	**Latin America and Caribbean**	**0.576 (0.010–1.188)**	**0.870 (0.010–1.804)**	**51% (−67–585%)**	**21.363 (11.043–31.683)**	**3610% (1054–11831%)**	**NA**
Colombia	Central Latin America	Latin America and Caribbean	0.034 (0.010–0.177)	0.048 (0.010–0.261)	39% (**−**100–63778%)	4.296 (0.737–7.855)	12465% (79–881102%)	low
Costa Rica	Central Latin America	Latin America and Caribbean	0.071 (0.010–0.174)	0.092 (0.010–0.235)	30% (**−**85–986%)	0.436 (0.101–0.771)	515% (19–3073%)	mid
El Salvador	Central Latin America	Latin America and Caribbean	0.003 (0.010–0.017)	0.003 (0.010–0.017)	**−**1% (**−**100–125679%)	0.274 (0.000–0.547)	9550% (**−**36–1464669%)	low
Guatemala	Central Latin America	Latin America and Caribbean	0.121 (0.010–0.316)	0.230 (0.010–0.638)	90% (**−**83–1989%)	1.103 (0.010–2.226)	812% (35–6045%)	mid
Honduras	Central Latin America	Latin America and Caribbean	0.003 (0.010–0.027)	0.005 (0.010–0.036)	66% (**−**100–4481681%)	0.498 (0.010–1.136)	16554% (**−**95–61369320%)	low
Mexico	Central Latin America	Latin America and Caribbean	0.103 (0.010–0.519)	0.139 (0.010–0.722)	35% (**−**100–44763%)	12.784 (3.366–22.202)	12296% (106–747182%)	low
Nicaragua	Central Latin America	Latin America and Caribbean	0.002 (0.010–0.014)	0.003 (0.010–0.022)	45% (**−**100–787135%)	0.292 (0.010–0.661)	13987% (**−**68–6169575%)	low
Panama	Central Latin America	Latin America and Caribbean	0.076 (0.010–0.179)	0.110 (0.010–0.260)	44% (**−**79–890%)	0.530 (0.192–0.867)	597% (56–3021%)	mid
Venezuela (Bolivarian Republic of)	Central Latin America	Latin America and Caribbean	0.163 (0.010–0.512)	0.240 (0.010–0.767)	47% (**−**93–3063%)	1.151 (0.010–2.892)	606% (**−**49–9632%)	mid
**Tropical Latin America**	**Tropical Latin America**	**Latin America and Caribbean**	**2.401 (0.010–5.024)**	**2.468 (0.010–5.131)**	**3% (−78–377%)**	**15.287 (9.824–20.750)**	**537% (102–1910%)**	**NA**
Brazil	Tropical Latin America	Latin America and Caribbean	2.397 (0.010–5.020)	2.462 (0.010–5.125)	3% (**−**78–378%)	14.773 (9.327–20.218)	516% (94–1855%)	mid
Paraguay	Tropical Latin America	Latin America and Caribbean	0.004 (0.010–0.021)	0.006 (0.010–0.030)	47% (**−**100–79920%)	0.515 (0.079–0.950)	13512% (37–1348025%)	low
**North Africa and Middle East**	**NA**	**North Africa and Middle East**	**3.032 (0.987–5.078)**	**3.914 (1.110–6.718)**	**29% (−52–245%)**	**53.864 (41.008–66.720)**	**1676% (769–3532%)**	**NA**
**North Africa and Middle East**	**North Africa and Middle East**	**North Africa and Middle East**	**3.032 (0.987–5.078)**	**3.914 (1.110–6.718)**	**29% (−52–245%)**	**53.864 (41.008–66.720)**	**1676% (769–3532%)**	**NA**
Afghanistan	North Africa and Middle East	North Africa and Middle East	0.003 (0.010–0.045)	0.008 (0.010–0.079)	186% (**−**100–9024526101%)	0.980 (0.010–3.573)	34227% (**−**100–132202483529%)	low
Algeria	North Africa and Middle East	North Africa and Middle East	0.026 (0.010–0.134)	0.039 (0.010–0.219)	50% (**−**100–75378%)	3.492 (0.712–6.272)	13369% (94–934361%)	low
Bahrain	North Africa and Middle East	North Africa and Middle East	0.043 (0.010–0.097)	0.068 (0.010–0.156)	59% (**−**74–867%)	0.332 (0.164–0.500)	676% (99–2929%)	mid
Egypt	North Africa and Middle East	North Africa and Middle East	0.058 (0.010–0.323)	0.082 (0.010–0.442)	42% (**−**100–80228%)	7.595 (1.287–13.902)	13016% (24–1384376%)	low
Iran (Islamic Republic of)	North Africa and Middle East	North Africa and Middle East	1.158 (0.010–2.713)	1.482 (0.010–3.657)	28% (**−**82–836%)	7.166 (1.843–12.488)	519% (33–2773%)	mid
Iraq	North Africa and Middle East	North Africa and Middle East	0.022 (0.010–0.127)	0.048 (0.010–0.281)	117% (**−**100–180071%)	4.363 (0.780–7.945)	19471% (68–2276456%)	low
Jordan	North Africa and Middle East	North Africa and Middle East	0.006 (0.010–0.033)	0.009 (0.010–0.051)	57% (**−**100–145328%)	0.821 (0.070–1.572)	14429% (0–2100443%)	low
Kuwait	North Africa and Middle East	North Africa and Middle East	0.156 (0.010–0.349)	0.204 (0.010–0.474)	30% (**−**79–698%)	0.964 (0.452–1.477)	517% (62–2261%)	mid
Lebanon	North Africa and Middle East	North Africa and Middle East	0.050 (0.010–0.132)	0.118 (0.010–0.321)	138% (**−**78–2445%)	0.577 (0.016–1.138)	1057% (71–7712%)	mid
Libya	North Africa and Middle East	North Africa and Middle East	0.005 (0.010–0.024)	0.007 (0.010–0.037)	51% (**−**100–51137%)	0.641 (0.162–1.119)	13493% (124–823974%)	low
Morocco	North Africa and Middle East	North Africa and Middle East	0.018 (0.010–0.103)	0.022 (0.010–0.136)	27% (**−**100–142020%)	2.128 (0.010–4.280)	11993% (**−**15–1717072%)	low
Oman	North Africa and Middle East	North Africa and Middle East	0.006 (0.010–0.029)	0.012 (0.010–0.055)	89% (**−**99–33087%)	1.007 (0.476–1.538)	16053% (301–650557%)	low
Palestine	North Africa and Middle East	North Africa and Middle East	0.001 (0.010–0.011)	0.003 (0.010–0.020)	89% (**−**100–4238904%)	0.239 (0.010–0.550)	17569% (**−**91–34609540%)	low
Qatar	North Africa and Middle East	North Africa and Middle East	0.011 (0.010–0.050)	0.014 (0.010–0.061)	26% (**−**99–17084%)	1.150 (0.622–1.678)	10367% (202–362887%)	low
Saudi Arabia	North Africa and Middle East	North Africa and Middle East	0.912 (0.010–1.993)	1.257 (0.010–2.774)	38% (**−**75–648%)	6.308 (3.255–9.362)	592% (92–2387%)	mid
Sudan	North Africa and Middle East	North Africa and Middle East	0.008 (0.010–0.078)	0.016 (0.010–0.140)	94% (**−**100–22864747%)	1.546 (0.010–4.051)	18924% (**−**97–126378985%)	low
Syrian Arab Republic	North Africa and Middle East	North Africa and Middle East	0.003 (0.010–0.032)	0.005 (0.010–0.043)	63% (**−**100–27296732%)	0.498 (0.010–1.260)	16027% (**−**99–218208269%)	low
Tunisia	North Africa and Middle East	North Africa and Middle East	0.006 (0.010–0.032)	0.008 (0.010–0.046)	28% (**−**100–86998%)	0.730 (0.064–1.397)	11798% (50–943633%)	low
Türkiye	North Africa and Middle East	North Africa and Middle East	0.090 (0.010–0.421)	0.121 (0.010–0.573)	34% (**−**99–25460%)	10.641 (4.119–17.164)	11730% (185–491168%)	low
United Arab Emirates	North Africa and Middle East	North Africa and Middle East	0.446 (0.010–1.015)	0.384 (0.010–0.876)	**−**14% (**−**86–424%)	1.804 (0.933–2.675)	304% (3–1480%)	mid
Yemen	North Africa and Middle East	North Africa and Middle East	0.005 (0.010–0.060)	0.007 (0.010–0.060)	58% (**−**100–276966012%)	0.881 (0.010–2.686)	19398% (**−**100–5026126700%)	low
**South Asia**	**NA**	**South Asia**	**0.872 (0.010–4.983)**	**1.124 (0.010–5.994)**	**29% (−100–77823%)**	**109.944 (10.430–209.458)**	**12511% (4–1535411%)**	**NA**
**South Asia**	**South Asia**	**South Asia**	**0.872 (0.010–4.983)**	**1.124 (0.010–5.994)**	**29% (−100–77823%)**	**109.944 (10.430–209.458)**	**12511% (4–1535411%)**	**NA**
Bangladesh	South Asia	South Asia	0.065 (0.010–0.410)	0.087 (0.010–0.615)	34% (**−**100–444709%)	8.643 (0.010–20.112)	13251% (**−**45–3244662%)	low
Bhutan	South Asia	South Asia	0.000 (0.010–0.003)	0.001 (0.010–0.005)	82% (**−**100–162154%)	0.085 (0.000–0.171)	17661% (58–1995711%)	low
India	South Asia	South Asia	0.701 (0.010–4.750)	0.876 (0.010–5.627)	25% (**−**100–343090%)	85.187 (0.010–182.149)	12045% (**−**66–4356148%)	low
Nepal	South Asia	South Asia	0.008 (0.010–0.080)	0.010 (0.010–0.085)	34% (**−**100–24322214%)	1.061 (0.010–2.705)	13966% (**−**99–225144980%)	low
Pakistan	South Asia	South Asia	0.098 (0.010–0.724)	0.150 (0.010–1.080)	54% (**−**100–1144634%)	14.967 (0.010–34.130)	15232% (**−**78–10631315%)	low
**Southeast Asia, East Asia and Oceania**	**NA**	**Southeast Asia, East Asia and Oceania**	**28.954 (15.813–42.095)**	**26.348 (13.619–39.076)**	**−9% (−53–77%)**	**66.228 (44.352–88.104)**	**129% (30–301%)**	**NA**
**East Asia**	**East Asia**	**Southeast Asia, East Asia and Oceania**	**28.562 (15.448–41.676)**	**25.872 (13.183–38.562)**	**−9% (−54–77%)**	**26.242 (13.528–38.956)**	**−8% (−53–79%)**	**NA**
China	East Asia	Southeast Asia, East Asia and Oceania	26.450 (13.391–39.509)	23.828 (11.195–36.461)	**−**10% (**−**56–86%)	23.828 (11.195–36.461)	**−**10% (**−**56–86%)	high
Democratic People's Republic of Korea	East Asia	Southeast Asia, East Asia and Oceania	0.003 (0.010–0.044)	0.003 (0.010–0.031)	**−**0% (**−**100–747703437%)	0.373 (0.010–1.171)	11747% (**−**100–6214575338%)	low
Taiwan (Province of China)	East Asia	Southeast Asia, East Asia and Oceania	2.109 (0.908–3.310)	2.041 (0.843–3.240)	**−**3% (**−**57–119%)	2.041 (0.843–3.240)	**−**3% (**−**57–119%)	high
**Oceania**	**Oceania**	**Southeast Asia, East Asia and Oceania**	**0.028 (0.010–0.097)**	**0.046 (0.010–0.162)**	**63% (−95–5114%)**	**0.292 (0.010–0.781)**	**926% (−45–19059%)**	**NA**
American Samoa	Oceania	Southeast Asia, East Asia and Oceania	0.000 (0.010–0.000)	0.000 (0.010–0.000)	71% (**−**100–70514%)	0.005 (0.001–0.009)	15790% (119–1151241%)	low
Cook Islands	Oceania	Southeast Asia, East Asia and Oceania	0.000 (0.010–0.000)	NA	NA	NA	NA	low
Fiji	Oceania	Southeast Asia, East Asia and Oceania	0.004 (0.010–0.012)	0.005 (0.010–0.015)	25% (**−**89–1342%)	0.032 (0.010–0.066)	627% (**−**6–5540%)	mid
Guam	Oceania	Southeast Asia, East Asia and Oceania	0.000 (0.010–0.001)	0.000 (0.010–0.001)	32% (**−**100–41345%)	0.015 (0.006–0.024)	11255% (75–737059%)	low
Kiribati	Oceania	Southeast Asia, East Asia and Oceania	0.000 (0.010–0.001)	0.000 (0.010–0.002)	90% (**−**99–67849%)	0.002 (0.010–0.008)	937% (**−**96–251689%)	mid
Marshall Islands	Oceania	Southeast Asia, East Asia and Oceania	0.000 (0.010–0.001)	0.000 (0.010–0.001)	41% (**−**95–3697%)	0.001 (0.010–0.004)	747% (**−**55–15984%)	mid
Micronesia (Federated States of)	Oceania	Southeast Asia, East Asia and Oceania	0.000 (0.010–0.001)	0.000 (0.010–0.001)	55% (**−**98–12322%)	0.002 (0.010–0.006)	781% (**−**82–42760%)	mid
Nauru	Oceania	Southeast Asia, East Asia and Oceania	0.000 (0.010–0.000)	NA	NA	NA	NA	mid
Niue	Oceania	Southeast Asia, East Asia and Oceania	0.000 (0.010–0.000)	NA	NA	NA	NA	low
Northern Mariana Islands	Oceania	Southeast Asia, East Asia and Oceania	0.000 (0.010–0.000)	0.000 (0.010–0.000)	7% (**−**100–71455%)	0.002 (0.000–0.004)	9249% (**−**12–996187%)	low
Palau	Oceania	Southeast Asia, East Asia and Oceania	0.000 (0.010–0.000)	NA	NA	NA	NA	mid
Papua New Guinea	Oceania	Southeast Asia, East Asia and Oceania	0.021 (0.010–0.089)	0.036 (0.010–0.152)	73% (**−**98–15651%)	0.209 (0.010–0.696)	891% (**−**81–52341%)	mid
Samoa	Oceania	Southeast Asia, East Asia and Oceania	0.001 (0.010–0.002)	0.001 (0.010–0.004)	75% (**−**94–4917%)	0.006 (0.010–0.018)	941% (**−**44–19218%)	mid
Solomon Islands	Oceania	Southeast Asia, East Asia and Oceania	0.001 (0.010–0.006)	0.002 (0.010–0.008)	61% (**−**100–84892%)	0.009 (0.010–0.038)	791% (**−**97–272442%)	mid
Tokelau	Oceania	Southeast Asia, East Asia and Oceania	0.000 (0.010–0.000)	NA	NA	NA	NA	low
Tonga	Oceania	Southeast Asia, East Asia and Oceania	0.000 (0.010–0.001)	0.001 (0.010‐0.002)	63% (**−**94–3976%)	0.003 (0.010–0.008)	871% (**−**38–15180%)	mid
Tuvalu	Oceania	Southeast Asia, East Asia and Oceania	0.000 (0.010–0.000)	NA	NA	NA	NA	mid
Vanuatu	Oceania	Southeast Asia, East Asia and Oceania	0.000 (0.010–0.000)	0.000 (0.010–0.001)	75% (**−**100–65770861693%)	0.005 (0.010–0.021)	16345% (**−**100–3594890856%)	low
**Southeast Asia**	**Southeast Asia**	**Southeast Asia, East Asia and Oceania**	**0.363 (0.010–1.200)**	**0.429 (0.010–1.421)**	**18% (−95–2989%)**	**39.694 (21.899–57.490)**	**10823% (946–114015%)**	**NA**
Cambodia	Southeast Asia	Southeast Asia, East Asia and Oceania	0.004 (0.010–0.030)	0.006 (0.010–0.044)	42% (**−**100–1729964%)	0.578 (0.010–1.405)	14403% (**−**83–12228376%)	low
Indonesia	Southeast Asia	Southeast Asia, East Asia and Oceania	0.176 (0.010–0.909)	0.202 (0.010–1.081)	15% (**−**100–46529%)	18.960 (2.974–34.945)	10646% (56–741947%)	low
Lao People's Democratic Republic	Southeast Asia	Southeast Asia, East Asia and Oceania	0.002 (0.010–0.013)	0.003 (0.010–0.019)	46% (**−**100–307060%)	0.272 (0.010–0.577)	13644% (**−**42–3280813%)	low
Malaysia	Southeast Asia	Southeast Asia, East Asia and Oceania	0.028 (0.010–0.139)	0.039 (0.010–0.179)	39% (**−**99–30591%)	3.417 (1.459–5.376)	12184% (118–692392%)	low
Maldives	Southeast Asia	Southeast Asia, East Asia and Oceania	0.000 (0.010–0.002)	0.000 (0.010–0.003)	52% (**−**100–58907%)	0.044 (0.014–0.074)	13260% (80–989778%)	low
Mauritius	Southeast Asia	Southeast Asia, East Asia and Oceania	0.001 (0.010–0.005)	0.001 (0.010–0.004)	−3% (**−**100–23987%)	0.083 (0.028–0.137)	8676% (58–488224%)	low
Myanmar	Southeast Asia	Southeast Asia, East Asia and Oceania	0.014 (0.010–0.125)	0.017 (0.010–0.116)	17% (**−**100–2069240%)	1.662 (0.010–3.831)	11542% (**−**96–30521064%)	low
Philippines	Southeast Asia	Southeast Asia, East Asia and Oceania	0.034 (0.010–0.245)	0.048 (0.010–0.328)	41% (**−**100–688348%)	4.281 (0.010–8.788)	12432% (**−**76–6658327%)	low
Seychelles	Southeast Asia	Southeast Asia, East Asia and Oceania	0.000 (0.010–0.000)	0.000 (0.010–0.001)	9% (**−**99–18665%)	0.009 (0.004–0.015)	9344% (159–344008%)	low
Sri Lanka	Southeast Asia	Southeast Asia, East Asia and Oceania	0.012 (0.010–0.067)	0.012 (0.010–0.061)	−0% (**−**100–57412%)	1.078 (0.211–1.946)	9142% (**−**24–1118735%)	low
Thailand	Southeast Asia	Southeast Asia, East Asia and Oceania	0.053 (0.010–0.289)	0.058 (0.010–0.298)	9% (**−**100–47738%)	5.108 (1.239–8.978)	9513% (6–872200%)	low
Timor‐Leste	Southeast Asia	Southeast Asia, East Asia and Oceania	0.000 (0.010–0.002)	0.000 (0.010–0.003)	60% (**−**100–2207378%)	0.045 (0.010–0.115)	16750% (**−**89–25964327%)	low
Viet Nam	Southeast Asia	Southeast Asia, East Asia and Oceania	0.038 (0.010–0.226)	0.044 (0.010–0.251)	14% (**−**100–106018%)	4.157 (0.186–8.128)	10753% (**−**27–1619264%)	low
**Sub‐Saharan Africa**	**NA**	**Sub‐Saharan Africa**	**0.672 (0.010–1.429)**	**1.146 (0.010–2.487)**	**70% (−66–765%)**	**49.342 (27.879–70.804)**	**7239% (2095–24436%)**	**NA**
**Central Sub‐Saharan Africa**	**Central Sub‐Saharan Africa**	**Sub‐Saharan Africa**	**0.020 (0.010–0.157)**	**0.041 (0.010–0.297)**	**102% (−100–2149756%)**	**4.239 (0.010–9.509)**	**20988% (−79–21356261%)**	**NA**
Angola	Central Sub‐Saharan Africa	Sub‐Saharan Africa	0.009 (0.010–0.066)	0.019 (0.010–0.120)	106% (**−**100–683439%)	1.892 (0.010–4.134)	20355% (**−**60–10590321%)	low
Central African Republic	Central Sub‐Saharan Africa	Sub‐Saharan Africa	0.000 (0.010–0.003)	0.000 (0.010–0.003)	20% (**−**100–49762084%)	0.047 (0.010–0.203)	15857% (**−**100–859112213%)	low
Congo	Central Sub‐Saharan Africa	Sub‐Saharan Africa	0.001 (0.010–0.010)	0.002 (0.010–0.015)	41% (**−**100–891133%)	0.197 (0.010–0.460)	13700% (**−**69–6104575%)	low
Democratic Republic of the Congo	Central Sub‐Saharan Africa	Sub‐Saharan Africa	0.007 (0.010–0.131)	0.015 (0.010–0.250)	124% (**−**100–3525787995797%)	1.757 (0.010–6.514)	25311% (**−**100–1916692220804%)	low
Equatorial Guinea	Central Sub‐Saharan Africa	Sub‐Saharan Africa	0.001 (0.010–0.005)	0.002 (0.010–0.010)	100% (**−**99–55552%)	0.188 (0.060–0.316)	18058% (204–1085338%)	low
Gabon	Central Sub‐Saharan Africa	Sub‐Saharan Africa	0.001 (0.010–0.006)	0.002 (0.010–0.009)	46% (**−**100–60899%)	0.158 (0.042–0.273)	13277% (53–1170181%)	low
**Eastern Sub‐Saharan Africa**	**Eastern Sub‐Saharan Africa**	**Sub‐Saharan Africa**	**0.073 (0.010–0.354)**	**0.155 (0.010–0.611)**	**113% (−98–27731%)**	**16.836 (4.929–28.744)**	**23074% (351–1191707%)**	**NA**
Burundi	Eastern Sub‐Saharan Africa	Sub‐Saharan Africa	0.001 (0.010–0.016)	0.002 (0.010–0.044)	187% (**−**100–201234740261096%)	0.233 (0.010–0.860)	30607% (**−**100–10853696049198%)	low
Comoros	Eastern Sub‐Saharan Africa	Sub‐Saharan Africa	0.000 (0.010–0.002)	0.000 (0.010–0.002)	67% (**−**100–123785332%)	0.022 (0.010–0.059)	16902% (**−**100–1061595699%)	low
Djibouti	Eastern Sub‐Saharan Africa	Sub‐Saharan Africa	0.000 (0.010–0.003)	0.001 (0.010–0.004)	50% (**−**100–678500%)	0.056 (0.010–0.123)	14341% (**−**63–5681781%)	low
Eritrea	Eastern Sub‐Saharan Africa	Sub‐Saharan Africa	0.001 (0.010–0.007)	0.001 (0.010–0.014)	83% (**−**100–69341565%)	0.139 (0.010–0.442)	19462% (**−**98–155557967%)	low
Ethiopia	Eastern Sub‐Saharan Africa	Sub‐Saharan Africa	0.022 (0.010–0.235)	0.048 (0.010–0.412)	120% (**−**100–60550075%)	5.613 (0.010–15.480)	25906% (**−**99–604399655%)	low
Kenya	Eastern Sub‐Saharan Africa	Sub‐Saharan Africa	0.014 (0.010–0.119)	0.025 (0.010–0.173)	82% (**−**100–2378841%)	2.857 (0.010–7.008)	20298% (**−**90–41894985%)	low
Madagascar	Eastern Sub‐Saharan Africa	Sub‐Saharan Africa	0.002 (0.010–0.022)	0.005 (0.010–0.051)	165% (**−**100–249273086%)	0.565 (0.010–2.283)	29430% (**−**99–1338756457%)	low
Malawi	Eastern Sub‐Saharan Africa	Sub‐Saharan Africa	0.002 (0.010–0.040)	0.004 (0.010–0.041)	107% (**−**100–493174892720%)	0.453 (0.010–1.491)	22991% (**−**100–8650297777421%)	low
Mozambique	Eastern Sub‐Saharan Africa	Sub‐Saharan Africa	0.002 (0.010–0.023)	0.006 (0.010–0.073)	143% (**−**100–793888672%)	0.602 (0.010–2.332)	25795% (**−**98–329608818%)	low
Rwanda	Eastern Sub‐Saharan Africa	Sub‐Saharan Africa	0.001 (0.010–0.004)	0.002 (0.010–0.008)	105% (**−**100–87394%)	0.128 (0.021–0.234)	16835% (99–1439908%)	low
Somalia	Eastern Sub‐Saharan Africa	Sub‐Saharan Africa	0.001 (0.010–0.009)	0.002 (0.010–0.014)	83% (**−**100–21550011%)	0.214 (0.010–0.874)	23417% (**−**98–352175644%)	low
South Sudan	Eastern Sub‐Saharan Africa	Sub‐Saharan Africa	0.001 (0.010–0.007)	0.003 (0.010–0.034)	244% (**−**100–200076243%)	0.304 (0.010–1.174)	37480% (**−**84–89618559%)	low
Uganda	Eastern Sub‐Saharan Africa	Sub‐Saharan Africa	0.007 (0.010–0.120)	0.015 (0.010–0.148)	128% (**−**100–49125889457%)	1.598 (0.010–4.561)	23998% (**−**100–672797060205%)	low
United Republic of Tanzania	Eastern Sub‐Saharan Africa	Sub‐Saharan Africa	0.016 (0.010–0.096)	0.034 (0.010–0.182)	111% (**−**100–170071%)	3.209 (0.381–6.037)	19987% (22–3303653%)	low
Zambia	Eastern Sub‐Saharan Africa	Sub‐Saharan Africa	0.004 (0.010–0.031)	0.008 (0.010–0.061)	108% (−100–4064723%)	0.842 (0.010–2.150)	22128% (**−**86–34456713%)	low
**Southern Sub‐Saharan Africa**	**Southern Sub‐Saharan Africa**	**Sub‐Saharan Africa**	**0.471 (0.010–1.057)**	**0.705 (0.010–1.560)**	**50% (−74–752%)**	**3.936 (1.306–6.566)**	**736% (103–3340%)**	**NA**
Botswana	Southern Sub‐Saharan Africa	Sub‐Saharan Africa	0.025 (0.010–0.057)	0.036 (0.010–0.079)	43% (**−**74–703%)	0.223 (0.129–0.317)	782% (135–3205%)	mid
Eswatini	Southern Sub‐Saharan Africa	Sub‐Saharan Africa	0.006 (0.010–0.017)	0.011 (0.010–0.028)	66% (**−**83–1516%)	0.057 (0.002–0.112)	781% (35–5650%)	mid
Lesotho	Southern Sub‐Saharan Africa	Sub‐Saharan Africa	0.004 (0.010–0.018)	0.006 (0.010–0.023)	50% (**−**98–9084%)	0.033 (0.010–0.102)	686% (**−**83–35543%)	mid
Namibia	Southern Sub‐Saharan Africa	Sub‐Saharan Africa	0.001 (0.010–0.005)	0.001 (0.010–0.008)	68% (**−**100–255372%)	0.127 (0.004–0.250)	15353% (**−**33–3582505%)	low
South Africa	Southern Sub‐Saharan Africa	Sub‐Saharan Africa	0.403 (0.010–0.980)	0.591 (0.010–1.428)	47% (**−**80–996%)	3.179 (0.670–5.688)	688% (54–3935%)	mid
Zimbabwe	Southern Sub‐Saharan Africa	Sub‐Saharan Africa	0.031 (0.010–0.132)	0.059 (0.010–0.227)	94% (**−**98–15204%)	0.317 (0.010–1.086)	937% (**−**83–63989%)	mid
**Western Sub‐Saharan Africa**	**Western Sub‐Saharan Africa**	**Sub‐Saharan Africa**	**0.109 (0.010–0.471)**	**0.245 (0.010–1.138)**	**125% (−98–30926%)**	**24.330 (7.473–41.187)**	**22220% (650–663815%)**	**NA**
Benin	Western Sub‐Saharan Africa	Sub‐Saharan Africa	0.003 (0.010–0.028)	0.007 (0.010–0.057)	120% (**−**100–19412588%)	0.666 (0.010–1.724)	22279% (**−**95–108286957%)	low
Burkina Faso	Western Sub‐Saharan Africa	Sub‐Saharan Africa	0.005 (0.010–0.043)	0.012 (0.010–0.121)	162% (**−**100–51712883%)	1.312 (0.010–3.687)	28276% (**−**94–128407764%)	low
Cabo Verde	Western Sub‐Saharan Africa	Sub‐Saharan Africa	0.000 (0.010–0.002)	0.000 (0.010–0.003)	32% (**−**100–570036%)	0.043 (0.010–0.098)	13842% (**−**76–8075037%)	low
Cameroon	Western Sub‐Saharan Africa	Sub‐Saharan Africa	0.009 (0.010–0.068)	0.016 (0.010–0.142)	76% (**−**100–4529151%)	1.747 (0.010–4.405)	19015% (**−**74–14274863%)	low
Chad	Western Sub‐Saharan Africa	Sub‐Saharan Africa	0.002 (0.010–0.022)	0.008 (0.010–0.078)	229% (**−**100–79776729%)	0.870 (0.010–2.713)	37146% (**−**94–220244872%)	low
Côte d'Ivoire	Western Sub‐Saharan Africa	Sub‐Saharan Africa	0.009 (0.010–0.062)	0.019 (0.010–0.136)	110% (**−**100–1120847%)	1.858 (0.010–4.207)	20666% (**−**50–8661263%)	low
Gambia	Western Sub‐Saharan Africa	Sub‐Saharan Africa	0.001 (0.010–0.007)	0.001 (0.010–0.012)	87% (**−**100–217540334%)	0.129 (0.010–0.352)	21407% (**−**99–575450391%)	low
Ghana	Western Sub‐Saharan Africa	Sub‐Saharan Africa	0.015 (0.010–0.099)	0.025 (0.010–0.183)	70% (**−**100–888627%)	2.456 (0.010–5.521)	16674% (**−**53–5968674%)	low
Guinea	Western Sub‐Saharan Africa	Sub‐Saharan Africa	0.003 (0.010–0.025)	0.006 (0.010–0.056)	110% (**−**100–21069537%)	0.668 (0.010–1.843)	23511% (**−**92–72450406%)	low
Guinea‐Bissau	Western Sub‐Saharan Africa	Sub‐Saharan Africa	0.000 (0.010–0.004)	0.001 (0.010–0.008)	106% (**−**100–3453836794%)	0.073 (0.010–0.213)	22332% (**−**100–7083922923%)	low
Liberia	Western Sub‐Saharan Africa	Sub‐Saharan Africa	0.001 (0.010–0.007)	0.002 (0.010–0.016)	104% (**−**100–43980254%)	0.199 (0.010–0.632)	25350% (**−**95–119314991%)	low
Mali	Western Sub‐Saharan Africa	Sub‐Saharan Africa	0.005 (0.010–0.040)	0.012 (0.010–0.115)	160% (**−**100–34686880%)	1.318 (0.010–3.872)	29060% (**−**92–100393474%)	low
Mauritania	Western Sub‐Saharan Africa	Sub‐Saharan Africa	0.001 (0.010–0.011)	0.003 (0.010–0.022)	97% (**−**100–3545789%)	0.271 (0.010–0.637)	19678% (**−**79–18563345%)	low
Niger	Western Sub‐Saharan Africa	Sub‐Saharan Africa	0.002 (0.010–0.037)	0.006 (0.010–0.073)	204% (**−**100–603907003356%)	0.670 (0.010–2.868)	34411% (**−**100–3444007058581%)	low
Nigeria	Western Sub‐Saharan Africa	Sub‐Saharan Africa	0.046 (0.010–0.378)	0.114 (0.010–0.950)	148% (**−**100–7162847%)	10.413 (0.010–25.775)	22489% (**−**86–35525875%)	low
Sao Tome and Principe	Western Sub‐Saharan Africa	Sub‐Saharan Africa	0.000 (0.010–0.000)	0.000 (0.010–0.001)	54% (**−**100–3699369%)	0.010 (0.010–0.025)	15856% (**−**81–13352488%)	low
Senegal	Western Sub‐Saharan Africa	Sub‐Saharan Africa	0.004 (0.010–0.034)	0.008 (0.010–0.058)	97% (**−**100–3377553%)	0.908 (0.010–2.303)	22570% (**−**88–42633337%)	low

## Appendix 2 Per‐Capita Periodontitis Expenditure (2025 US$) in 2021, With Projections to 2050, Across 21 Regions and 204 Countries

**Table:**

Country/region	Super‐region	Region	2021 per capita expenditure (US$ [95% UI])	2050 per capita expenditure (current‐usage scenario; US$ [95% UI])	Per capita % change 2021–2050 (current‐usage scenario; % [95% UI])	2050 per capita expenditure (expanded coverage scenario; US$ [95% UI])	Per capita % change 2021–2050 (expanded coverage scenario; % [95% UI])	Dental utilisation scenario
**Global**	**NA**	**NA**	**21.32 (16.97–25.67)**	**18.36 (14.43–22.29)**	**–14% (−36–16%)**	**52.56 (40.86–64.26)**	**147% (82–233%)**	**NA**
**Central Europe, Eastern Europe, and Central Asia**	**NA**	**Central Europe, Eastern Europe, and Central Asia**	**4.71 (1.60–7.81)**	**4.34 (1.53–7.15)**	**–8% (−63–133%)**	**74.30 (54.35–94.25)**	**1479% (674–3121%)**	**NA**
**Central Asia**	**Central Asia**	**Central Europe, Eastern Europe, and Central Asia**	**1.51 (0.00–3.03)**	**1.54 (0.00–3.11)**	**2% (−76–330%)**	**50.43 (32.83–68.03)**	**3248% (1047–9671%)**	**NA**
Armenia	Central Asia	Central Europe, Eastern Europe, and Central Asia	9.86 (0.00–24.04)	10.59 (0.00–26.94)	7% (−87–785%)	52.16 (8.17–96.14)	429% (0–2698%)	Mid
Azerbaijan	Central Asia	Central Europe, Eastern Europe, and Central Asia	0.67 (0.00–3.25)	0.73 (0.00–3.75)	10% (−100–31 807%)	64.09 (16.94–111.23)	9534% (86–499 718%)	Low
Georgia	Central Asia	Central Europe, Eastern Europe, and Central Asia	0.59 (0.00–3.41)	0.61 (0.00–3.25)	3% (−100–67 538%)	53.87 (11.06–96.68)	9070% (−30–1 204 567%)	Low
Kazakhstan	Central Asia	Central Europe, Eastern Europe, and Central Asia	0.92 (0.00–4.73)	0.89 (0.00–4.28)	−3% (−100–27 075%)	79.30 (34.01–124.60)	8547% (30–573 391%)	Low
Kyrgyzstan	Central Asia	Central Europe, Eastern Europe, and Central Asia	0.23 (0.00–1.83)	0.23 (0.00–1.55)	0% (−100–862 900%)	24.39 (0.00–58.48)	10 556% (−91–13 129 350%)	Low
Mongolia	Central Asia	Central Europe, Eastern Europe, and Central Asia	0.53 (0.00–2.83)	0.52 (0.00–2.99)	−1% (−100–61 162%)	46.93 (6.74–87.11)	8810% (4–762 385%)	Low
Tajikistan	Central Asia	Central Europe, Eastern Europe, and Central Asia	0.23 (0.00–1.99)	0.22 (0.00–1.74)	−3% (−100–3 052 655%)	22.19 (0.00–54.31)	9644% (−96–25 752 460%)	Low
Turkmenistan	Central Asia	Central Europe, Eastern Europe, and Central Asia	13.02 (0.00–29.74)	13.91 (0.00–32.41)	7% (−83–579%)	72.09 (25.34–118.84)	454% (31–2235%)	Mid
Uzbekistan	Central Asia	Central Europe, Eastern Europe, and Central Asia	0.45 (0.00–2.63)	0.47 (0.00–2.66)	5% (−100–82 959%)	43.24 (5.98–80.50)	9477% (−28–1 275 384%)	Low
**Central Europe**	**Central Europe**	**Central Europe, Eastern Europe, and Central Asia**	**13.19 (3.10–23.28)**	**13.43 (3.08–23.79)**	**2% (−66–202%)**	**90.58 (67.11–114.06)**	**587% (206–1441%)**	**NA**
Albania	Central Europe	Central Europe, Eastern Europe, and Central Asia	0.58 (0.00–3.07)	0.59 (0.00–3.11)	3% (−100–44 176%)	54.94 (10.70–99.19)	9449% (16–784 754%)	Low
Bosnia and Herzegovina	Central Europe	Central Europe, Eastern Europe, and Central Asia	11.07 (0.00–27.51)	11.58 (0.00–28.84)	5% (−87–758%)	56.28 (11.13–101.42)	408% (−6–2651%)	Mid
Bulgaria	Central Europe	Central Europe, Eastern Europe, and Central Asia	0.79 (0.00–3.86)	0.80 (0.00–3.81)	0% (−100–22 264%)	69.63 (26.48–112.77)	8668% (76–437 023%)	Low
Croatia	Central Europe	Central Europe, Eastern Europe, and Central Asia	20.86 (0.00–49.38)	22.00 (0.00–53.66)	5% (−86–668%)	102.72 (38.46–166.98)	392% (9–2115%)	Mid
Czechia	Central Europe	Central Europe, Eastern Europe, and Central Asia	22.02 (0.00–50.27)	21.44 (0.00–48.86)	−3% (−84–496%)	106.08 (49.56–162.59)	382% (20–1833%)	Mid
Hungary	Central Europe	Central Europe, Eastern Europe, and Central Asia	13.97 (0.00–31.06)	13.97 (0.00–31.13)	0% (−82–466%)	76.19 (35.70–116.68)	445% (44–1968%)	Mid
Montenegro	Central Europe	Central Europe, Eastern Europe, and Central Asia	0.75 (0.00–3.67)	0.75 (0.00–3.78)	0% (−100–27 369%)	67.98 (21.75–114.20)	8958% (74–470 231%)	Low
North Macedonia	Central Europe	Central Europe, Eastern Europe, and Central Asia	0.63 (0.00–3.42)	0.67 (0.00–3.66)	7% (−100–56 939%)	59.23 (13.35–105.11)	9315% (4–855 185%)	Low
Poland	Central Europe	Central Europe, Eastern Europe, and Central Asia	21.21 (0.00–49.24)	21.48 (0.00–50.20)	1% (−85–564%)	103.52 (44.17–162.87)	388% (16–1960%)	Mid
Romania	Central Europe	Central Europe, Eastern Europe, and Central Asia	1.00 (0.00–4.72)	1.01 (0.00–4.82)	1% (−100–20 634%)	88.40 (35.27–141.53)	8776% (101–391 903%)	Low
Serbia	Central Europe	Central Europe, Eastern Europe, and Central Asia	0.70 (0.00–3.42)	0.76 (0.00–3.80)	8% (−100–28 373%)	69.04 (20.00–118.08)	9756% (91–509 440%)	Low
Slovakia	Central Europe	Central Europe, Eastern Europe, and Central Asia	19.75 (0.00–45.45)	19.81 (0.00–45.82)	0% (−84–537%)	97.62 (44.04–151.21)	394% (20–1929%)	Mid
Slovenia	Central Europe	Central Europe, Eastern Europe, and Central Asia	25.62 (0.00–59.93)	24.04 (0.00–56.14)	−6% (−86–522%)	115.60 (51.33–179.87)	351% (6–1823%)	Mid
Eastern Europe	Eastern Europe	Central Europe, Eastern Europe, and Central Asia	1.53 (0.00–4.33)	1.48 (0.00–4.26)	−3% (−93–1242%)	81.72 (41.55–121.89)	5247% (700–35 648%)	NA
Belarus	Eastern Europe	Central Europe, Eastern Europe, and Central Asia	0.87 (0.00–4.27)	0.85 (0.00–4.16)	−3% (−100–23 867%)	75.90 (25.04–126.75)	8588% (69–446 541%)	Low
Estonia	Eastern Europe	Central Europe, Eastern Europe, and Central Asia	22.88 (0.00–52.11)	23.33 (0.00–52.70)	2% (−83–513%)	113.22 (51.01–175.44)	395% (23–1888%)	Mid
Latvia	Eastern Europe	Central Europe, Eastern Europe, and Central Asia	20.89 (0.00–48.78)	21.22 (0.00–50.21)	2% (−85–586%)	102.42 (41.62–163.22)	390% (14–2013%)	Mid
Lithuania	Eastern Europe	Central Europe, Eastern Europe, and Central Asia	24.00 (0.00–55.07)	25.03 (0.00–58.01)	4% (−84–561%)	119.76 (52.65–186.88)	399% (22–1945%)	Mid
Republic of Moldova	Eastern Europe	Central Europe, Eastern Europe, and Central Asia	0.49 (0.00–2.93)	0.51 (0.00–3.07)	5% (−100–120 079%)	49.73 (1.38–98.08)	10 064% (−37–1 632 739%)	Low
Russian Federation	Eastern Europe	Central Europe, Eastern Europe, and Central Asia	1.03 (0.00–4.90)	1.00 (0.00–4.68)	−3% (−99–18 446%)	89.46 (35.90–143.02)	8580% (95–387 220%)	Low
Ukraine	Eastern Europe	Central Europe, Eastern Europe, and Central Asia	0.52 (0.00–2.87)	0.54 (0.00–3.19)	4% (−100–85 800%)	50.40 (4.50–96.29)	9665% (−6–1 017 358%)	Low
**High‐income**	**NA**	**High‐income**	**118.56 (89.97–147.15)**	**121.71 (90.97–152.44)**	**3% (−28–46%)**	**131.56 (100.71–162.41)**	**11% (−21–55%)**	**NA**
Australasia	**Australasia**	**High‐income**	**148.17 (88.05–208.29)**	**150.92 (89.09–212.74)**	**2% (−43–81%)**	**164.92 (102.53–227.30)**	**11% (−36–94%)**	**NA**
Australia	Australasia	High‐income	172.95 (101.06–244.85)	172.35 (100.23–244.48)	0% (−45–80%)	172.35 (100.23–244.48)	0% (−45–80%)	High
New Zealand	Australasia	High‐income	24.52 (0.00–55.64)	24.71 (0.00–56.49)	1% (−83–514%)	121.13 (55.41–186.84)	394% (24–1865%)	Mid
**High‐income Asia Pacific**	**High‐income Asia Pacific**	**High‐income**	**57.63 (37.78–77.48)**	**56.58 (37.90–75.26)**	**−2% (−39–58%)**	**65.87 (46.77–84.98)**	**14% (−27–79%)**	**NA**
Brunei Darussalam	High‐income Asia Pacific	High‐income	1.92 (0.00–8.93)	2.12 (0.00–9.43)	10% (−99–16 478%)	181.98 (100.46–263.51)	9354% (142–368 866%)	Low
Japan	High‐income Asia Pacific	High‐income	65.52 (37.96–93.08)	64.61 (37.76–91.46)	−1% (−45–78%)	64.61 (37.76–91.46)	−1% (−45–78%)	High
Republic of Korea	High‐income Asia Pacific	High‐income	40.17 (20.04–60.30)	42.50 (20.07–64.93)	6% (−49–119%)	42.50 (20.07–64.93)	6% (−49–119%)	High
Singapore	High‐income Asia Pacific	High‐income	43.47 (0.00–95.20)	44.64 (0.00–98.89)	3% (−81–463%)	223.04 (124.57–321.52)	413% (44–1725%)	Mid
**High‐income North America**	**High‐income North America**	**High‐income**	**191.99 (111.50–272.49)**	**192.52 (110.37–274.67)**	**0% (−45–82%)**	**192.55 (110.40–274.70)**	**0% (−45–82%)**	**NA**
Canada	High‐income North America	High‐income	139.61 (66.80–212.43)	138.75 (66.62–210.88)	−1% (−52–108%)	138.75 (66.62–210.88)	−1% (−52–108%)	High
Greenland	High‐income North America	High‐income	2.40 (0.00–10.64)	2.50 (0.00–10.82)	4% (−99–12 271%)	204.09 (112.61–295.57)	8396% (168–269 694%)	Low
United States of America	High‐income North America	High‐income	197.92 (108.72–287.12)	199.00 (107.40–290.61)	1% (−47–91%)	199.00 (107.40–290.61)	1% (−47–91%)	High
**Southern Latin America**	**Southern Latin America**	**High‐income**	**10.70 (0.00–23.36)**	**12.43 (0.00–28.74)**	**16% (−80–581%)**	**92.23 (52.54–131.91)**	**762% (145–2940%)**	**NA**
Argentina	Southern Latin America	High‐income	13.89 (0.00–32.52)	15.98 (0.00–39.34)	15% (−84–736%)	76.54 (23.49–129.59)	451% (22–2392%)	Mid
Chile	Southern Latin America	High‐income	1.46 (0.00–6.45)	1.58 (0.00–7.17)	8% (−99–14 555%)	133.11 (77.10–189.13)	9007% (193–283 082%)	Low
Uruguay	Southern Latin America	High‐income	18.95 (0.00–46.10)	20.38 (0.00–50.54)	8% (−86–744%)	96.62 (29.44–163.81)	410% (4–2407%)	Mid
**Western Europe**	**Western Europe**	**High‐income**	**96.82 (78.02–115.63)**	**97.04 (78.77–115.31)**	**0% (−24–31%)**	**103.48 (85.13–121.83)**	**7% (−18–39%)**	**NA**
Andorra	Western Europe	High‐income	116.88 (55.20–178.56)	113.54 (52.05–175.04)	−3% (−54–107%)	113.54 (52.05–175.04)	−3% (−54–107%)	High
Austria	Western Europe	High‐income	113.13 (55.93–170.33)	111.29 (54.60–167.98)	−2% (−52–102%)	111.29 (54.60–167.98)	−2% (−52–102%)	High
Belgium	Western Europe	High‐income	123.48 (54.44–192.51)	121.19 (54.84–187.54)	−2% (−55–115%)	121.19 (54.84–187.54)	−2% (−55–115%)	High
Cyprus	Western Europe	High‐income	1.14 (0.00–5.18)	1.16 (0.00–5.22)	2% (−99–14 981%)	101.43 (47.81–155.05)	8829% (144–326 689%)	Low
Denmark	Western Europe	High‐income	152.64 (62.13–243.15)	149.57 (61.71–237.42)	−2% (−57–126%)	149.57 (61.71–237.42)	−2% (−57–126%)	High
Finland	Western Europe	High‐income	28.56 (0.00–66.11)	28.60 (0.00–66.39)	0% (−84–546%)	137.32 (65.62–209.01)	381% (17–1880%)	Mid
France	Western Europe	High‐income	87.20 (48.36–126.03)	86.44 (48.17–124.71)	−1% (−47–86%)	86.44 (48.17–124.71)	−1% (−47–86%)	High
Germany	Western Europe	High‐income	158.03 (86.24–229.82)	155.52 (84.84–226.21)	−2% (−48–87%)	155.52 (84.84–226.21)	−2% (−48–87%)	High
Greece	Western Europe	High‐income	16.88 (0.00–38.97)	16.62 (0.00–38.77)	−2% (−85–538%)	83.20 (33.76–132.64)	393% (17–1976%)	Mid
Iceland	Western Europe	High‐income	113.36 (55.91–170.81)	112.80 (55.06–170.54)	0% (−52–104%)	112.80 (55.06–170.54)	0% (−52–104%)	High
Ireland	Western Europe	High‐income	32.01 (0.00–68.94)	32.47 (0.00–69.61)	1% (−80–415%)	176.63 (97.39–255.87)	452% (60–1802%)	Mid
Israel	Western Europe	High‐income	94.91 (44.63–145.19)	94.76 (44.60–144.91)	0% (−53–111%)	94.76 (44.60–144.91)	0% (−53–111%)	High
Italy	Western Europe	High‐income	74.02 (21.13–126.92)	72.83 (20.79–124.86)	−2% (−64–170%)	72.83 (20.79–124.86)	−2% (−64–170%)	High
Luxembourg	Western Europe	High‐income	195.22 (101.28–289.16)	191.39 (101.05–281.72)	−2% (−50–92%)	191.39 (101.05–281.72)	−2% (−50–92%)	High
Malta	Western Europe	High‐income	23.83 (0.00–53.10)	23.18 (0.00–51.42)	−3% (−83–449%)	117.95 (59.39–176.51)	395% (32–1762%)	Mid
Monaco	Western Europe	High‐income	5.84 (0.00–26.62)	NA	NA	NA	NA	Low
Netherlands	Western Europe	High‐income	117.49 (58.43–176.54)	116.35 (59.51–173.18)	−1% (−51–100%)	116.35 (59.51–173.18)	−1% (−51–100%)	High
Norway	Western Europe	High‐income	171.77 (81.55–261.99)	169.89 (80.34–259.44)	−1% (−53–108%)	169.89 (80.34–259.44)	−1% (−53–108%)	High
Portugal	Western Europe	High‐income	18.18 (0.00–41.17)	18.01 (0.00–41.08)	−1% (−84–499%)	90.39 (39.72–141.05)	397% (25–1884%)	Mid
San Marino	Western Europe	High‐income	131.48 (63.75–199.20)	NA	NA	NA	NA	High
Spain	Western Europe	High‐income	54.35 (29.65–79.06)	53.25 (29.41–77.09)	−2% (−48–85%)	53.25 (29.41–77.09)	−2% (−48–85%)	High
Sweden	Western Europe	High‐income	238.06 (136.13–339.99)	237.13 (138.68–335.57)	0% (−45–81%)	237.13 (138.68–335.57)	0% (−45–81%)	High
Switzerland	Western Europe	High‐income	172.93 (95.52–250.35)	170.79 (95.91–245.68)	−1% (−47–85%)	170.79 (95.91–245.68)	–1% (−47–85%)	High
United Kingdom	Western Europe	High‐income	59.77 (19.11–100.42)	60.66 (18.73–102.60)	1% (−62–168%)	60.66 (18.73–102.60)	1% (−62–168%)	High
**Latin America and Caribbean**	**NA**	**Latin America and Caribbean**	**5.45 (0.88–10.01)**	**5.05 (1.07–9.04)**	**−7% (−71–193%)**	**61.14 (44.32–77.96)**	**1022% (365–2609%)**	**NA**
**Andean Latin America**	**Andean Latin America**	**Latin America and Caribbean**	**0.49 (0.00–1.94)**	**0.51 (0.00–2.00)**	**3% (−98–6517%)**	**47.37 (20.76–73.97)**	**9522% (380–192 955%)**	**NA**
Bolivia (Plurinational State of)	Andean Latin America	Latin America and Caribbean	0.35 (0.00–2.26)	0.38 (0.00–2.65)	9% (−100–356 979%)	36.46 (0.00–75.75)	10 347% (−61–2 775 981%)	Low
Ecuador	Andean Latin America	Latin America and Caribbean	0.51 (0.00–3.31)	0.52 (0.00–2.99)	3% (−100–151 803%)	47.86 (3.48–92.24)	9372% (−66–2 657 665%)	Low
Peru	Andean Latin America	Latin America and Caribbean	0.53 (0.00–2.69)	0.55 (0.00–2.89)	3% (−100–36 487%)	51.30 (9.58–93.02)	9536% (55–598 914%)	Low
**Caribbean**	**Caribbean**	**Latin America and Caribbean**	**4.79 (0.00–9.67)**	**4.45 (0.22–8.68)**	**−7% (−77–274%)**	**54.44 (32.51–76.37)**	**1036% (280–3297%)**	**NA**
Antigua and Barbuda	Caribbean	Latin America and Caribbean	18.04 (0.00–43.52)	19.78 (0.00–48.85)	10% (−86–742%)	93.32 (28.49–158.15)	417% (7–2396%)	Mid
Bahamas	Caribbean	Latin America and Caribbean	21.97 (0.00–51.43)	24.58 (0.00–59.29)	12% (−84–684%)	114.51 (44.52–184.50)	421% (19–2174%)	Mid
Barbados	Caribbean	Latin America and Caribbean	14.12 (0.00–36.59)	14.99 (0.00–38.98)	6% (−89–914%)	70.56 (10.90–130.22)	400% (−18–2927%)	Mid
Belize	Caribbean	Latin America and Caribbean	8.39 (0.00–22.31)	9.91 (0.00–26.86)	18% (−89–1181%)	48.68 (0.36–97.01)	480% (−16–3912%)	Mid
Bermuda	Caribbean	Latin America and Caribbean	2.70 (0.00–12.61)	2.61 (0.00–11.82)	−3% (−99–15 738%)	220.51 (108.74–332.28)	8075% (100–334 403%)	Low
Cuba	Caribbean	Latin America and Caribbean	10.40 (0.00–29.12)	10.59 (0.00–29.99)	2% (−92–1226%)	50.10 (0.00–102.71)	382% (−40–3766%)	Mid
Dominica	Caribbean	Latin America and Caribbean	10.21 (0.00–26.88)	11.77 (0.00–31.61)	15% (−89–1104%)	56.45 (0.92–111.98)	453% (−18–3617%)	Mid
Dominican Republic	Caribbean	Latin America and Caribbean	0.79 (0.00–3.82)	0.83 (0.00–4.04)	5% (−100–25 168%)	74.72 (21.45–127.99)	9405% (87–483 974%)	Low
Grenada	Caribbean	Latin America and Caribbean	12.40 (0.00–30.84)	13.98 (0.00–36.15)	13% (−87–891%)	66.82 (8.70–124.95)	439% (−4–2920%)	Mid
Guyana	Caribbean	Latin America and Caribbean	0.87 (0.00–4.39)	0.94 (0.00–4.52)	8% (−100–28 180%)	81.94 (28.74–135.14)	9339% (54–577 542%)	Low
Haiti	Caribbean	Latin America and Caribbean	0.17 (0.00–1.62)	0.18 (0.00–1.60)	6% (−100–15 621 507%)	19.68 (0.00–56.62)	11 755% (−98–91 106 096%)	Low
Jamaica	Caribbean	Latin America and Caribbean	8.89 (0.00–23.88)	11.00 (0.00–30.93)	24% (−90–1372%)	51.95 (0.00–106.80)	484% (−20–4175%)	Mid
Puerto Rico	Caribbean	Latin America and Caribbean	1.41 (0.00–6.61)	1.44 (0.00–7.20)	2% (−100–23 346%)	128.82 (48.57–209.08)	9020% (119–380 511%)	Low
Saint Kitts and Nevis	Caribbean	Latin America and Caribbean	20.31 (0.00–48.87)	NA	NA	NA	NA	Mid
Saint Lucia	Caribbean	Latin America and Caribbean	12.37 (0.00–32.43)	13.40 (0.00–35.57)	8% (−89–998%)	63.55 (6.17–120.93)	414% (−20–3184%)	Mid
Saint Vincent and the Grenadines	Caribbean	Latin America and Caribbean	12.23 (0.00–31.17)	13.44 (0.00–34.69)	10% (−88–906%)	64.61 (8.72–120.50)	429% (−10–3018%)	Mid
Suriname	Caribbean	Latin America and Caribbean	11.76 (0.00–30.04)	12.41 (0.00–31.70)	6% (−88–851%)	60.41 (8.53–112.30)	414% (−13–2937%)	Mid
Trinidad and Tobago	Caribbean	Latin America and Caribbean	21.25 (0.00–50.48)	22.74 (0.00–55.59)	7% (−85–687%)	106.20 (35.58–176.82)	400% (8–2204%)	Mid
United States Virgin Islands	Caribbean	Latin America and Caribbean	1.46 (0.00–6.96)	1.43 (0.00–6.94)	−2% (−100–21 649%)	124.03 (48.53–199.52)	8422% (85–392 631%)	Low
**Central Latin America**	**Central Latin America**	**Latin America and Caribbean**	**2.28 (0.00–4.70)**	**2.63 (0.00–5.46)**	**16% (−75–424%)**	**64.61 (33.40–95.83)**	**2739% (783–9030%)**	**NA**
Colombia	Central Latin America	Latin America and Caribbean	0.70 (0.00–3.60)	0.77 (0.00–4.25)	11% (−100–50 901%)	69.91 (12.00–127.83)	9932% (43–703 457%)	Low
Costa Rica	Central Latin America	Latin America and Caribbean	14.93 (0.00–36.58)	16.76 (0.00–42.84)	12% (−87–841%)	79.55 (18.36–140.73)	433% (3–2648%)	Mid
El Salvador	Central Latin America	Latin America and Caribbean	0.44 (0.00–2.61)	0.50 (0.00–3.09)	14% (−100–143 577%)	48.49 (0.08–96.90)	10 923% (−27–1 673 098%)	Low
Guatemala	Central Latin America	Latin America and Caribbean	7.66 (0.00–20.02)	9.41 (0.00–26.07)	23% (−89–1246%)	45.07 (0.00–90.97)	488% (−13–3861%)	Mid
Honduras	Central Latin America	Latin America and Caribbean	0.30 (0.00–2.70)	0.34 (0.00–2.42)	14% (−100–3 078 602%)	33.84 (0.00–77.18)	11 340% (−97–42 156 844%)	Low
Mexico	Central Latin America	Latin America and Caribbean	0.80 (0.00–4.01)	0.83 (0.00–4.31)	5% (−100–34 560%)	76.40 (20.12–132.68)	9477% (59–57 7235%)	Low
Nicaragua	Central Latin America	Latin America and Caribbean	0.31 (0.00–2.16)	0.38 (0.00–2.73)	22% (−100–662 609%)	36.87 (0.00–83.45)	11 759% (−73–5 193 648%)	Low
Panama	Central Latin America	Latin America and Caribbean	17.70 (0.00–41.72)	19.20 (0.00–45.41)	8% (−84–643%)	92.64 (33.63–151.66)	423% (17–2243%)	Mid
Venezuela (Bolivarian Republic of)	Central Latin America	Latin America and Caribbean	6.12 (0.00–19.22)	6.33 (0.00–20.23)	4% (−95–2122%)	30.36 (0.00–76.31)	396% (−64–6738%)	Mid
Tropical Latin America	**Tropical Latin America**	**Latin America and Caribbean**	**10.55 (0.00–22.08)**	**10.14 (0.00–21.08)**	**−4% (−79–346%)**	**62.81 (40.37–85.26)**	**495% (89–1779%)**	**NA**
Brazil	Tropical Latin America	Latin America and Caribbean	10.88 (0.00–22.78)	10.51 (0.00–21.89)	−3% (−79–350%)	63.09 (39.83–86.34)	480% (83–1740%)	Mid
Paraguay	Tropical Latin America	Latin America and Caribbean	0.53 (0.00–2.91)	0.60 (0.00–3.25)	14% (−100–62 188%)	55.87 (8.63–103.11)	10 496% (7–1 049 286%)	Low
**North Africa and Middle East**	**NA**	**North Africa and Middle East**	**4.87 (1.59–8.16)**	**4.36 (1.24–7.48)**	**−11% (−67–139%)**	**59.97 (45.66–74.28)**	**1131% (502–2417%)**	**NA**
**North Africa and Middle East**	**North Africa and Middle East**	**North Africa and Middle East**	**4.87 (1.59–8.16)**	**4.36 (1.24–7.48)**	**−11% (−67–139%)**	**59.97 (45.66–74.28)**	**1131% (502–2417%)**	**NA**
Afghanistan	North Africa and Middle East	North Africa and Middle East	0.09 (0.00–1.46)	0.10 (0.00–0.94)	7% (−100–3 360 762 346%)	11.69 (0.00–42.61)	12 683% (−100–49 232 627 996%)	Low
Algeria	North Africa and Middle East	North Africa and Middle East	0.59 (0.00–3.03)	0.63 (0.00–3.57)	8% (−100–54 278%)	56.92 (11.60–102.24)	9603% (40–673 130%)	Low
Bahrain	North Africa and Middle East	North Africa and Middle East	27.98 (0.00–63.35)	30.63 (0.00–70.16)	9% (−82–567%)	149.63 (73.76–225.50)	435% (37–1988%)	Mid
Egypt	North Africa and Middle East	North Africa and Middle East	0.55 (0.00–3.06)	0.55 (0.00–2.95)	0% (−100–56 428%)	50.60 (8.58–92.62)	9130% (−13–974 173%)	Low
Iran (Islamic Republic of)	North Africa and Middle East	North Africa and Middle East	13.57 (0.00–31.79)	15.56 (0.00–38.38)	15% (−84–738%)	75.20 (19.34–131.06)	454% (19–2473%)	Mid
Iraq	North Africa and Middle East	North Africa and Middle East	0.54 (0.00–3.07)	0.60 (0.00–3.47)	10% (−100–91 457%)	53.78 (9.62–97.94)	9845% (−15–1 156 769%)	Low
Jordan	North Africa and Middle East	North Africa and Middle East	0.46 (0.00–2.70)	0.50 (0.00–2.86)	8% (−100–100 225%)	45.95 (3.94–87.97)	9923% (−31–1 448 987%)	Low
Kuwait	North Africa and Middle East	North Africa and Middle East	33.59 (0.00–74.95)	36.30 (0.00–84.48)	8% (−82–561%)	171.80 (80.54–263.05)	411% (34–1855%)	Mid
Lebanon	North Africa and Middle East	North Africa and Middle East	9.00 (0.00–23.78)	10.39 (0.00–28.14)	15% (−89–1137%)	50.61 (1.41–99.80)	463% (−17–3697%)	Mid
Libya	North Africa and Middle East	North Africa and Middle East	0.69 (0.00–3.46)	0.79 (0.00–4.12)	15% (−100–39 103%)	71.35 (18.07–124.63)	10 300% (72–630 427%)	Low
Morocco	North Africa and Middle East	North Africa and Middle East	0.47 (0.00–2.77)	0.53 (0.00–3.20)	11% (−100–124 390%)	50.15 (0.00–100.84)	10 493% (−25–1 504 057%)	Low
Oman	North Africa and Middle East	North Africa and Middle East	1.33 (0.00–6.17)	1.47 (0.00–6.82)	11% (−99–19 352%)	125.48 (59.35–191.60)	9368% (135–381 271%)	Low
Palestine	North Africa and Middle East	North Africa and Middle East	0.26 (0.00–2.23)	0.31 (0.00–2.35)	16% (−100–2 603 240%)	28.62 (0.00–65.81)	10 751% (−94–21 255 048%)	Low
Qatar	North Africa and Middle East	North Africa and Middle East	3.69 (0.00–16.66)	3.91 (0.00–17.36)	6% (−99–14 405%)	326.00 (176.33–475.67)	8736% (155–306 303%)	Low
Saudi Arabia	North Africa and Middle East	North Africa and Middle East	24.19 (0.00–52.85)	28.16 (0.00–62.14)	16% (−79–532%)	141.32 (72.92–209.73)	484% (62–2001%)	Mid
Sudan	North Africa and Middle East	North Africa and Middle East	0.19 (0.00–1.81)	0.22 (0.00–1.98)	20% (−100–14 094 210%)	21.95 (0.00–57.51)	11 627% (−98–77 902 281%)	Low
Syrian Arab Republic	North Africa and Middle East	North Africa and Middle East	0.22 (0.00–2.29)	0.25 (0.00–2.14)	14% (−100–19 192 116%)	24.96 (0.00–63.16)	11 239% (−99–153 420 710%)	Low
Tunisia	North Africa and Middle East	North Africa and Middle East	0.52 (0.00–2.74)	0.58 (0.00–3.42)	11% (−100–75 801%)	53.75 (4.70–102.81)	10 269% (31–822 304%)	Low
Türkiye	North Africa and Middle East	North Africa and Middle East	1.08 (0.00–5.03)	1.11 (0.00–5.29)	4% (−99–19 651%)	98.34 (38.06–158.62)	9041% (120–379 505%)	Low
United Arab Emirates	North Africa and Middle East	North Africa and Middle East	46.35 (0.00–105.43)	43.58 (0.00–99.47)	−6% (−85–473%)	204.88 (105.92–303.83)	342% (13–1628%)	Mid
Yemen	North Africa and Middle East	North Africa and Middle East	0.13 (0.00–1.79)	0.14 (0.00–1.15)	2% (−100–177 804 771%)	16.82 (0.00–51.26)	12 417% (−100–3 226 639 520%)	Low
**South Asia**	**NA**	**South Asia**	**0.47 (0.00–2.70)**	**0.53 (0.00–2.83)**	**12% (−100–67 859%)**	**51.93 (4.93–98.93)**	**10 898% (−10–1 339 053%)**	**NA**
**South Asia**	**South Asia**	**South Asia**	**0.47 (0.00–2.70)**	**0.53 (0.00–2.83)**	**12% (−100–67 859%)**	**51.93 (4.93–98.93)**	**10 898% (−10–1 339 053%)**	**NA**
Bangladesh	South Asia	South Asia	0.39 (0.00–2.49)	0.51 (0.00–3.62)	29% (−100–430 809%)	50.86 (0.00–118.34)	12 833% (−47–3 143 267%)	Low
Bhutan	South Asia	South Asia	0.63 (0.00–3.56)	0.74 (0.00–4.41)	16% (−100–103 223%)	71.79 (0.14–143.45)	11 210% (1–1 270 821%)	Low
India	South Asia	South Asia	0.50 (0.00–3.36)	0.55 (0.00–3.51)	10% (−100–302 369%)	53.08 (0.00–113.49)	10 604% (−70–3 839 260%)	Low
Nepal	South Asia	South Asia	0.24 (0.00–2.56)	0.29 (0.00–2.48)	21% (−100–22 081 780%)	30.94 (0.00–78.90)	12 670% (−99–204 405 895%)	Low
Pakistan	South Asia	South Asia	0.41 (0.00–3.07)	0.49 (0.00–3.52)	18% (−100–878 282%)	48.76 (0.00–111.18)	11 665% (−83–8 157 643%)	Low
**Southeast Asia, East Asia, and Oceania**	**NA**	**Southeast Asia, East Asia, and Oceania**	**13.26 (7.24–19.28)**	**12.39 (6.41–18.38)**	**−7% (−52–81%)**	**31.16 (20.86–41.45)**	**135% (34–312%)**	**NA**
**East Asia**	**East Asia**	**Southeast Asia, East Asia, and Oceania**	**19.39 (10.49–28.30)**	**19.62 (10.00–29.24)**	**1% (−48–98%)**	**19.90 (10.26–29.54)**	**3% (−47–100%)**	**NA**
China	East Asia	Southeast Asia, East Asia, and Oceania	18.59 (9.41–27.77)	18.70 (8.79–28.62)	1% (−51–108%)	18.70 (8.79–28.62)	1% (−51–108%)	High
Democratic People's Republic of Korea	East Asia	Southeast Asia, East Asia, and Oceania	0.12 (0.00–1.67)	0.13 (0.00–1.34)	12% (−100–838 362 489%)	15.84 (0.00–49.73)	13 183% (−100–6 968 092 595%)	Low
Taiwan (Province of China)	East Asia	Southeast Asia, East Asia, and Oceania	89.22 (38.40–140.04)	97.13 (40.10–154.16)	9% (−52–147%)	97.13 (40.10–154.16)	9% (−52–147%)	High
**Oceania**	**Oceania**	**Southeast Asia, East Asia, and Oceania**	**2.14 (0.00–7.27)**	**2.16 (0.00–7.57)**	**1% (−97–3132%)**	**13.61 (0.00–36.42)**	**536% (−66–11 777%)**	**NA**
American Samoa	Oceania	Southeast Asia, East Asia, and Oceania	0.59 (0.00–3.06)	0.61 (0.00–3.24)	4% (−100–42 717%)	56.66 (9.43–103.89)	9535% (33–698 027%)	Low
Cook Islands	Oceania	Southeast Asia, East Asia, and Oceania	0.59 (0.00–3.23)	NA	NA	NA	NA	Low
Fiji	Oceania	Southeast Asia, East Asia, and Oceania	4.77 (0.00–13.15)	5.04 (0.00–13.64)	6% (−91–1123%)	29.42 (0.00–60.46)	517% (−21–4684%)	Mid
Guam	Oceania	Southeast Asia, East Asia, and Oceania	0.83 (0.00–4.23)	0.83 (0.00–4.15)	0% (−100–31 488%)	71.44 (28.36–114.51)	8554% (33–561 732%)	Low
Kiribati	Oceania	Southeast Asia, East Asia, and Oceania	1.42 (0.00–7.18)	1.54 (0.00–8.09)	9% (−100–38 774%)	8.41 (0.00–39.53)	493% (−98–143 950%)	Mid
Marshall Islands	Oceania	Southeast Asia, East Asia, and Oceania	3.02 (0.00–10.39)	3.05 (0.00–9.80)	1% (−96–2624%)	18.35 (0.00–48.60)	507% (−68–11 439%)	Mid
Micronesia (Federated States of)	Oceania	Southeast Asia, East Asia, and Oceania	2.08 (0.00–8.60)	2.20 (0.00–8.94)	6% (−99–8411%)	12.54 (0.00–41.17)	504% (−88–29 266%)	Mid
Nauru	Oceania	Southeast Asia, East Asia, and Oceania	4.54 (0.00–13.29)	NA	NA	NA	NA	Mid
Niue	Oceania	Southeast Asia, East Asia, and Oceania	0.40 (0.00–2.46)	NA	NA	NA	NA	Low
Northern Mariana Islands	Oceania	Southeast Asia, East Asia, and Oceania	0.45 (0.00–2.52)	0.47 (0.00–2.65)	5% (−100–70 038%)	41.30 (8.36–74.24)	9064% (−14–976 452%)	Low
Palau	Oceania	Southeast Asia, East Asia, and Oceania	6.22 (0.00–15.50)	NA	NA	NA	NA	Mid
Papua New Guinea	Oceania	Southeast Asia, East Asia, and Oceania	2.01 (0.00–8.48)	2.08 (0.00–8.70)	4% (−99–9348%)	11.97 (0.00–39.89)	494% (−89–31 355%)	Mid
Samoa	Oceania	Southeast Asia, East Asia, and Oceania	2.91 (0.00–9.72)	2.93 (0.00–9.96)	1% (−96–2783%)	17.41 (0.00–47.81)	498% (−68–11 000%)	Mid
Solomon Islands	Oceania	Southeast Asia, East Asia, and Oceania	1.54 (0.00–8.92)	1.62 (0.00–8.14)	6% (−100–55 565%)	8.96 (0.00–36.79)	483% (−98–178 399%)	Mid
Tokelau	Oceania	Southeast Asia, East Asia, and Oceania	0.37 (0.00–2.53)	NA	NA	NA	NA	Low
Tonga	Oceania	Southeast Asia, East Asia, and Oceania	3.19 (0.00–10.43)	3.19 (0.00–10.47)	0% (−96–2404%)	19.03 (0.00–48.81)	497% (−62–9287%)	Mid
Tuvalu	Oceania	Southeast Asia, East Asia, and Oceania	2.59 (0.00–9.21)	NA	NA	NA	NA	Mid
Vanuatu	Oceania	Southeast Asia, East Asia, and Oceania	0.10 (0.00–1.32)	0.11 (0.00–1.82)	6% (−100–39 923 321 791%)	10.23 (0.00–40.61)	9882% (−100–2 182 121 053%)	Low
**Southeast Asia**	**Southeast Asia**	**Southeast Asia, East Asia, and Oceania**	**0.52 (0.00–1.72)**	**0.55 (0.00–1.81)**	**5% (−96–2642%)**	**50.53 (27.88–73.18)**	**9596% (828–101 193%)**	**NA**
Cambodia	Southeast Asia	Southeast Asia, East Asia, and Oceania	0.23 (0.00–1.77)	0.27 (0.00–2.11)	17% (−100–1 429 474%)	28.02 (0.00–68.14)	11 884% (−86–10 104 447%)	Low
Indonesia	Southeast Asia	Southeast Asia, East Asia, and Oceania	0.63 (0.00–3.26)	0.67 (0.00–3.60)	7% (−100–43 242%)	63.18 (9.91–116.46)	9888% (45–689 635%)	Low
Lao People's Democratic Republic	Southeast Asia	Southeast Asia, East Asia, and Oceania	0.27 (0.00–1.71)	0.31 (0.00–2.03)	17% (−100–246 852%)	29.67 (0.00–62.90)	10 950% (−54–2 637 701%)	Low
Malaysia	Southeast Asia	Southeast Asia, East Asia, and Oceania	0.87 (0.00–4.36)	0.92 (0.00–4.27)	5% (−100–23 208%)	81.58 (34.84–128.33)	9229% (65–525 798%)	Low
Maldives	Southeast Asia	Southeast Asia, East Asia, and Oceania	0.63 (0.00–3.33)	0.76 (0.00–3.94)	20% (−100–46 362%)	66.73 (20.60–112.85)	10 420% (42–779 335%)	Low
Mauritius	Southeast Asia	Southeast Asia, East Asia, and Oceania	0.74 (0.00–3.68)	0.76 (0.00–3.68)	3% (−100–25 307%)	68.68 (23.50–113.86)	9157% (66–514 984%)	Low
Myanmar	Southeast Asia	Southeast Asia, East Asia, and Oceania	0.25 (0.00–2.22)	0.26 (0.00–1.83)	4% (−100–1 838 453%)	26.17 (0.00–60.34)	10 243% (−96–27 117 132%)	Low
Philippines	Southeast Asia	Southeast Asia, East Asia, and Oceania	0.30 (0.00–2.17)	0.31 (0.00–2.15)	4% (−100–510 812%)	28.05 (0.00–57.59)	9201% (−82–4 941 261%)	Low
Seychelles	Southeast Asia	Southeast Asia, East Asia, and Oceania	0.93 (0.00–4.25)	0.99 (0.00–4.67)	6% (−99–18 152%)	85.71 (37.40–134.03)	9085% (152–334 587%)	Low
Sri Lanka	Southeast Asia	Southeast Asia, East Asia, and Oceania	0.52 (0.00–3.00)	0.56 (0.00–2.92)	6% (−100–61 063%)	51.50 (10.06–92.94)	9728% (−19–1 189 765%)	Low
Thailand	Southeast Asia	Southeast Asia, East Asia, and Oceania	0.80 (0.00–4.34)	0.87 (0.00–4.50)	9% (−100–48 148%)	77.26 (18.74–135.78)	9596% (7–879 675%)	Low
Timor–Leste	Southeast Asia	Southeast Asia, East Asia, and Oceania	0.19 (0.00–1.55)	0.19 (0.00–1.40)	1% (−100–1 395 890%)	20.22 (0.00–52.12)	10 556% (−93–16 419 575%)	Low
Viet Nam	Southeast Asia	Southeast Asia, East Asia, and Oceania	0.38 (0.00–2.26)	0.41 (0.00–2.35)	7% (−100–99 817%)	39.03 (1.74–76.32)	10 119% (−32–1 524 634%)	Low
**Sub‐Saharan Africa**	**NA**	**Sub‐Saharan Africa**	**0.59 (0.00–1.26)**	**0.53 (0.00–1.16)**	**−10% (−82–356%)**	**22.98 (12.99–32.98)**	**3772% (1058–12 846%)**	**NA**
**Central Sub‐Saharan Africa**	**Central Sub‐Saharan Africa**	**Sub‐Saharan Africa**	**0.15 (0.00–1.15)**	**0.16 (0.00–1.20)**	**12% (−100–1 193 143%)**	**17.18 (0.00–38.55)**	**11 604% (−88–11 853 404%)**	**NA**
Angola	Central Sub‐Saharan Africa	Sub‐Saharan Africa	0.28 (0.00–2.02)	0.31 (0.00–1.96)	10% (−100–366 008%)	30.99 (0.00–67.70)	10 856% (−79–5 672 206%)	Low
Central African Republic	Central Sub‐Saharan Africa	Sub‐Saharan Africa	0.05 (0.00–0.60)	0.07 (0.00–0.60)	30% (−100–54 000 547%)	9.21 (0.00–40.21)	17 216% (−100–932 286 561%)	Low
Congo	Central Sub‐Saharan Africa	Sub‐Saharan Africa	0.27 (0.00–1.84)	0.30 (0.00–2.26)	15% (−100–724 861%)	29.75 (0.00–69.46)	11 126% (−75–4 965 658%)	Low
Democratic Republic of the Congo	Central Sub‐Saharan Africa	Sub‐Saharan Africa	0.08 (0.00–1.45)	0.09 (0.00–1.48)	19% (−100–1 878 247 770 913%)	10.40 (0.00–38.55)	13 437% (−100–1 021 054 838 087%)	Low
Equatorial Guinea	Central Sub‐Saharan Africa	Sub‐Saharan Africa	0.69 (0.00–3.45)	0.80 (0.00–3.96)	17% (−100–32 554%)	73.06 (23.40–122.73)	10 554% (78–636 781%)	Low
Gabon	Central Sub‐Saharan Africa	Sub‐Saharan Africa	0.65 (0.00–3.51)	0.72 (0.00–3.67)	10% (−100–46 047%)	65.73 (17.61–113.84)	10 020% (16–885 236%)	Low
**Eastern Sub‐Saharan Africa**	**Eastern Sub‐Saharan Africa**	**Sub‐Saharan Africa**	**0.17 (0.00–0.83)**	**0.19 (0.00–0.76)**	**13% (−99–14 623%)**	**20.92 (6.13–35.72)**	**12 159% (138–630 372%)**	**NA**
Burundi	Eastern Sub‐Saharan Africa	Sub‐Saharan Africa	0.06 (0.00–1.18)	0.08 (0.00–1.66)	44% (−100–100 791 364 296 130%)	8.84 (0.00–32.59)	15 280% (−100–5 436 232 486 624%)	Low
Comoros	Eastern Sub‐Saharan Africa	Sub‐Saharan Africa	0.18 (0.00–2.09)	0.22 (0.00–2.01)	28% (−100–94 626 382%)	22.79 (0.00–61.01)	12 897% (−100–811 525 730%)	Low
Djibouti	Eastern Sub‐Saharan Africa	Sub‐Saharan Africa	0.31 (0.00–2.11)	0.37 (0.00–2.63)	21% (−100–546 817%)	35.88 (0.00–79.05)	11 539% (−70–4 579 208%)	Low
Eritrea	Eastern Sub‐Saharan Africa	Sub‐Saharan Africa	0.11 (0.00–1.05)	0.15 (0.00–1.54)	37% (−100–52 052 139%)	15.83 (0.00–50.25)	14 585% (−98–116 771 621%)	Low
Ethiopia	Eastern Sub‐Saharan Africa	Sub‐Saharan Africa	0.20 (0.00–2.16)	0.23 (0.00–2.01)	17% (−100–32 134 919%)	27.35 (0.00–75.42)	13 702% (−99–320 765 241%)	Low
Kenya	Eastern Sub‐Saharan Africa	Sub‐Saharan Africa	0.28 (0.00–2.37)	0.33 (0.00–2.23)	17% (−100–1 532 127%)	36.76 (0.00–90.16)	13 038% (−94–26 983 669%)	Low
Madagascar	Eastern Sub‐Saharan Africa	Sub‐Saharan Africa	0.07 (0.00–0.76)	0.08 (0.00–0.83)	22% (−100–115 099 162%)	9.14 (0.00–36.92)	13 535% (−100–618 156 609%)	Low
Malawi	Eastern Sub‐Saharan Africa	Sub‐Saharan Africa	0.10 (0.00–2.08)	0.12 (0.00–1.23)	22% (−100–290 445 842 663%)	13.71 (0.00–45.14)	13 499% (−100–5 094 426 064 011%)	Low
Mozambique	Eastern Sub‐Saharan Africa	Sub‐Saharan Africa	0.07 (0.00–0.75)	0.11 (0.00–1.45)	49% (−100–486 727 781%)	11.87 (0.00–46.02)	15 776% (−99–202 080 917%)	Low
Rwanda	Eastern Sub‐Saharan Africa	Sub‐Saharan Africa	0.06 (0.00–0.31)	0.06 (0.00–0.32)	7% (−100–45 535%)	5.03 (0.84–9.21)	8733% (4–750 975%)	Low
Somalia	Eastern Sub‐Saharan Africa	Sub‐Saharan Africa	0.04 (0.00–0.43)	0.04 (0.00–0.36)	3% (−100–12 083 992%)	5.56 (0.00–22.67)	13 087% (−99–197 480 278%)	Low
South Sudan	Eastern Sub‐Saharan Africa	Sub‐Saharan Africa	0.08 (0.00–0.69)	0.10 (0.00–1.23)	21% (−100–70 095 010%)	11.03 (0.00–42.51)	13 066% (−94–31 397 064%)	Low
Uganda	Eastern Sub‐Saharan Africa	Sub‐Saharan Africa	0.15 (0.00–2.76)	0.17 (0.00–1.67)	11% (−100–24 019 702 614%)	18.04 (0.00–51.48)	11 683% (−100–328 958 630 906%)	Low
United Republic of Tanzania	Eastern Sub‐Saharan Africa	Sub‐Saharan Africa	0.27 (0.00–1.65)	0.27 (0.00–1.49)	1% (−100–81 161%)	26.22 (3.11–49.32)	9492% (−42–1 577 529%)	Low
Zambia	Eastern Sub‐Saharan Africa	Sub‐Saharan Africa	0.19 (0.00–1.59)	0.22 (0.00–1.71)	13% (−100–2 207 382%)	23.44 (0.00–59.82)	11 972% (−92–18 712 347%)	Low
**Southern Sub‐Saharan Africa**	**Southern Sub‐Saharan Africa**	**Sub‐Saharan Africa**	**5.86 (0.00–13.16)**	**6.29 (0.00–13.92)**	**7% (−81–511%)**	**35.13 (11.65–58.60)**	**499% (46–2365%)**	**NA**
Botswana	Southern Sub‐Saharan Africa	Sub‐Saharan Africa	10.57 (0.00–23.80)	11.08 (0.00–24.21)	5% (−81–488%)	68.25 (39.42–97.07)	546% (72–2320%)	Mid
Eswatini	Southern Sub‐Saharan Africa	Sub‐Saharan Africa	5.59 (0.00–14.59)	6.57 (0.00–17.13)	18% (−88–1045%)	34.89 (1.41–68.36)	524% (−4–3973%)	Mid
Lesotho	Southern Sub‐Saharan Africa	Sub‐Saharan Africa	2.25 (0.00–9.47)	2.53 (0.00–9.07)	12% (−98–6790%)	13.28 (0.00–40.77)	489% (−87–26 640%)	Mid
Namibia	Southern Sub‐Saharan Africa	Sub‐Saharan Africa	0.34 (0.00–2.15)	0.37 (0.00–2.20)	9% (−100–165 085%)	33.79 (1.07–66.51)	9892% (−57–2 316 372%)	Low
South Africa	Southern Sub‐Saharan Africa	Sub‐Saharan Africa	7.09 (0.00–17.23)	8.02 (0.00–19.36)	13% (−85–745%)	43.10 (9.08–77.12)	508% (19–3010%)	Mid
Zimbabwe	Southern Sub‐Saharan Africa	Sub‐Saharan Africa	1.96 (0.00–8.48)	2.18 (0.00–8.35)	12% (−99–8699%)	11.67 (0.00–40.02)	496% (−90–36 748%)	Mid
**Western Sub‐Saharan Africa**	**Western Sub‐Saharan Africa**	**Sub‐Saharan Africa**	**0.22 (0.00–0.96)**	**0.25 (0.00–1.16)**	**12% (−99–15 354%)**	**24.74 (7.60–41.88)**	**11 018% (274–330 594%)**	**NA**
Benin	Western Sub‐Saharan Africa	Sub‐Saharan Africa	0.22 (0.00–2.06)	0.27 (0.00–2.37)	23% (−100–10 850 005%)	27.57 (0.00–71.38)	12 408% (−97–60 523 510%)	Low
Burkina Faso	Western Sub‐Saharan Africa	Sub‐Saharan Africa	0.20 (0.00–1.87)	0.24 (0.00–2.44)	20% (−100–23 691 376%)	26.40 (0.00–74.21)	12 900% (−97–58 827 909%)	Low
Cabo Verde	Western Sub‐Saharan Africa	Sub‐Saharan Africa	0.55 (0.00–4.00)	0.60 (0.00–3.97)	9% (−100–469 969%)	63.62 (0.00–144.37)	11 395% (−80–6 657 741%)	Low
Cameroon	Western Sub‐Saharan Africa	Sub‐Saharan Africa	0.29 (0.00–2.14)	0.35 (0.00–3.10)	22% (−100–3 133 419%)	38.03 (0.00–95.90)	13 124% (−82–9 875 897%)	Low
Chad	Western Sub‐Saharan Africa	Sub‐Saharan Africa	0.13 (0.00–1.24)	0.16 (0.00–1.57)	18% (−100–28 763 520%)	17.67 (0.00–55.12)	13 329% (−98–79 409 457%)	Low
Côte d'Ivoire	Western Sub‐Saharan Africa	Sub‐Saharan Africa	0.32 (0.00–2.22)	0.39 (0.00–2.81)	21% (−100–645 717%)	38.41 (0.00–86.99)	11 864% (−71–4 990 016%)	Low
Gambia	Western Sub‐Saharan Africa	Sub‐Saharan Africa	0.25 (0.00–2.77)	0.31 (0.00–3.27)	22% (−100–141 708 807%)	35.08 (0.00–95.91)	13 910% (−99–374 856 465%)	Low
Ghana	Western Sub‐Saharan Africa	Sub‐Saharan Africa	0.43 (0.00–2.88)	0.51 (0.00–3.76)	20% (−100–624 129%)	50.38 (0.00–113.23)	11 682% (−67–4 192 282%)	Low
Guinea	Western Sub‐Saharan Africa	Sub‐Saharan Africa	0.21 (0.00–1.86)	0.26 (0.00–2.45)	23% (−100–12 364 708%)	29.21 (0.00–80.53)	13 756% (−95–42 517 796%)	Low
Guinea–Bissau	Western Sub‐Saharan Africa	Sub‐Saharan Africa	0.16 (0.00–2.13)	0.20 (0.00–2.41)	28% (−100–2 135 061 634%)	21.87 (0.00–63.90)	13 767% (−100–4 379 075 529%)	Low
Liberia	Western Sub‐Saharan Africa	Sub‐Saharan Africa	0.14 (0.00–1.32)	0.20 (0.00–2.01)	38% (−100–29 765 244%)	24.72 (0.00–78.37)	17 124% (−96–80 750 835%)	Low
Mali	Western Sub‐Saharan Africa	Sub‐Saharan Africa	0.19 (0.00–1.67)	0.22 (0.00–2.19)	19% (−100–15 944 027%)	25.13 (0.00–73.84)	13 303% (−96–46 146 548%)	Low
Mauritania	Western Sub‐Saharan Africa	Sub‐Saharan Africa	0.31 (0.00–2.41)	0.36 (0.00–2.93)	15% (−100–2 082 642%)	36.27 (0.00–85.09)	11 517% (−88–10 903 472%)	Low
Niger	Western Sub‐Saharan Africa	Sub‐Saharan Africa	0.08 (0.00–1.48)	0.08 (0.00–0.97)	1% (−100–200 823 461 903%)	8.90 (0.00–38.09)	11 376% (−100–1 145 271 402 171%)	Low
Nigeria	Western Sub‐Saharan Africa	Sub‐Saharan Africa	0.20 (0.00–1.64)	0.23 (0.00–1.94)	17% (−100–3 378 170%)	21.24 (0.00–52.59)	10 554% (−93–16 755 062%)	Low
Sao Tome and Principe	Western Sub‐Saharan Africa	Sub‐Saharan Africa	0.30 (0.00–2.25)	0.36 (0.00–3.14)	22% (−100–2 928 709%)	37.48 (0.00–92.64)	12 532% (−85–10 570 925%)	Low
Senegal	Western Sub‐Saharan Africa	Sub‐Saharan Africa	0.25 (0.00–2.12)	0.28 (0.00–2.08)	12% (−100–1 910 129%)	32.39 (0.00–82.11)	12 721% (−93–24 111 206%)	Low
