# Supplementary material for: Economic Impact of Periodontitis: Global, Regional and National Estimates of Periodontal Expenditure, With Forecasts to 2050
Source: J Periodontal Res. 2026 May 9;61(5):518–59. doi: 10.1111/jre.70104 (PMC13378222; doi:10.1111/jre.70104)

Supplementary Methods 1: R Code for Monte Carlo Model (please see Github repository for full code)

# ==============================================================================

# Cost Model Function — Optimised Version with Cleanup

# ==============================================================================

source("./99_functions.R")

# ============================================================================

# Load packages

# ============================================================================

library(MCMCpack)

library(tidyverse)

library(data.table)

# ==============================================================================

# Calling the function

# ==============================================================================

# Example file paths (adjust if needed)

countries_path <- "./data/combined_country_input.csv"

procedure_lookup_high_path <- "./data/procedure_lookup.csv"

procedure_lookup_mid_path <- "./data/procedure_lookup.csv"

# procedure_lookup_high_path <- "./data/procedure_lookup_increased_maintenance.csv" #for various scenarios

# procedure_lookup_mid_path <- "./data/procedure_lookup_increased_maintenance.csv"

procedure_lookup_low_path <- "./data/procedure_lookup_low_scenario.csv"

procedure_lookup_WHO_target_path <- "./data/procedure_lookup.csv"

# procedure_lookup_low_path <- "./data/procedure_lookup_low_scenario_increased_maintenance.csv"

tx_split_high_path <- "./data/tx_split.csv"

tx_split_mid_path <- "./data/tx_split_mid_scenario.csv"

tx_split_low_path <- "./data/tx_split_low_scenario.csv"

tx_split_WHO_target_path <- "./data/tx_split_WHO_target_scenario.csv"

severity_split_path <- "./data/severity_split.csv"

# Where to write outputs

output_dir <- "outputs"

n_sims <- 12000

# Call the new scenario runner

run_cost_model_scenarios(

countries_path = countries_path,

procedure_lookup_high_path = procedure_lookup_high_path,

procedure_lookup_mid_path = procedure_lookup_mid_path,

procedure_lookup_low_path = procedure_lookup_low_path,

tx_split_high_path = tx_split_high_path,

tx_split_mid_path = tx_split_mid_path,

tx_split_low_path = tx_split_low_path,

severity_split_path = severity_split_path,

n_sims = n_sims,

output_dir = output_dir,

scenarios = c("low", "mid", "high", "WHO_target"),

# scenarios = c("low", "mid", "high"),

slice_size = 999

)

Supplementary Methods 2: Detailed Search Strategy for Country Level Expenditure

| *Country* | *Search string* | *Official Language(s)* | *Translation of "Periodontitis"* | *Translation of "Expenditure"* | *Translation of "Ministry of Health"* | *[No blanks] National Dental/Health Insurance Scheme* |
| --- | --- | --- | --- | --- | --- | --- |
| Afghanistan | (Afghanistan) AND (periodont* OR پیریڈونٹائٹس) AND (cost OR expenditure OR spending OR revenue OR reimbursement OR مصارف (Masaref)) AND (health accounts OR ministry of health OR national OR federal OR insurance OR registry OR register OR database OR data OR dataset OR pubmed OR survey OR interview OR panel OR وزارت صحت عامه OR Insurance) | Pashto, Dari | پیریڈونٹائٹس | مصارف (Masaref) | وزارت صحت عامه | Insurance |
| Albania | (Albania) AND (periodont* OR Periodontiti) AND (cost OR expenditure OR spending OR revenue OR reimbursement OR Shpenzime) AND (health accounts OR ministry of health OR national OR federal OR insurance OR registry OR register OR database OR data OR dataset OR pubmed OR survey OR interview OR panel OR Ministria e Shëndetësisë dhe Mbrojtjes Sociale OR Insurance) | Albanian | Periodontiti | Shpenzime | Ministria e Shëndetësisë dhe Mbrojtjes Sociale | Insurance |
| Algeria | (Algeria) AND (periodont* OR التهاب دواعم السن) AND (cost OR expenditure OR spending OR revenue OR reimbursement OR النفقات (Al-Nafaqat)) AND (health accounts OR ministry of health OR national OR federal OR insurance OR registry OR register OR database OR data OR dataset OR pubmed OR survey OR interview OR panel OR وزارة الصحة OR Insurance) | Arabic | التهاب دواعم السن | النفقات (Al-Nafaqat) | وزارة الصحة | Insurance |
| Andorra | (Andorra) AND (periodont* OR Periodontitis) AND (cost OR expenditure OR spending OR revenue OR reimbursement OR Despesa) AND (health accounts OR ministry of health OR national OR federal OR insurance OR registry OR register OR database OR data OR dataset OR pubmed OR survey OR interview OR panel OR Ministeri de Salut OR Insurance) | Catalan | Periodontitis | Despesa | Ministeri de Salut | Insurance |
| Angola | (Angola) AND (periodont* OR Periodontite) AND (cost OR expenditure OR spending OR revenue OR reimbursement OR Despesa) AND (health accounts OR ministry of health OR national OR federal OR insurance OR registry OR register OR database OR data OR dataset OR pubmed OR survey OR interview OR panel OR Ministério da Saúde OR Insurance) | Portuguese | Periodontite | Despesa | Ministério da Saúde | Insurance |
| Antigua and Barbuda | (Antigua and Barbuda) AND (periodont* OR Periodontitis) AND (cost OR expenditure OR spending OR revenue OR reimbursement OR Expenditure) AND (health accounts OR ministry of health OR national OR federal OR insurance OR registry OR register OR database OR data OR dataset OR pubmed OR survey OR interview OR panel OR Ministry of Health, Wellness & The Environment OR Insurance) | English | Periodontitis | Expenditure | Ministry of Health, Wellness & The Environment | Insurance |
| Argentina | (Argentina) AND (periodont* OR Periodontitis) AND (cost OR expenditure OR spending OR revenue OR reimbursement OR Gasto) AND (health accounts OR ministry of health OR national OR federal OR insurance OR registry OR register OR database OR data OR dataset OR pubmed OR survey OR interview OR panel OR Ministerio de Salud OR Programa Médico Obligatorio (PMO)) | Spanish | Periodontitis | Gasto | Ministerio de Salud | Programa Médico Obligatorio (PMO) |
| Armenia | (Armenia) AND (periodont* OR Պերիօդոնտիտ) AND (cost OR expenditure OR spending OR revenue OR reimbursement OR Ծախս (Tsakhs)) AND (health accounts OR ministry of health OR national OR federal OR insurance OR registry OR register OR database OR data OR dataset OR pubmed OR survey OR interview OR panel OR Առողջապահության նախարարություն OR Insurance) | Armenian | Պերիօդոնտիտ | Ծախս (Tsakhs) | Առողջապահության նախարարություն | Insurance |
| Australia | (Australia) AND (periodont* OR Periodontitis) AND (cost OR expenditure OR spending OR revenue OR reimbursement OR Expenditure) AND (health accounts OR ministry of health OR national OR federal OR insurance OR registry OR register OR database OR data OR dataset OR pubmed OR survey OR interview OR panel OR Department of Health OR Medicare) | English | Periodontitis | Expenditure | Department of Health | Medicare |
| Austria | (Austria) AND (periodont* OR Parodontitis) AND (cost OR expenditure OR spending OR revenue OR reimbursement OR Ausgaben) AND (health accounts OR ministry of health OR national OR federal OR insurance OR registry OR register OR database OR data OR dataset OR pubmed OR survey OR interview OR panel OR Bundesministerium für Soziales, Gesundheit, Pflege und Konsumentenschutz OR Österreichische Gesundheitskasse (ÖGK)) | German | Parodontitis | Ausgaben | Bundesministerium für Soziales, Gesundheit, Pflege und Konsumentenschutz | Österreichische Gesundheitskasse (ÖGK) |
| Azerbaijan | (Azerbaijan) AND (periodont* OR Periodontit) AND (cost OR expenditure OR spending OR revenue OR reimbursement OR Xərclər) AND (health accounts OR ministry of health OR national OR federal OR insurance OR registry OR register OR database OR data OR dataset OR pubmed OR survey OR interview OR panel OR Səhiyyə Nazirliyi OR Insurance) | Azerbaijani | Periodontit | Xərclər | Səhiyyə Nazirliyi | Insurance |
| Bahamas | (Bahamas) AND (periodont* OR Periodontitis) AND (cost OR expenditure OR spending OR revenue OR reimbursement OR Expenditure) AND (health accounts OR ministry of health OR national OR federal OR insurance OR registry OR register OR database OR data OR dataset OR pubmed OR survey OR interview OR panel OR Ministry of Health OR Insurance) | English | Periodontitis | Expenditure | Ministry of Health | Insurance |
| Bahrain | (Bahrain) AND (periodont* OR التهاب دواعم السن) AND (cost OR expenditure OR spending OR revenue OR reimbursement OR النفقات (Al-Nafaqat)) AND (health accounts OR ministry of health OR national OR federal OR insurance OR registry OR register OR database OR data OR dataset OR pubmed OR survey OR interview OR panel OR وزارة الصحة OR Insurance) | Arabic | التهاب دواعم السن | النفقات (Al-Nafaqat) | وزارة الصحة | Insurance |
| Bangladesh | (Bangladesh) AND (periodont* OR পেরিওডন্টাইটিস) AND (cost OR expenditure OR spending OR revenue OR reimbursement OR ব্যয় (Byay)) AND (health accounts OR ministry of health OR national OR federal OR insurance OR registry OR register OR database OR data OR dataset OR pubmed OR survey OR interview OR panel OR স্বাস্থ্য ও পরিবার কল্যাণ মন্ত্রণালয় OR Insurance) | Bengali | পেরিওডন্টাইটিস | ব্যয় (Byay) | স্বাস্থ্য ও পরিবার কল্যাণ মন্ত্রণালয় | Insurance |
| Barbados | (Barbados) AND (periodont* OR Periodontitis) AND (cost OR expenditure OR spending OR revenue OR reimbursement OR Expenditure) AND (health accounts OR ministry of health OR national OR federal OR insurance OR registry OR register OR database OR data OR dataset OR pubmed OR survey OR interview OR panel OR Ministry of Health and Wellness OR Insurance) | English | Periodontitis | Expenditure | Ministry of Health and Wellness | Insurance |
| Belarus | (Belarus) AND (periodont* OR Перыядантыт) AND (cost OR expenditure OR spending OR revenue OR reimbursement OR Выдаткі (Vydatki)) AND (health accounts OR ministry of health OR national OR federal OR insurance OR registry OR register OR database OR data OR dataset OR pubmed OR survey OR interview OR panel OR Министэрства аховы здароўя OR Insurance) | Belarusian, Russian | Перыядантыт | Выдаткі (Vydatki) | Министэрства аховы здароўя | Insurance |
| Belgium | (Belgium) AND (periodont* OR Parodontitis) AND (cost OR expenditure OR spending OR revenue OR reimbursement OR Uitgaven / Dépenses / Ausgaben) AND (health accounts OR ministry of health OR national OR federal OR insurance OR registry OR register OR database OR data OR dataset OR pubmed OR survey OR interview OR panel OR Service public fédéral Santé publique, Sécurité de la Chaîne alimentaire et Environnement OR RIZIV/INAMI) | Dutch, French, German | Parodontitis | Uitgaven / Dépenses / Ausgaben | Service public fédéral Santé publique, Sécurité de la Chaîne alimentaire et Environnement | RIZIV/INAMI |
| Belize | (Belize) AND (periodont* OR Periodontitis) AND (cost OR expenditure OR spending OR revenue OR reimbursement OR Expenditure) AND (health accounts OR ministry of health OR national OR federal OR insurance OR registry OR register OR database OR data OR dataset OR pubmed OR survey OR interview OR panel OR Ministry of Health and Wellness OR Insurance) | English | Periodontitis | Expenditure | Ministry of Health and Wellness | Insurance |
| Benin | (Benin) AND (periodont* OR Parodontite) AND (cost OR expenditure OR spending OR revenue OR reimbursement OR Dépenses) AND (health accounts OR ministry of health OR national OR federal OR insurance OR registry OR register OR database OR data OR dataset OR pubmed OR survey OR interview OR panel OR Ministère de la Santé OR Insurance) | French | Parodontite | Dépenses | Ministère de la Santé | Insurance |
| Bhutan | (Bhutan) AND (periodont* OR Periodontitis) AND (cost OR expenditure OR spending OR revenue OR reimbursement OR འབད་དོག (Beydok)) AND (health accounts OR ministry of health OR national OR federal OR insurance OR registry OR register OR database OR data OR dataset OR pubmed OR survey OR interview OR panel OR གསོ་བའི་ལས་ཁུངས། OR Insurance) | Dzongkha | Periodontitis | འབད་དོག (Beydok) | གསོ་བའི་ལས་ཁུངས། | Insurance |
| Bolivia (Plurinational State of) | (Bolivia (Plurinational State of)) AND (periodont* OR Periodontitis) AND (cost OR expenditure OR spending OR revenue OR reimbursement OR Gasto) AND (health accounts OR ministry of health OR national OR federal OR insurance OR registry OR register OR database OR data OR dataset OR pubmed OR survey OR interview OR panel OR Ministerio de Salud y Deportes OR Insurance) | Spanish | Periodontitis | Gasto | Ministerio de Salud y Deportes | Insurance |
| Bosnia and Herzegovina | (Bosnia and Herzegovina) AND (periodont* OR Parodontitis) AND (cost OR expenditure OR spending OR revenue OR reimbursement OR Trošak) AND (health accounts OR ministry of health OR national OR federal OR insurance OR registry OR register OR database OR data OR dataset OR pubmed OR survey OR interview OR panel OR Federalno ministarstvo zdravstva OR Insurance) | Bosnian, Croatian, Serbian | Parodontitis | Trošak | Federalno ministarstvo zdravstva | Insurance |
| Botswana | (Botswana) AND (periodont* OR Periodontitis) AND (cost OR expenditure OR spending OR revenue OR reimbursement OR Expenditure / Ditshenyegelo) AND (health accounts OR ministry of health OR national OR federal OR insurance OR registry OR register OR database OR data OR dataset OR pubmed OR survey OR interview OR panel OR Ministry of Health and Wellness OR Insurance) | English, Tswana | Periodontitis | Expenditure / Ditshenyegelo | Ministry of Health and Wellness | Insurance |
| Brazil | (Brazil) AND (periodont* OR Periodontite) AND (cost OR expenditure OR spending OR revenue OR reimbursement OR Despesa) AND (health accounts OR ministry of health OR national OR federal OR insurance OR registry OR register OR database OR data OR dataset OR pubmed OR survey OR interview OR panel OR Ministério da Saúde OR Sistema Único de Saúde (SUS)) | Periodontite | Despesa | Ministério da Saúde | Sistema Único de Saúde (SUS) |  |
| Brunei Darussalam | (Brunei Darussalam) AND (periodont* OR Periodontitis) AND (cost OR expenditure OR spending OR revenue OR reimbursement OR Perbelanjaan) AND (health accounts OR ministry of health OR national OR federal OR insurance OR registry OR register OR database OR data OR dataset OR pubmed OR survey OR interview OR panel OR Kementerian Kesihatan OR Insurance) | Malay | Periodontitis | Perbelanjaan | Kementerian Kesihatan | Insurance |
| Bulgaria | (Bulgaria) AND (periodont* OR Пародонтит) AND (cost OR expenditure OR spending OR revenue OR reimbursement OR Разходи (Razkhodi)) AND (health accounts OR ministry of health OR national OR federal OR insurance OR registry OR register OR database OR data OR dataset OR pubmed OR survey OR interview OR panel OR Министерство на здравеопазването OR Insurance) | Bulgarian | Пародонтит | Разходи (Razkhodi) | Министерство на здравеопазването | Insurance |
| Burkina Faso | (Burkina Faso) AND (periodont* OR Parodontite) AND (cost OR expenditure OR spending OR revenue OR reimbursement OR Dépenses) AND (health accounts OR ministry of health OR national OR federal OR insurance OR registry OR register OR database OR data OR dataset OR pubmed OR survey OR interview OR panel OR Ministère de la Santé OR Insurance) | French | Parodontite | Dépenses | Ministère de la Santé | Insurance |
| Burundi | (Burundi) AND (periodont* OR Parodontite) AND (cost OR expenditure OR spending OR revenue OR reimbursement OR Inyungu / Dépenses) AND (health accounts OR ministry of health OR national OR federal OR insurance OR registry OR register OR database OR data OR dataset OR pubmed OR survey OR interview OR panel OR Ministère de la Santé Publique et de la Lutte contre le SIDA OR Insurance) | Kirundi, French | Parodontite | Inyungu / Dépenses | Ministère de la Santé Publique et de la Lutte contre le SIDA | Insurance |
| Cabo Verde | (Cabo Verde) AND (periodont* OR Periodontite) AND (cost OR expenditure OR spending OR revenue OR reimbursement OR Despesa) AND (health accounts OR ministry of health OR national OR federal OR insurance OR registry OR register OR database OR data OR dataset OR pubmed OR survey OR interview OR panel OR Ministério da Saúde OR Insurance) | Portuguese | Periodontite | Despesa | Ministério da Saúde | Insurance |
| Cambodia | (Cambodia) AND (periodont* OR ប៉េរីអូដន់ទីត) AND (cost OR expenditure OR spending OR revenue OR reimbursement OR ការចំណាយ (Kar Chamnay)) AND (health accounts OR ministry of health OR national OR federal OR insurance OR registry OR register OR database OR data OR dataset OR pubmed OR survey OR interview OR panel OR ក្រសួងសុខាភិបាល OR Insurance) | Khmer | ប៉េរីអូដន់ទីត | ការចំណាយ (Kar Chamnay) | ក្រសួងសុខាភិបាល | Insurance |
| Cameroon | (Cameroon) AND (periodont* OR Parodontite) AND (cost OR expenditure OR spending OR revenue OR reimbursement OR Expenditure / Dépenses) AND (health accounts OR ministry of health OR national OR federal OR insurance OR registry OR register OR database OR data OR dataset OR pubmed OR survey OR interview OR panel OR Ministère de la Santé Publique OR Insurance) | English, French | Parodontite | Expenditure / Dépenses | Ministère de la Santé Publique | Insurance |
| Canada | (Canada) AND (periodont* OR Periodontitis) AND (cost OR expenditure OR spending OR revenue OR reimbursement OR Expenditure / Dépenses) AND (health accounts OR ministry of health OR national OR federal OR insurance OR registry OR register OR database OR data OR dataset OR pubmed OR survey OR interview OR panel OR Santé Canada OR Canada Health Act) | English, French | Periodontitis | Expenditure / Dépenses | Santé Canada | Canada Health Act |
| Central African Republic | (Central African Republic) AND (periodont* OR Parodontite) AND (cost OR expenditure OR spending OR revenue OR reimbursement OR Dépenses) AND (health accounts OR ministry of health OR national OR federal OR insurance OR registry OR register OR database OR data OR dataset OR pubmed OR survey OR interview OR panel OR Ministère de la Santé et de la Population OR Insurance) | Sango, French | Parodontite | Dépenses | Ministère de la Santé et de la Population | Insurance |
| Chad | (Chad) AND (periodont* OR Parodontite) AND (cost OR expenditure OR spending OR revenue OR reimbursement OR النفقات (Al-Nafaqat) / Dépenses) AND (health accounts OR ministry of health OR national OR federal OR insurance OR registry OR register OR database OR data OR dataset OR pubmed OR survey OR interview OR panel OR Ministère de la Santé Publique OR Insurance) | Arabic, French | Parodontite | النفقات (Al-Nafaqat) / Dépenses | Ministère de la Santé Publique | Insurance |
| Chile | (Chile) AND (periodont* OR Periodontitis) AND (cost OR expenditure OR spending OR revenue OR reimbursement OR Gasto) AND (health accounts OR ministry of health OR national OR federal OR insurance OR registry OR register OR database OR data OR dataset OR pubmed OR survey OR interview OR panel OR Ministerio de Salud OR Fondo Nacional de Salud (FONASA)) | Spanish | Periodontitis | Gasto | Ministerio de Salud | Fondo Nacional de Salud (FONASA) |
| China | (China) AND (periodont* OR 牙周炎) AND (cost OR expenditure OR spending OR revenue OR reimbursement OR 支出 (Zhīchū)) AND (health accounts OR ministry of health OR national OR federal OR insurance OR registry OR register OR database OR data OR dataset OR pubmed OR survey OR interview OR panel OR 国家卫生健康委员会 OR National Healthcare Security) | Chinese (Mandarin) | 牙周炎 | 支出 (Zhīchū) | 国家卫生健康委员会 | National Healthcare Security |
| Colombia | (Colombia) AND (periodont* OR Periodontitis) AND (cost OR expenditure OR spending OR revenue OR reimbursement OR Gasto) AND (health accounts OR ministry of health OR national OR federal OR insurance OR registry OR register OR database OR data OR dataset OR pubmed OR survey OR interview OR panel OR Ministerio de Salud y Protección Social OR Sistema General de Seguridad Social en Salud) | Spanish | Periodontitis | Gasto | Ministerio de Salud y Protección Social | Sistema General de Seguridad Social en Salud |
| Comoros | (Comoros) AND (periodont* OR Parodontite) AND (cost OR expenditure OR spending OR revenue OR reimbursement OR Matumizi / النفقات / Dépenses) AND (health accounts OR ministry of health OR national OR federal OR insurance OR registry OR register OR database OR data OR dataset OR pubmed OR survey OR interview OR panel OR Ministère de la Santé OR Insurance) | Comorian, Arabic, French | Parodontite | Matumizi / النفقات / Dépenses | Ministère de la Santé | Insurance |
| Congo | (Congo) AND (periodont* OR Parodontite) AND (cost OR expenditure OR spending OR revenue OR reimbursement OR Dépenses) AND (health accounts OR ministry of health OR national OR federal OR insurance OR registry OR register OR database OR data OR dataset OR pubmed OR survey OR interview OR panel OR Ministère de la Santé et de la Population OR Insurance) | French | Parodontite | Dépenses | Ministère de la Santé et de la Population | Insurance |
| Cook Islands | (Cook Islands) AND (periodont* OR Periodontitis) AND (cost OR expenditure OR spending OR revenue OR reimbursement OR Expenditure / Pēpē) AND (health accounts OR ministry of health OR national OR federal OR insurance OR registry OR register OR database OR data OR dataset OR pubmed OR survey OR interview OR panel OR Ministry of Health OR Insurance) | English, Cook Islands Māori | Periodontitis | Expenditure / Pēpē | Ministry of Health | Insurance |
| Costa Rica | (Costa Rica) AND (periodont* OR Periodontitis) AND (cost OR expenditure OR spending OR revenue OR reimbursement OR Gasto) AND (health accounts OR ministry of health OR national OR federal OR insurance OR registry OR register OR database OR data OR dataset OR pubmed OR survey OR interview OR panel OR Ministerio de Salud OR Caja Costarricense de Seguro Social (CCSS)) | Spanish | Periodontitis | Gasto | Ministerio de Salud | Caja Costarricense de Seguro Social (CCSS) |
| Côte d'Ivoire | (Côte d'Ivoire) AND (periodont* OR Parodontite) AND (cost OR expenditure OR spending OR revenue OR reimbursement OR Dépenses) AND (health accounts OR ministry of health OR national OR federal OR insurance OR registry OR register OR database OR data OR dataset OR pubmed OR survey OR interview OR panel OR Ministère de la Santé et de l'Hygiène Publique OR Insurance) | French | Parodontite | Dépenses | Ministère de la Santé et de l'Hygiène Publique | Insurance |
| Croatia | (Croatia) AND (periodont* OR Parodontitis) AND (cost OR expenditure OR spending OR revenue OR reimbursement OR Trošak) AND (health accounts OR ministry of health OR national OR federal OR insurance OR registry OR register OR database OR data OR dataset OR pubmed OR survey OR interview OR panel OR Ministarstvo zdravstva OR Hrvatski Zavod za Zdravstveno Osiguranje) | Croatian | Parodontitis | Trošak | Ministarstvo zdravstva | Hrvatski Zavod za Zdravstveno Osiguranje |
| Cuba | (Cuba) AND (periodont* OR Periodontitis) AND (cost OR expenditure OR spending OR revenue OR reimbursement OR Gasto) AND (health accounts OR ministry of health OR national OR federal OR insurance OR registry OR register OR database OR data OR dataset OR pubmed OR survey OR interview OR panel OR Ministerio de Salud Pública OR Servicio Nacional de Salud) | Spanish | Periodontitis | Gasto | Ministerio de Salud Pública | Servicio Nacional de Salud |
| Cyprus | (Cyprus) AND (periodont* OR Περιοδοντίτιδα) AND (cost OR expenditure OR spending OR revenue OR reimbursement OR Δαπάνη (Dapani) / Gider) AND (health accounts OR ministry of health OR national OR federal OR insurance OR registry OR register OR database OR data OR dataset OR pubmed OR survey OR interview OR panel OR Υπουργείο Υγείας OR Insurance) | Greek, Turkish | Περιοδοντίτιδα | Δαπάνη (Dapani) / Gider | Υπουργείο Υγείας | Insurance |
| Czechia | (Czechia) AND (periodont* OR Parodontit) AND (cost OR expenditure OR spending OR revenue OR reimbursement OR Výdaje) AND (health accounts OR ministry of health OR national OR federal OR insurance OR registry OR register OR database OR data OR dataset OR pubmed OR survey OR interview OR panel OR Ministerstvo zdravotnictví OR Všeobecná zdravotní pojišťovna (VZP)) | Czech | Parodontit | Výdaje | Ministerstvo zdravotnictví | Všeobecná zdravotní pojišťovna (VZP) |
| Democratic People's Republic of Korea | (Democratic People's Republic of Korea) AND (periodont* OR 치주염) AND (cost OR expenditure OR spending OR revenue OR reimbursement OR 지출 (Jichul)) AND (health accounts OR ministry of health OR national OR federal OR insurance OR registry OR register OR database OR data OR dataset OR pubmed OR survey OR interview OR panel OR 보건성 OR Insurance) | Korean | 치주염 | 지출 (Jichul) | 보건성 | Insurance |
| Democratic Republic of the Congo | (Democratic Republic of the Congo) AND (periodont* OR Parodontite) AND (cost OR expenditure OR spending OR revenue OR reimbursement OR Dépenses) AND (health accounts OR ministry of health OR national OR federal OR insurance OR registry OR register OR database OR data OR dataset OR pubmed OR survey OR interview OR panel OR Ministère de la Santé Publique OR Insurance) | French | Parodontite | Dépenses | Ministère de la Santé Publique | Insurance |
| Denmark | (Denmark) AND (periodont* OR Parodontitis) AND (cost OR expenditure OR spending OR revenue OR reimbursement OR Udgift) AND (health accounts OR ministry of health OR national OR federal OR insurance OR registry OR register OR database OR data OR dataset OR pubmed OR survey OR interview OR panel OR Sundhedsministeriet OR Sundhedsstyrelsen) | Danish | Parodontitis | Udgift | Sundhedsministeriet | Sundhedsstyrelsen |
| Djibouti | (Djibouti) AND (periodont* OR Parodontite) AND (cost OR expenditure OR spending OR revenue OR reimbursement OR النفقات / Dépenses) AND (health accounts OR ministry of health OR national OR federal OR insurance OR registry OR register OR database OR data OR dataset OR pubmed OR survey OR interview OR panel OR Ministère de la Santé OR Insurance) | Arabic, French | Parodontite | النفقات / Dépenses | Ministère de la Santé | Insurance |
| Dominica | (Dominica) AND (periodont* OR Periodontitis) AND (cost OR expenditure OR spending OR revenue OR reimbursement OR Expenditure) AND (health accounts OR ministry of health OR national OR federal OR insurance OR registry OR register OR database OR data OR dataset OR pubmed OR survey OR interview OR panel OR Ministry of Health, Wellness and Social Services OR Insurance) | English | Periodontitis | Expenditure | Ministry of Health, Wellness and Social Services | Insurance |
| Dominican Republic | (Dominican Republic) AND (periodont* OR Periodontitis) AND (cost OR expenditure OR spending OR revenue OR reimbursement OR Gasto) AND (health accounts OR ministry of health OR national OR federal OR insurance OR registry OR register OR database OR data OR dataset OR pubmed OR survey OR interview OR panel OR Ministerio de Salud Pública OR Seguro Nacional de Salud (SeNaSa)) | Spanish | Periodontitis | Gasto | Ministerio de Salud Pública | Seguro Nacional de Salud (SeNaSa) |
| Ecuador | (Ecuador) AND (periodont* OR Periodontitis) AND (cost OR expenditure OR spending OR revenue OR reimbursement OR Gasto) AND (health accounts OR ministry of health OR national OR federal OR insurance OR registry OR register OR database OR data OR dataset OR pubmed OR survey OR interview OR panel OR Ministerio de Salud Pública OR Instituto Ecuatoriano de Seguridad Social) | Spanish | Periodontitis | Gasto | Ministerio de Salud Pública | Instituto Ecuatoriano de Seguridad Social |
| Egypt | (Egypt) AND (periodont* OR التهاب دواعم السن) AND (cost OR expenditure OR spending OR revenue OR reimbursement OR النفقات (Al-Nafaqat)) AND (health accounts OR ministry of health OR national OR federal OR insurance OR registry OR register OR database OR data OR dataset OR pubmed OR survey OR interview OR panel OR وزارة الصحة والسكان OR Insurance) | Arabic | التهاب دواعم السن | النفقات (Al-Nafaqat) | وزارة الصحة والسكان | Insurance |
| El Salvador | (El Salvador) AND (periodont* OR Periodontitis) AND (cost OR expenditure OR spending OR revenue OR reimbursement OR Gasto) AND (health accounts OR ministry of health OR national OR federal OR insurance OR registry OR register OR database OR data OR dataset OR pubmed OR survey OR interview OR panel OR Ministerio de Salud OR Instituto Salvadoreño del Seguro Social) | Spanish | Periodontitis | Gasto | Ministerio de Salud | Instituto Salvadoreño del Seguro Social |
| Equatorial Guinea | (Equatorial Guinea) AND (periodont* OR Periodontitis) AND (cost OR expenditure OR spending OR revenue OR reimbursement OR Gasto / Dépenses / Despesa) AND (health accounts OR ministry of health OR national OR federal OR insurance OR registry OR register OR database OR data OR dataset OR pubmed OR survey OR interview OR panel OR Ministerio de Sanidad y Bienestar Social OR Insurance) | Spanish, French, Portuguese | Periodontitis | Gasto / Dépenses / Despesa | Ministerio de Sanidad y Bienestar Social | Insurance |
| Eritrea | (Eritrea) AND (periodont* OR Periodontitis) AND (cost OR expenditure OR spending OR revenue OR reimbursement OR ወጪ (Wechi) / النفقات / Expenditure) AND (health accounts OR ministry of health OR national OR federal OR insurance OR registry OR register OR database OR data OR dataset OR pubmed OR survey OR interview OR panel OR ሚኒስትሪ ጥዕና OR Insurance) | Tigrinya, Arabic, English | Periodontitis | ወጪ (Wechi) / النفقات / Expenditure | ሚኒስትሪ ጥዕና | Insurance |
| Estonia | (Estonia) AND (periodont* OR Parodontiit) AND (cost OR expenditure OR spending OR revenue OR reimbursement OR Kulu) AND (health accounts OR ministry of health OR national OR federal OR insurance OR registry OR register OR database OR data OR dataset OR pubmed OR survey OR interview OR panel OR Sotsiaalministeerium OR Eesti Haigekassa) | Estonian | Parodontiit | Kulu | Sotsiaalministeerium | Eesti Haigekassa |
| Eswatini | (Eswatini) AND (periodont* OR Periodontitis) AND (cost OR expenditure OR spending OR revenue OR reimbursement OR Expenditure) AND (health accounts OR ministry of health OR national OR federal OR insurance OR registry OR register OR database OR data OR dataset OR pubmed OR survey OR interview OR panel OR Ministry of Health OR Insurance) | Swati, English | Periodontitis | Expenditure | Ministry of Health | Insurance |
| Ethiopia | (Ethiopia) AND (periodont* OR Periodontitis) AND (cost OR expenditure OR spending OR revenue OR reimbursement OR ወጪ (Wechi)) AND (health accounts OR ministry of health OR national OR federal OR insurance OR registry OR register OR database OR data OR dataset OR pubmed OR survey OR interview OR panel OR የጤና ሚኒስቴር OR Insurance) | Amharic | Periodontitis | ወጪ (Wechi) | የጤና ሚኒስቴር | Insurance |
| Fiji | (Fiji) AND (periodont* OR Periodontitis) AND (cost OR expenditure OR spending OR revenue OR reimbursement OR Expenditure) AND (health accounts OR ministry of health OR national OR federal OR insurance OR registry OR register OR database OR data OR dataset OR pubmed OR survey OR interview OR panel OR Ministry of Health and Medical Services OR Insurance) | English, Fijian, Hindi | Periodontitis | Expenditure | Ministry of Health and Medical Services | Insurance |
| Finland | (Finland) AND (periodont* OR Parodontiitti) AND (cost OR expenditure OR spending OR revenue OR reimbursement OR Menot / Utgifter) AND (health accounts OR ministry of health OR national OR federal OR insurance OR registry OR register OR database OR data OR dataset OR pubmed OR survey OR interview OR panel OR Sosiaali- ja terveysministeriö OR Kansaneläkelaitos (KELA)) | Finnish, Swedish | Parodontiitti | Menot / Utgifter | Sosiaali- ja terveysministeriö | Kansaneläkelaitos (KELA) |
| France | (France) AND (periodont* OR Parodontite) AND (cost OR expenditure OR spending OR revenue OR reimbursement OR Dépenses) AND (health accounts OR ministry of health OR national OR federal OR insurance OR registry OR register OR database OR data OR dataset OR pubmed OR survey OR interview OR panel OR Ministère des Solidarités et de la Santé OR Assurance Maladie) | French | Parodontite | Dépenses | Ministère des Solidarités et de la Santé | Assurance Maladie |
| Gabon | (Gabon) AND (periodont* OR Parodontite) AND (cost OR expenditure OR spending OR revenue OR reimbursement OR Dépenses) AND (health accounts OR ministry of health OR national OR federal OR insurance OR registry OR register OR database OR data OR dataset OR pubmed OR survey OR interview OR panel OR Ministère de la Santé OR Insurance) | French | Parodontite | Dépenses | Ministère de la Santé | Insurance |
| Gambia | (Gambia) AND (periodont* OR Periodontitis) AND (cost OR expenditure OR spending OR revenue OR reimbursement OR Expenditure) AND (health accounts OR ministry of health OR national OR federal OR insurance OR registry OR register OR database OR data OR dataset OR pubmed OR survey OR interview OR panel OR Ministry of Health OR Insurance) | English | Periodontitis | Expenditure | Ministry of Health | Insurance |
| Georgia | (Georgia) AND (periodont* OR პერიოდონტიტი) AND (cost OR expenditure OR spending OR revenue OR reimbursement OR ხარჯი (Kharchi)) AND (health accounts OR ministry of health OR national OR federal OR insurance OR registry OR register OR database OR data OR dataset OR pubmed OR survey OR interview OR panel OR საქართველოს შრომის, ჯანმრთელობისა და სოციალური დაცვის სამინისტრო OR Insurance) | Georgian | პერიოდონტიტი | ხარჯი (Kharchi) | საქართველოს შრომის, ჯანმრთელობისა და სოციალური დაცვის სამინისტრო | Insurance |
| Germany | (Germany) AND (periodont* OR Parodontitis) AND (cost OR expenditure OR spending OR revenue OR reimbursement OR Ausgaben) AND (health accounts OR ministry of health OR national OR federal OR insurance OR registry OR register OR database OR data OR dataset OR pubmed OR survey OR interview OR panel OR Bundesministerium für Gesundheit OR Gesetzliche Krankenversicherung (GKV)) | German | Parodontitis | Ausgaben | Bundesministerium für Gesundheit | Gesetzliche Krankenversicherung (GKV) |
| Ghana | (Ghana) AND (periodont* OR Periodontitis) AND (cost OR expenditure OR spending OR revenue OR reimbursement OR Expenditure) AND (health accounts OR ministry of health OR national OR federal OR insurance OR registry OR register OR database OR data OR dataset OR pubmed OR survey OR interview OR panel OR Ministry of Health OR National Health Insurance Scheme (NHIS)) | English | Periodontitis | Expenditure | Ministry of Health | National Health Insurance Scheme (NHIS) |
| Greece | (Greece) AND (periodont* OR Περιοδοντίτιδα) AND (cost OR expenditure OR spending OR revenue OR reimbursement OR Δαπάνη (Dapani)) AND (health accounts OR ministry of health OR national OR federal OR insurance OR registry OR register OR database OR data OR dataset OR pubmed OR survey OR interview OR panel OR Υπουργείο Υγείας OR Εθνικός Οργανισμός Παροχής Υπηρεσιών Υγείας (EOPYY)) | Greek | Περιοδοντίτιδα | Δαπάνη (Dapani) | Υπουργείο Υγείας | Εθνικός Οργανισμός Παροχής Υπηρεσιών Υγείας (EOPYY) |
| Grenada | (Grenada) AND (periodont* OR Periodontitis) AND (cost OR expenditure OR spending OR revenue OR reimbursement OR Expenditure) AND (health accounts OR ministry of health OR national OR federal OR insurance OR registry OR register OR database OR data OR dataset OR pubmed OR survey OR interview OR panel OR Ministry of Health, Wellness & Religious Affairs OR Insurance) | English | Periodontitis | Expenditure | Ministry of Health, Wellness & Religious Affairs | Insurance |
| Guatemala | (Guatemala) AND (periodont* OR Periodontitis) AND (cost OR expenditure OR spending OR revenue OR reimbursement OR Gasto) AND (health accounts OR ministry of health OR national OR federal OR insurance OR registry OR register OR database OR data OR dataset OR pubmed OR survey OR interview OR panel OR Ministerio de Salud Pública y Asistencia Social OR Instituto Guatemalteco de Seguridad Social) | Spanish | Periodontitis | Gasto | Ministerio de Salud Pública y Asistencia Social | Instituto Guatemalteco de Seguridad Social |
| Guinea | (Guinea) AND (periodont* OR Parodontite) AND (cost OR expenditure OR spending OR revenue OR reimbursement OR Dépenses) AND (health accounts OR ministry of health OR national OR federal OR insurance OR registry OR register OR database OR data OR dataset OR pubmed OR survey OR interview OR panel OR Ministère de la Santé OR Insurance) | French | Parodontite | Dépenses | Ministère de la Santé | Insurance |
| Guinea-Bissau | (Guinea-Bissau) AND (periodont* OR Parodontite) AND (cost OR expenditure OR spending OR revenue OR reimbursement OR Despesa) AND (health accounts OR ministry of health OR national OR federal OR insurance OR registry OR register OR database OR data OR dataset OR pubmed OR survey OR interview OR panel OR Ministério da Saúde Pública OR Insurance) | Portuguese | Parodontite | Despesa | Ministério da Saúde Pública | Insurance |
| Guyana | (Guyana) AND (periodont* OR Periodontitis) AND (cost OR expenditure OR spending OR revenue OR reimbursement OR Expenditure) AND (health accounts OR ministry of health OR national OR federal OR insurance OR registry OR register OR database OR data OR dataset OR pubmed OR survey OR interview OR panel OR Ministry of Health OR Insurance) | English | Periodontitis | Expenditure | Ministry of Health | Insurance |
| Haiti | (Haiti) AND (periodont* OR Parodontit) AND (cost OR expenditure OR spending OR revenue OR reimbursement OR Dépenses / Depans) AND (health accounts OR ministry of health OR national OR federal OR insurance OR registry OR register OR database OR data OR dataset OR pubmed OR survey OR interview OR panel OR Ministère de la Santé Publique et de la Population OR Insurance) | French, Haitian Creole | Parodontit | Dépenses / Depans | Ministère de la Santé Publique et de la Population | Insurance |
| Honduras | (Honduras) AND (periodont* OR Periodontitis) AND (cost OR expenditure OR spending OR revenue OR reimbursement OR Gasto) AND (health accounts OR ministry of health OR national OR federal OR insurance OR registry OR register OR database OR data OR dataset OR pubmed OR survey OR interview OR panel OR Secretaría de Salud OR Instituto Hondureño de Seguridad Social) | Spanish | Periodontitis | Gasto | Secretaría de Salud | Instituto Hondureño de Seguridad Social |
| Hungary | (Hungary) AND (periodont* OR Parodontitis) AND (cost OR expenditure OR spending OR revenue OR reimbursement OR Kiadások) AND (health accounts OR ministry of health OR national OR federal OR insurance OR registry OR register OR database OR data OR dataset OR pubmed OR survey OR interview OR panel OR Emberi Erőforrások Minisztériuma OR Nemzeti Egészségbiztosítási Alapkezelő) | Hungarian | Parodontitis | Kiadások | Emberi Erőforrások Minisztériuma | Nemzeti Egészségbiztosítási Alapkezelő |
| Iceland | (Iceland) AND (periodont* OR Periodontitis) AND (cost OR expenditure OR spending OR revenue OR reimbursement OR Útgjöld) AND (health accounts OR ministry of health OR national OR federal OR insurance OR registry OR register OR database OR data OR dataset OR pubmed OR survey OR interview OR panel OR Heilbrigðisráðuneytið OR Sjúkratryggingar Íslands) | Icelandic | Periodontitis | Útgjöld | Heilbrigðisráðuneytið | Sjúkratryggingar Íslands |
| India | (India) AND (periodont* OR पीरियडोंटाइटिस) AND (cost OR expenditure OR spending OR revenue OR reimbursement OR व्यय (Vyay) / Expenditure) AND (health accounts OR ministry of health OR national OR federal OR insurance OR registry OR register OR database OR data OR dataset OR pubmed OR survey OR interview OR panel OR Ministry of Health and Family Welfare OR Employees' State Insurance Scheme) | Hindi, English | पीरियडोंटाइटिस | व्यय (Vyay) / Expenditure | Ministry of Health and Family Welfare | Employees' State Insurance Scheme |
| Indonesia | (Indonesia) AND (periodont* OR Periodontitis) AND (cost OR expenditure OR spending OR revenue OR reimbursement OR Pengeluaran) AND (health accounts OR ministry of health OR national OR federal OR insurance OR registry OR register OR database OR data OR dataset OR pubmed OR survey OR interview OR panel OR Kementerian Kesehatan OR Badan Penyelenggara Jaminan Sosial Kesehatan) | Indonesian | Periodontitis | Pengeluaran | Kementerian Kesehatan | Badan Penyelenggara Jaminan Sosial Kesehatan |
| Iran (Islamic Republic of) | (Iran (Islamic Republic of)) AND (periodont* OR پریودنتیت) AND (cost OR expenditure OR spending OR revenue OR reimbursement OR هزینه‌ها (Hazineha)) AND (health accounts OR ministry of health OR national OR federal OR insurance OR registry OR register OR database OR data OR dataset OR pubmed OR survey OR interview OR panel OR وزارت بهداشت، درمان و آموزش پزشکی OR Insurance) | Persian | پریودنتیت | هزینه‌ها (Hazineha) | وزارت بهداشت، درمان و آموزش پزشکی | Insurance |
| Iraq | (Iraq) AND (periodont* OR التهاب دواعم السن) AND (cost OR expenditure OR spending OR revenue OR reimbursement OR النفقات (Al-Nafaqat) / خەرج (Kharj)) AND (health accounts OR ministry of health OR national OR federal OR insurance OR registry OR register OR database OR data OR dataset OR pubmed OR survey OR interview OR panel OR وزارة الصحة OR Insurance) | Arabic, Kurdish | التهاب دواعم السن | النفقات (Al-Nafaqat) / خەرج (Kharj) | وزارة الصحة | Insurance |
| Ireland | (Ireland) AND (periodont* OR Periodontitis) AND (cost OR expenditure OR spending OR revenue OR reimbursement OR Caiteachas / Expenditure) AND (health accounts OR ministry of health OR national OR federal OR insurance OR registry OR register OR database OR data OR dataset OR pubmed OR survey OR interview OR panel OR Department of Health OR Health Service Executive (HSE)) | Irish, English | Periodontitis | Caiteachas / Expenditure | Department of Health | Health Service Executive (HSE) |
| Israel | (Israel) AND (periodont* OR דלקת חניכיים) AND (cost OR expenditure OR spending OR revenue OR reimbursement OR הוצאה (Hotza'a)) AND (health accounts OR ministry of health OR national OR federal OR insurance OR registry OR register OR database OR data OR dataset OR pubmed OR survey OR interview OR panel OR משרד הבריאות OR קופת חולים (Kupat Holim)) | Hebrew | דלקת חניכיים | הוצאה (Hotza'a) | משרד הבריאות | קופת חולים (Kupat Holim) |
| Italy | (Italy) AND (periodont* OR Parodontite) AND (cost OR expenditure OR spending OR revenue OR reimbursement OR Spesa) AND (health accounts OR ministry of health OR national OR federal OR insurance OR registry OR register OR database OR data OR dataset OR pubmed OR survey OR interview OR panel OR Ministero della Salute OR Servizio Sanitario Nazionale (SSN)) | Italian | Parodontite | Spesa | Ministero della Salute | Servizio Sanitario Nazionale (SSN) |
| Jamaica | (Jamaica) AND (periodont* OR Periodontitis) AND (cost OR expenditure OR spending OR revenue OR reimbursement OR Expenditure) AND (health accounts OR ministry of health OR national OR federal OR insurance OR registry OR register OR database OR data OR dataset OR pubmed OR survey OR interview OR panel OR Ministry of Health and Wellness OR Insurance) | English | Periodontitis | Expenditure | Ministry of Health and Wellness | Insurance |
| Japan | (Japan) AND (periodont* OR 歯周炎) AND (cost OR expenditure OR spending OR revenue OR reimbursement OR 支出 (Shishutsu)) AND (health accounts OR ministry of health OR national OR federal OR insurance OR registry OR register OR database OR data OR dataset OR pubmed OR survey OR interview OR panel OR 厚生労働省 OR 国民健康保険 (Kokumin Kenkō Hoken)) | Japanese | 歯周炎 | 支出 (Shishutsu) | 厚生労働省 | 国民健康保険 (Kokumin Kenkō Hoken) |
| Jordan | (Jordan) AND (periodont* OR التهاب دواعم السن) AND (cost OR expenditure OR spending OR revenue OR reimbursement OR النفقات (Al-Nafaqat)) AND (health accounts OR ministry of health OR national OR federal OR insurance OR registry OR register OR database OR data OR dataset OR pubmed OR survey OR interview OR panel OR وزارة الصحة OR Insurance) | Arabic | التهاب دواعم السن | النفقات (Al-Nafaqat) | وزارة الصحة | Insurance |
| Kazakhstan | (Kazakhstan) AND (periodont* OR Периодонтит) AND (cost OR expenditure OR spending OR revenue OR reimbursement OR Шығындар (Shyǵyndar) / Расходы) AND (health accounts OR ministry of health OR national OR federal OR insurance OR registry OR register OR database OR data OR dataset OR pubmed OR survey OR interview OR panel OR Қазақстан Республикасының Денсаулық сақтау министрлігі OR Insurance) | Kazakh, Russian | Периодонтит | Шығындар (Shyǵyndar) / Расходы | Қазақстан Республикасының Денсаулық сақтау министрлігі | Insurance |
| Kenya | (Kenya) AND (periodont* OR Periodontitis) AND (cost OR expenditure OR spending OR revenue OR reimbursement OR Expenditure / Matumizi) AND (health accounts OR ministry of health OR national OR federal OR insurance OR registry OR register OR database OR data OR dataset OR pubmed OR survey OR interview OR panel OR Ministry of Health OR National Health Insurance Fund (NHIF)) | English, Swahili | Periodontitis | Expenditure / Matumizi | Ministry of Health | National Health Insurance Fund (NHIF) |
| Kiribati | (Kiribati) AND (periodont* OR Periodontitis) AND (cost OR expenditure OR spending OR revenue OR reimbursement OR Expenditure / Rawa) AND (health accounts OR ministry of health OR national OR federal OR insurance OR registry OR register OR database OR data OR dataset OR pubmed OR survey OR interview OR panel OR Ministry of Health and Medical Services OR Insurance) | English, Gilbertese | Periodontitis | Expenditure / Rawa | Ministry of Health and Medical Services | Insurance |
| Kuwait | (Kuwait) AND (periodont* OR التهاب دواعم السن) AND (cost OR expenditure OR spending OR revenue OR reimbursement OR النفقات (Al-Nafaqat)) AND (health accounts OR ministry of health OR national OR federal OR insurance OR registry OR register OR database OR data OR dataset OR pubmed OR survey OR interview OR panel OR وزارة الصحة OR Insurance) | Arabic | التهاب دواعم السن | النفقات (Al-Nafaqat) | وزارة الصحة | Insurance |
| Kyrgyzstan | (Kyrgyzstan) AND (periodont* OR Периодонтит) AND (cost OR expenditure OR spending OR revenue OR reimbursement OR Чыгымдар (Chygymdar) / Расходы) AND (health accounts OR ministry of health OR national OR federal OR insurance OR registry OR register OR database OR data OR dataset OR pubmed OR survey OR interview OR panel OR Кыргыз Республикасынын Саламаттыкты сактоо министрлиги OR Insurance) | Kyrgyz, Russian | Периодонтит | Чыгымдар (Chygymdar) / Расходы | Кыргыз Республикасынын Саламаттыкты сактоо министрлиги | Insurance |
| Lao People's Democratic Republic | (Lao People's Democratic Republic) AND (periodont* OR ปริทันต์อักเสบ) AND (cost OR expenditure OR spending OR revenue OR reimbursement OR ຄ່າໃຊ້ຈ່າຍ (Kha Chai Chai)) AND (health accounts OR ministry of health OR national OR federal OR insurance OR registry OR register OR database OR data OR dataset OR pubmed OR survey OR interview OR panel OR ກະຊວງສາທາລະນະສຸກ OR Insurance) | Lao | ปริทันต์อักเสบ | ຄ່າໃຊ້ຈ່າຍ (Kha Chai Chai) | ກະຊວງສາທາລະນະສຸກ | Insurance |
| Latvia | (Latvia) AND (periodont* OR Periodontīts) AND (cost OR expenditure OR spending OR revenue OR reimbursement OR Izdevumi) AND (health accounts OR ministry of health OR national OR federal OR insurance OR registry OR register OR database OR data OR dataset OR pubmed OR survey OR interview OR panel OR Veselības ministrija OR Insurance) | Latvian | Periodontīts | Izdevumi | Veselības ministrija | Insurance |
| Lebanon | (Lebanon) AND (periodont* OR التهاب دواعم السن) AND (cost OR expenditure OR spending OR revenue OR reimbursement OR النفقات (Al-Nafaqat)) AND (health accounts OR ministry of health OR national OR federal OR insurance OR registry OR register OR database OR data OR dataset OR pubmed OR survey OR interview OR panel OR وزارة الصحة العامة OR Insurance) | Arabic | التهاب دواعم السن | النفقات (Al-Nafaqat) | وزارة الصحة العامة | Insurance |
| Lesotho | (Lesotho) AND (periodont* OR Periodontitis) AND (cost OR expenditure OR spending OR revenue OR reimbursement OR Expenditure / Lichelete) AND (health accounts OR ministry of health OR national OR federal OR insurance OR registry OR register OR database OR data OR dataset OR pubmed OR survey OR interview OR panel OR Ministry of Health OR Insurance) | English, Sesotho | Periodontitis | Expenditure / Lichelete | Ministry of Health | Insurance |
| Liberia | (Liberia) AND (periodont* OR Periodontitis) AND (cost OR expenditure OR spending OR revenue OR reimbursement OR Expenditure) AND (health accounts OR ministry of health OR national OR federal OR insurance OR registry OR register OR database OR data OR dataset OR pubmed OR survey OR interview OR panel OR Ministry of Health OR Insurance) | English | Periodontitis | Expenditure | Ministry of Health | Insurance |
| Libya | (Libya) AND (periodont* OR التهاب دواعم السن) AND (cost OR expenditure OR spending OR revenue OR reimbursement OR النفقات (Al-Nafaqat)) AND (health accounts OR ministry of health OR national OR federal OR insurance OR registry OR register OR database OR data OR dataset OR pubmed OR survey OR interview OR panel OR وزارة الصحة OR Insurance) | Arabic | التهاب دواعم السن | النفقات (Al-Nafaqat) | وزارة الصحة | Insurance |
| Lithuania | (Lithuania) AND (periodont* OR Periodontitas) AND (cost OR expenditure OR spending OR revenue OR reimbursement OR Išlaidos) AND (health accounts OR ministry of health OR national OR federal OR insurance OR registry OR register OR database OR data OR dataset OR pubmed OR survey OR interview OR panel OR Sveikatos apsaugos ministerija OR Insurance) | Lithuanian | Periodontitas | Išlaidos | Sveikatos apsaugos ministerija | Insurance |
| Luxembourg | (Luxembourg) AND (periodont* OR Parodontite) AND (cost OR expenditure OR spending OR revenue OR reimbursement OR Ausgabe / Dépenses / Ausgaben) AND (health accounts OR ministry of health OR national OR federal OR insurance OR registry OR register OR database OR data OR dataset OR pubmed OR survey OR interview OR panel OR Ministère de la Santé OR Caisse Nationale de Santé (CNS)) | Luxembourgish, French, German | Parodontite | Ausgabe / Dépenses / Ausgaben | Ministère de la Santé | Caisse Nationale de Santé (CNS) |
| Madagascar | (Madagascar) AND (periodont* OR Periodontite) AND (cost OR expenditure OR spending OR revenue OR reimbursement OR Fandaniana / Dépenses) AND (health accounts OR ministry of health OR national OR federal OR insurance OR registry OR register OR database OR data OR dataset OR pubmed OR survey OR interview OR panel OR Ministère de la Santé Publique OR Insurance) | Malagasy, French | Periodontite | Fandaniana / Dépenses | Ministère de la Santé Publique | Insurance |
| Malawi | (Malawi) AND (periodont* OR Periodontitis) AND (cost OR expenditure OR spending OR revenue OR reimbursement OR Expenditure / Ndalama) AND (health accounts OR ministry of health OR national OR federal OR insurance OR registry OR register OR database OR data OR dataset OR pubmed OR survey OR interview OR panel OR Ministry of Health OR Insurance) | English, Chichewa | Periodontitis | Expenditure / Ndalama | Ministry of Health | Insurance |
| Malaysia | (Malaysia) AND (periodont* OR Periodontitis) AND (cost OR expenditure OR spending OR revenue OR reimbursement OR Perbelanjaan) AND (health accounts OR ministry of health OR national OR federal OR insurance OR registry OR register OR database OR data OR dataset OR pubmed OR survey OR interview OR panel OR Kementerian Kesihatan Malaysia OR Perkhidmatan Kesihatan Awam) | Malay | Periodontitis | Perbelanjaan | Kementerian Kesihatan Malaysia | Perkhidmatan Kesihatan Awam |
| Maldives | (Maldives) AND (periodont* OR Periodontitis) AND (cost OR expenditure OR spending OR revenue OR reimbursement OR ކޮންމެ އެކަލް (Konme Ekal)) AND (health accounts OR ministry of health OR national OR federal OR insurance OR registry OR register OR database OR data OR dataset OR pubmed OR survey OR interview OR panel OR މިނިސްޓްރީ އޮފް ހެލްތް OR Insurance) | Dhivehi | Periodontitis | ކޮންމެ އެކަލް (Konme Ekal) | މިނިސްޓްރީ އޮފް ހެލްތް | Insurance |
| Mali | (Mali) AND (periodont* OR Parodontite) AND (cost OR expenditure OR spending OR revenue OR reimbursement OR Dépenses) AND (health accounts OR ministry of health OR national OR federal OR insurance OR registry OR register OR database OR data OR dataset OR pubmed OR survey OR interview OR panel OR Ministère de la Santé et de l'Hygiène Publique OR Insurance) | French | Parodontite | Dépenses | Ministère de la Santé et de l'Hygiène Publique | Insurance |
| Malta | (Malta) AND (periodont* OR Periodontitis) AND (cost OR expenditure OR spending OR revenue OR reimbursement OR Nefqa / Expenditure) AND (health accounts OR ministry of health OR national OR federal OR insurance OR registry OR register OR database OR data OR dataset OR pubmed OR survey OR interview OR panel OR Ministeru għas-Saħħa OR Insurance) | Maltese, English | Periodontitis | Nefqa / Expenditure | Ministeru għas-Saħħa | Insurance |
| Marshall Islands | (Marshall Islands) AND (periodont* OR Periodontitis) AND (cost OR expenditure OR spending OR revenue OR reimbursement OR Expenditure / Ekadak) AND (health accounts OR ministry of health OR national OR federal OR insurance OR registry OR register OR database OR data OR dataset OR pubmed OR survey OR interview OR panel OR Ministry of Health and Human Services OR Insurance) | English, Marshallese | Periodontitis | Expenditure / Ekadak | Ministry of Health and Human Services | Insurance |
| Mauritania | (Mauritania) AND (periodont* OR التهاب دواعم السن) AND (cost OR expenditure OR spending OR revenue OR reimbursement OR النفقات (Al-Nafaqat)) AND (health accounts OR ministry of health OR national OR federal OR insurance OR registry OR register OR database OR data OR dataset OR pubmed OR survey OR interview OR panel OR وزارة الصحة OR Insurance) | Arabic | التهاب دواعم السن | النفقات (Al-Nafaqat) | وزارة الصحة | Insurance |
| Mauritius | (Mauritius) AND (periodont* OR Periodontitis) AND (cost OR expenditure OR spending OR revenue OR reimbursement OR Expenditure) AND (health accounts OR ministry of health OR national OR federal OR insurance OR registry OR register OR database OR data OR dataset OR pubmed OR survey OR interview OR panel OR Ministry of Health and Wellness OR Insurance) | English | Periodontitis | Expenditure | Ministry of Health and Wellness | Insurance |
| Mexico | (Mexico) AND (periodont* OR Periodontitis) AND (cost OR expenditure OR spending OR revenue OR reimbursement OR Gasto) AND (health accounts OR ministry of health OR national OR federal OR insurance OR registry OR register OR database OR data OR dataset OR pubmed OR survey OR interview OR panel OR Secretaría de Salud OR Instituto Mexicano del Seguro Social (IMSS)) | Spanish | Periodontitis | Gasto | Secretaría de Salud | Instituto Mexicano del Seguro Social (IMSS) |
| Micronesia (Federated States of) | (Micronesia (Federated States of)) AND (periodont* OR Periodontitis) AND (cost OR expenditure OR spending OR revenue OR reimbursement OR Expenditure) AND (health accounts OR ministry of health OR national OR federal OR insurance OR registry OR register OR database OR data OR dataset OR pubmed OR survey OR interview OR panel OR Department of Health and Social Affairs OR Insurance) | English | Periodontitis | Expenditure | Department of Health and Social Affairs | Insurance |
| Monaco | (Monaco) AND (periodont* OR Parodontite) AND (cost OR expenditure OR spending OR revenue OR reimbursement OR Dépenses) AND (health accounts OR ministry of health OR national OR federal OR insurance OR registry OR register OR database OR data OR dataset OR pubmed OR survey OR interview OR panel OR Département des Affaires Sociales et de la Santé OR Insurance) | French | Parodontite | Dépenses | Département des Affaires Sociales et de la Santé | Insurance |
| Mongolia | (Mongolia) AND (periodont* OR Периодонтит) AND (cost OR expenditure OR spending OR revenue OR reimbursement OR Зардал (Zardal)) AND (health accounts OR ministry of health OR national OR federal OR insurance OR registry OR register OR database OR data OR dataset OR pubmed OR survey OR interview OR panel OR Эрүүл мэндийн яам OR Insurance) | Mongolian | Периодонтит | Зардал (Zardal) | Эрүүл мэндийн яам | Insurance |
| Montenegro | (Montenegro) AND (periodont* OR Parodontitis) AND (cost OR expenditure OR spending OR revenue OR reimbursement OR Trošak) AND (health accounts OR ministry of health OR national OR federal OR insurance OR registry OR register OR database OR data OR dataset OR pubmed OR survey OR interview OR panel OR Ministarstvo zdravlja OR Insurance) | Montenegrin | Parodontitis | Trošak | Ministarstvo zdravlja | Insurance |
| Morocco | (Morocco) AND (periodont* OR التهاب دواعم السن) AND (cost OR expenditure OR spending OR revenue OR reimbursement OR النفقات (Al-Nafaqat)) AND (health accounts OR ministry of health OR national OR federal OR insurance OR registry OR register OR database OR data OR dataset OR pubmed OR survey OR interview OR panel OR وزارة الصحة OR Insurance) | Arabic | التهاب دواعم السن | النفقات (Al-Nafaqat) | وزارة الصحة | Insurance |
| Mozambique | (Mozambique) AND (periodont* OR Periodontite) AND (cost OR expenditure OR spending OR revenue OR reimbursement OR Despesa) AND (health accounts OR ministry of health OR national OR federal OR insurance OR registry OR register OR database OR data OR dataset OR pubmed OR survey OR interview OR panel OR Ministério da Saúde OR Insurance) | Portuguese | Periodontite | Despesa | Ministério da Saúde | Insurance |
| Myanmar | (Myanmar) AND (periodont* OR Periodontitis) AND (cost OR expenditure OR spending OR revenue OR reimbursement OR ကုန်ကျစရိတ် (Konekyay Chayat)) AND (health accounts OR ministry of health OR national OR federal OR insurance OR registry OR register OR database OR data OR dataset OR pubmed OR survey OR interview OR panel OR ကျန်းမာရေးနှင့် အားကစားဝန်ကြီးဌာန OR Insurance) | Burmese | Periodontitis | ကုန်ကျစရိတ် (Konekyay Chayat) | ကျန်းမာရေးနှင့် အားကစားဝန်ကြီးဌာန | Insurance |
| Namibia | (Namibia) AND (periodont* OR Periodontitis) AND (cost OR expenditure OR spending OR revenue OR reimbursement OR Expenditure) AND (health accounts OR ministry of health OR national OR federal OR insurance OR registry OR register OR database OR data OR dataset OR pubmed OR survey OR interview OR panel OR Ministry of Health and Social Services OR Insurance) | English | Periodontitis | Expenditure | Ministry of Health and Social Services | Insurance |
| Nauru | (Nauru) AND (periodont* OR Periodontitis) AND (cost OR expenditure OR spending OR revenue OR reimbursement OR Expenditure / Bwiema) AND (health accounts OR ministry of health OR national OR federal OR insurance OR registry OR register OR database OR data OR dataset OR pubmed OR survey OR interview OR panel OR Department of Health OR Insurance) | English, Nauruan | Periodontitis | Expenditure / Bwiema | Department of Health | Insurance |
| Nepal | (Nepal) AND (periodont* OR पीरियडोंटाइटिस) AND (cost OR expenditure OR spending OR revenue OR reimbursement OR खर्च (Kharcha)) AND (health accounts OR ministry of health OR national OR federal OR insurance OR registry OR register OR database OR data OR dataset OR pubmed OR survey OR interview OR panel OR स्वास्थ्य तथा जनसंख्या मन्त्रालय OR Insurance) | Nepali | पीरियडोंटाइटिस | खर्च (Kharcha) | स्वास्थ्य तथा जनसंख्या मन्त्रालय | Insurance |
| Netherlands | (Netherlands) AND (periodont* OR Parodontitis) AND (cost OR expenditure OR spending OR revenue OR reimbursement OR Uitgaven) AND (health accounts OR ministry of health OR national OR federal OR insurance OR registry OR register OR database OR data OR dataset OR pubmed OR survey OR interview OR panel OR Ministerie van Volksgezondheid, Welzijn en Sport OR Zorgverzekeringswet (Zvw)) | Dutch | Parodontitis | Uitgaven | Ministerie van Volksgezondheid, Welzijn en Sport | Zorgverzekeringswet (Zvw) |
| New Zealand | (New Zealand) AND (periodont* OR Periodontitis) AND (cost OR expenditure OR spending OR revenue OR reimbursement OR Expenditure / Whakapaunga) AND (health accounts OR ministry of health OR national OR federal OR insurance OR registry OR register OR database OR data OR dataset OR pubmed OR survey OR interview OR panel OR Ministry of Health OR Accident Compensation Corporation (ACC)) | English, Māori | Periodontitis | Expenditure / Whakapaunga | Ministry of Health | Accident Compensation Corporation (ACC) |
| Nicaragua | (Nicaragua) AND (periodont* OR Periodontitis) AND (cost OR expenditure OR spending OR revenue OR reimbursement OR Gasto) AND (health accounts OR ministry of health OR national OR federal OR insurance OR registry OR register OR database OR data OR dataset OR pubmed OR survey OR interview OR panel OR Ministerio de Salud OR Insurance) | Spanish | Periodontitis | Gasto | Ministerio de Salud | Insurance |
| Niger | (Niger) AND (periodont* OR Parodontite) AND (cost OR expenditure OR spending OR revenue OR reimbursement OR Dépenses) AND (health accounts OR ministry of health OR national OR federal OR insurance OR registry OR register OR database OR data OR dataset OR pubmed OR survey OR interview OR panel OR Ministère de la Santé Publique OR Insurance) | French | Parodontite | Dépenses | Ministère de la Santé Publique | Insurance |
| Nigeria | (Nigeria) AND (periodont* OR Periodontitis) AND (cost OR expenditure OR spending OR revenue OR reimbursement OR Expenditure) AND (health accounts OR ministry of health OR national OR federal OR insurance OR registry OR register OR database OR data OR dataset OR pubmed OR survey OR interview OR panel OR Federal Ministry of Health OR National Health Insurance Scheme (NHIS)) | English | Periodontitis | Expenditure | Federal Ministry of Health | National Health Insurance Scheme (NHIS) |
| Niue | (Niue) AND (periodont* OR Periodontitis) AND (cost OR expenditure OR spending OR revenue OR reimbursement OR Expenditure / Holoaga) AND (health accounts OR ministry of health OR national OR federal OR insurance OR registry OR register OR database OR data OR dataset OR pubmed OR survey OR interview OR panel OR Department of Health OR Insurance) | English, Niuean | Periodontitis | Expenditure / Holoaga | Department of Health | Insurance |
| North Macedonia | (North Macedonia) AND (periodont* OR Пародонтит) AND (cost OR expenditure OR spending OR revenue OR reimbursement OR Трошок (Trošok)) AND (health accounts OR ministry of health OR national OR federal OR insurance OR registry OR register OR database OR data OR dataset OR pubmed OR survey OR interview OR panel OR Министерство за здравство OR Insurance) | Macedonian | Пародонтит | Трошок (Trošok) | Министерство за здравство | Insurance |
| Norway | (Norway) AND (periodont* OR Periodontitt) AND (cost OR expenditure OR spending OR revenue OR reimbursement OR Utgift) AND (health accounts OR ministry of health OR national OR federal OR insurance OR registry OR register OR database OR data OR dataset OR pubmed OR survey OR interview OR panel OR Helse- og omsorgsdepartementet OR Folketrygden) | Norwegian | Periodontitt | Utgift | Helse- og omsorgsdepartementet | Folketrygden |
| Oman | (Oman) AND (periodont* OR التهاب دواعم السن) AND (cost OR expenditure OR spending OR revenue OR reimbursement OR النفقات (Al-Nafaqat)) AND (health accounts OR ministry of health OR national OR federal OR insurance OR registry OR register OR database OR data OR dataset OR pubmed OR survey OR interview OR panel OR وزارة الصحة OR Insurance) | Arabic | التهاب دواعم السن | النفقات (Al-Nafaqat) | وزارة الصحة | Insurance |
| Pakistan | (Pakistan) AND (periodont* OR پیریڈونٹائٹس) AND (cost OR expenditure OR spending OR revenue OR reimbursement OR اخراجات (Akhrajat) / Expenditure) AND (health accounts OR ministry of health OR national OR federal OR insurance OR registry OR register OR database OR data OR dataset OR pubmed OR survey OR interview OR panel OR وزارت صحت، خدمات ضابطہ کاری OR Insurance) | Urdu, English | پیریڈونٹائٹس | اخراجات (Akhrajat) / Expenditure | وزارت صحت، خدمات ضابطہ کاری | Insurance |
| Palau | (Palau) AND (periodont* OR Periodontitis) AND (cost OR expenditure OR spending OR revenue OR reimbursement OR Expenditure / Tial) AND (health accounts OR ministry of health OR national OR federal OR insurance OR registry OR register OR database OR data OR dataset OR pubmed OR survey OR interview OR panel OR Ministry of Health and Human Services OR Insurance) | English, Palauan | Periodontitis | Expenditure / Tial | Ministry of Health and Human Services | Insurance |
| Panama | (Panama) AND (periodont* OR Periodontitis) AND (cost OR expenditure OR spending OR revenue OR reimbursement OR Gasto) AND (health accounts OR ministry of health OR national OR federal OR insurance OR registry OR register OR database OR data OR dataset OR pubmed OR survey OR interview OR panel OR Ministerio de Salud OR Insurance) | Spanish | Periodontitis | Gasto | Ministerio de Salud | Insurance |
| Papua New Guinea | (Papua New Guinea) AND (periodont* OR Periodontitis) AND (cost OR expenditure OR spending OR revenue OR reimbursement OR Expenditure / Kostim) AND (health accounts OR ministry of health OR national OR federal OR insurance OR registry OR register OR database OR data OR dataset OR pubmed OR survey OR interview OR panel OR National Department of Health OR Insurance) | English, Tok Pisin, Hiri Motu | Periodontitis | Expenditure / Kostim | National Department of Health | Insurance |
| Paraguay | (Paraguay) AND (periodont* OR Periodontitis) AND (cost OR expenditure OR spending OR revenue OR reimbursement OR Gasto / Ñembosarái) AND (health accounts OR ministry of health OR national OR federal OR insurance OR registry OR register OR database OR data OR dataset OR pubmed OR survey OR interview OR panel OR Ministerio de Salud Pública y Bienestar Social OR Insurance) | Spanish, Guarani | Periodontitis | Gasto / Ñembosarái | Ministerio de Salud Pública y Bienestar Social | Insurance |
| Peru | (Peru) AND (periodont* OR Periodontitis) AND (cost OR expenditure OR spending OR revenue OR reimbursement OR Gasto) AND (health accounts OR ministry of health OR national OR federal OR insurance OR registry OR register OR database OR data OR dataset OR pubmed OR survey OR interview OR panel OR Ministerio de Salud OR Seguro Integral de Salud (SIS)) | Spanish | Periodontitis | Gasto | Ministerio de Salud | Seguro Integral de Salud (SIS) |
| Philippines | (Philippines) AND (periodont* OR Periodontitis) AND (cost OR expenditure OR spending OR revenue OR reimbursement OR Gastos / Expenditure) AND (health accounts OR ministry of health OR national OR federal OR insurance OR registry OR register OR database OR data OR dataset OR pubmed OR survey OR interview OR panel OR Kagawaran ng Kalusugan (Department of Health) OR Philippine Health Insurance Corporation (PhilHealth)) | Filipino, English | Periodontitis | Gastos / Expenditure | Kagawaran ng Kalusugan (Department of Health) | Philippine Health Insurance Corporation (PhilHealth) |
| Poland | (Poland) AND (periodont* OR Periodontitis) AND (cost OR expenditure OR spending OR revenue OR reimbursement OR Wydatki) AND (health accounts OR ministry of health OR national OR federal OR insurance OR registry OR register OR database OR data OR dataset OR pubmed OR survey OR interview OR panel OR Ministerstwo Zdrowia OR Narodowy Fundusz Zdrowia (NFZ)) | Polish | Periodontitis | Wydatki | Ministerstwo Zdrowia | Narodowy Fundusz Zdrowia (NFZ) |
| Portugal | (Portugal) AND (periodont* OR Periodontite) AND (cost OR expenditure OR spending OR revenue OR reimbursement OR Despesa) AND (health accounts OR ministry of health OR national OR federal OR insurance OR registry OR register OR database OR data OR dataset OR pubmed OR survey OR interview OR panel OR Ministério da Saúde OR Serviço Nacional de Saúde (SNS)) | Portuguese | Periodontite | Despesa | Ministério da Saúde | Serviço Nacional de Saúde (SNS) |
| Qatar | (Qatar) AND (periodont* OR التهاب دواعم السن) AND (cost OR expenditure OR spending OR revenue OR reimbursement OR النفقات (Al-Nafaqat)) AND (health accounts OR ministry of health OR national OR federal OR insurance OR registry OR register OR database OR data OR dataset OR pubmed OR survey OR interview OR panel OR وزارة الصحة العامة (Ministry of Public Health) OR Insurance) | Arabic | التهاب دواعم السن | النفقات (Al-Nafaqat) | وزارة الصحة العامة (Ministry of Public Health) | Insurance |
| Republic of China (Taiwan) | (Republic of China (Taiwan)) AND (periodont* OR 牙周病) AND (cost OR expenditure OR spending OR revenue OR reimbursement OR 支出) AND (health accounts OR ministry of health OR national OR federal OR insurance OR registry OR register OR database OR data OR dataset OR pubmed OR survey OR interview OR panel OR 卫生福利部 OR National Health Insurance) | Chinese (Mandarin) | 牙周病 | 支出 | 卫生福利部 | National Health Insurance |
| Republic of Korea | (Republic of Korea) AND (periodont* OR 치주염) AND (cost OR expenditure OR spending OR revenue OR reimbursement OR 지출 (Jichul)) AND (health accounts OR ministry of health OR national OR federal OR insurance OR registry OR register OR database OR data OR dataset OR pubmed OR survey OR interview OR panel OR 보건복지부 (Ministry of Health and Welfare) OR 국민건강보험 (Kukmin Geongang Bohum)) | Korean | 치주염 | 지출 (Jichul) | 보건복지부 (Ministry of Health and Welfare) | 국민건강보험 (Kukmin Geongang Bohum) |
| Republic of Moldova | (Republic of Moldova) AND (periodont* OR Parodontită) AND (cost OR expenditure OR spending OR revenue OR reimbursement OR Cheltuieli) AND (health accounts OR ministry of health OR national OR federal OR insurance OR registry OR register OR database OR data OR dataset OR pubmed OR survey OR interview OR panel OR Ministerul Sănătății OR Insurance) | Romanian | Parodontită | Cheltuieli | Ministerul Sănătății | Insurance |
| Romania | (Romania) AND (periodont* OR Parodontită) AND (cost OR expenditure OR spending OR revenue OR reimbursement OR Cheltuieli) AND (health accounts OR ministry of health OR national OR federal OR insurance OR registry OR register OR database OR data OR dataset OR pubmed OR survey OR interview OR panel OR Ministerul Sănătății OR Casa Națională de Asigurări de Sănătate) | Romanian | Parodontită | Cheltuieli | Ministerul Sănătății | Casa Națională de Asigurări de Sănătate |
| Russian Federation | (Russian Federation) AND (periodont* OR Пародонтит) AND (cost OR expenditure OR spending OR revenue OR reimbursement OR Расходы (Raskhody)) AND (health accounts OR ministry of health OR national OR federal OR insurance OR registry OR register OR database OR data OR dataset OR pubmed OR survey OR interview OR panel OR Министерство здравоохранения Российской Федерации (Ministry of Health of the Russian Federation) OR Федеральный фонд обязательного медицинского страхования (FFOMS)) | Russian | Пародонтит | Расходы (Raskhody) | Министерство здравоохранения Российской Федерации (Ministry of Health of the Russian Federation) | Федеральный фонд обязательного медицинского страхования (FFOMS) |
| Rwanda | (Rwanda) AND (periodont* OR Periodontitis) AND (cost OR expenditure OR spending OR revenue OR reimbursement OR Amafaranga yakoreshejwe / Dépenses) AND (health accounts OR ministry of health OR national OR federal OR insurance OR registry OR register OR database OR data OR dataset OR pubmed OR survey OR interview OR panel OR Ministry of Health OR Insurance) | Kinyarwanda, French, English | Periodontitis | Amafaranga yakoreshejwe / Dépenses | Ministry of Health | Insurance |
| Saint Kitts and Nevis | (Saint Kitts and Nevis) AND (periodont* OR Periodontitis) AND (cost OR expenditure OR spending OR revenue OR reimbursement OR Expenditure) AND (health accounts OR ministry of health OR national OR federal OR insurance OR registry OR register OR database OR data OR dataset OR pubmed OR survey OR interview OR panel OR Ministry of Health OR Insurance) | English | Periodontitis | Expenditure | Ministry of Health | Insurance |
| Saint Lucia | (Saint Lucia) AND (periodont* OR Periodontitis) AND (cost OR expenditure OR spending OR revenue OR reimbursement OR Expenditure) AND (health accounts OR ministry of health OR national OR federal OR insurance OR registry OR register OR database OR data OR dataset OR pubmed OR survey OR interview OR panel OR Ministry of Health and Wellness OR Insurance) | English | Periodontitis | Expenditure | Ministry of Health and Wellness | Insurance |
| Saint Vincent and the Grenadines | (Saint Vincent and the Grenadines) AND (periodont* OR Periodontitis) AND (cost OR expenditure OR spending OR revenue OR reimbursement OR Expenditure) AND (health accounts OR ministry of health OR national OR federal OR insurance OR registry OR register OR database OR data OR dataset OR pubmed OR survey OR interview OR panel OR Ministry of Health, Wellness and the Environment OR Insurance) | English | Periodontitis | Expenditure | Ministry of Health, Wellness and the Environment | Insurance |
| Samoa | (Samoa) AND (periodont* OR Periodontitis) AND (cost OR expenditure OR spending OR revenue OR reimbursement OR Expenditure / Faʻagaioiga) AND (health accounts OR ministry of health OR national OR federal OR insurance OR registry OR register OR database OR data OR dataset OR pubmed OR survey OR interview OR panel OR Ministry of Health OR Insurance) | English, Samoan | Periodontitis | Expenditure / Faʻagaioiga | Ministry of Health | Insurance |
| San Marino | (San Marino) AND (periodont* OR Parodontite) AND (cost OR expenditure OR spending OR revenue OR reimbursement OR Spesa) AND (health accounts OR ministry of health OR national OR federal OR insurance OR registry OR register OR database OR data OR dataset OR pubmed OR survey OR interview OR panel OR Segreteria di Stato per la Sanità e Sicurezza Sociale (State Secretariat for Health and Social Security) OR Insurance) | Italian | Parodontite | Spesa | Segreteria di Stato per la Sanità e Sicurezza Sociale (State Secretariat for Health and Social Security) | Insurance |
| Sao Tome and Principe | (Sao Tome and Principe) AND (periodont* OR Periodontite) AND (cost OR expenditure OR spending OR revenue OR reimbursement OR Despesa) AND (health accounts OR ministry of health OR national OR federal OR insurance OR registry OR register OR database OR data OR dataset OR pubmed OR survey OR interview OR panel OR Ministério da Saúde OR Insurance) | Portuguese | Periodontite | Despesa | Ministério da Saúde | Insurance |
| Saudi Arabia | (Saudi Arabia) AND (periodont* OR التهاب دواعم السن) AND (cost OR expenditure OR spending OR revenue OR reimbursement OR النفقات (Al-Nafaqat)) AND (health accounts OR ministry of health OR national OR federal OR insurance OR registry OR register OR database OR data OR dataset OR pubmed OR survey OR interview OR panel OR وزارة الصحة (Ministry of Health) OR Insurance) | Arabic | التهاب دواعم السن | النفقات (Al-Nafaqat) | وزارة الصحة (Ministry of Health) | Insurance |
| Senegal | (Senegal) AND (periodont* OR Parodontite) AND (cost OR expenditure OR spending OR revenue OR reimbursement OR Dépenses) AND (health accounts OR ministry of health OR national OR federal OR insurance OR registry OR register OR database OR data OR dataset OR pubmed OR survey OR interview OR panel OR Ministère de la Santé et de l'Action Sociale (Ministry of Health and Social Action) OR Insurance) | French | Parodontite | Dépenses | Ministère de la Santé et de l'Action Sociale (Ministry of Health and Social Action) | Insurance |
| Serbia | (Serbia) AND (periodont* OR Пародонтит) AND (cost OR expenditure OR spending OR revenue OR reimbursement OR Трошак (Trošak)) AND (health accounts OR ministry of health OR national OR federal OR insurance OR registry OR register OR database OR data OR dataset OR pubmed OR survey OR interview OR panel OR Министарство здравља (Ministarstvo zdravlja) OR Insurance) | Serbian | Пародонтит | Трошак (Trošak) | Министарство здравља (Ministarstvo zdravlja) | Insurance |
| Seychelles | (Seychelles) AND (periodont* OR Periodontitis) AND (cost OR expenditure OR spending OR revenue OR reimbursement OR Expenditure / Dépenses) AND (health accounts OR ministry of health OR national OR federal OR insurance OR registry OR register OR database OR data OR dataset OR pubmed OR survey OR interview OR panel OR Ministry of Health OR Insurance) | English, French, Seychellois Creole | Periodontitis | Expenditure / Dépenses | Ministry of Health | Insurance |
| Sierra Leone | (Sierra Leone) AND (periodont* OR Periodontitis) AND (cost OR expenditure OR spending OR revenue OR reimbursement OR Expenditure) AND (health accounts OR ministry of health OR national OR federal OR insurance OR registry OR register OR database OR data OR dataset OR pubmed OR survey OR interview OR panel OR Ministry of Health and Sanitation OR Insurance) | English | Periodontitis | Expenditure | Ministry of Health and Sanitation | Insurance |
| Singapore | (Singapore) AND (periodont* OR Periodontitis) AND (cost OR expenditure OR spending OR revenue OR reimbursement OR Expenditure / Perbelanjaan / செலவுகள் / 支出) AND (health accounts OR ministry of health OR national OR federal OR insurance OR registry OR register OR database OR data OR dataset OR pubmed OR survey OR interview OR panel OR Ministry of Health OR Medisave, Medishield Life) | English, Malay, Tamil, Chinese | Periodontitis | Expenditure / Perbelanjaan / செலவுகள் / 支出 | Ministry of Health | Medisave, Medishield Life |
| Slovakia | (Slovakia) AND (periodont* OR Parodontitída) AND (cost OR expenditure OR spending OR revenue OR reimbursement OR Výdavky) AND (health accounts OR ministry of health OR national OR federal OR insurance OR registry OR register OR database OR data OR dataset OR pubmed OR survey OR interview OR panel OR Ministerstvo zdravotníctva OR Všeobecná zdravotná poisťovňa) | Slovak | Parodontitída | Výdavky | Ministerstvo zdravotníctva | Všeobecná zdravotná poisťovňa |
| Slovenia | (Slovenia) AND (periodont* OR Parodontitis) AND (cost OR expenditure OR spending OR revenue OR reimbursement OR Stroški) AND (health accounts OR ministry of health OR national OR federal OR insurance OR registry OR register OR database OR data OR dataset OR pubmed OR survey OR interview OR panel OR Ministrstvo za zdravje OR Zavod za zdravstveno zavarovanje Slovenije) | Slovenian | Parodontitis | Stroški | Ministrstvo za zdravje | Zavod za zdravstveno zavarovanje Slovenije |
| Solomon Islands | (Solomon Islands) AND (periodont* OR Periodontitis) AND (cost OR expenditure OR spending OR revenue OR reimbursement OR Expenditure) AND (health accounts OR ministry of health OR national OR federal OR insurance OR registry OR register OR database OR data OR dataset OR pubmed OR survey OR interview OR panel OR Ministry of Health and Medical Services OR Insurance) | English | Periodontitis | Expenditure | Ministry of Health and Medical Services | Insurance |
| Somalia | (Somalia) AND (periodont* OR Periodontitis) AND (cost OR expenditure OR spending OR revenue OR reimbursement OR Kharashka / النفقات) AND (health accounts OR ministry of health OR national OR federal OR insurance OR registry OR register OR database OR data OR dataset OR pubmed OR survey OR interview OR panel OR Wasaaradda Caafimaadka (Ministry of Health) OR Insurance) | Somali, Arabic | Periodontitis | Kharashka / النفقات | Wasaaradda Caafimaadka (Ministry of Health) | Insurance |
| South Africa | (South Africa) AND (periodont* OR Periodontitis) AND (cost OR expenditure OR spending OR revenue OR reimbursement OR Expenditure / Uitgawe / Ukusetshenziswa) AND (health accounts OR ministry of health OR national OR federal OR insurance OR registry OR register OR database OR data OR dataset OR pubmed OR survey OR interview OR panel OR National Department of Health OR Insurance) | 11 Official Languages | Periodontitis | Expenditure / Uitgawe / Ukusetshenziswa | National Department of Health | Insurance |
| South Sudan | (South Sudan) AND (periodont* OR Periodontitis) AND (cost OR expenditure OR spending OR revenue OR reimbursement OR Expenditure) AND (health accounts OR ministry of health OR national OR federal OR insurance OR registry OR register OR database OR data OR dataset OR pubmed OR survey OR interview OR panel OR Ministry of Health OR Insurance) | English | Periodontitis | Expenditure | Ministry of Health | Insurance |
| Spain | (Spain) AND (periodont* OR Periodontitis) AND (cost OR expenditure OR spending OR revenue OR reimbursement OR Gasto) AND (health accounts OR ministry of health OR national OR federal OR insurance OR registry OR register OR database OR data OR dataset OR pubmed OR survey OR interview OR panel OR Ministerio de Sanidad OR Sistema Nacional de Salud) | Spanish | Periodontitis | Gasto | Ministerio de Sanidad | Sistema Nacional de Salud |
| Sri Lanka | (Sri Lanka) AND (periodont* OR පරිඩොන්ටයිටිස්) AND (cost OR expenditure OR spending OR revenue OR reimbursement OR වියදම් (Viyadam) / செலவுகள்) AND (health accounts OR ministry of health OR national OR federal OR insurance OR registry OR register OR database OR data OR dataset OR pubmed OR survey OR interview OR panel OR සෞඛ්‍ය අමාත්‍යාංශය (Saukya Amathyanshaya) / சுகாதார அமைச்சு (Sukādhāra Amaccu) (Ministry of Health) OR Insurance) | Sinhala, Tamil | පරිඩොන්ටයිටිස් | වියදම් (Viyadam) / செலவுகள் | සෞඛ්‍ය අමාත්‍යාංශය (Saukya Amathyanshaya) / சுகாதார அமைச்சு (Sukādhāra Amaccu) (Ministry of Health) | Insurance |
| Sudan | (Sudan) AND (periodont* OR التهاب دواعم السن) AND (cost OR expenditure OR spending OR revenue OR reimbursement OR النفقات (Al-Nafaqat) / Expenditure) AND (health accounts OR ministry of health OR national OR federal OR insurance OR registry OR register OR database OR data OR dataset OR pubmed OR survey OR interview OR panel OR وزارة الصحة الاتحادية (Federal Ministry of Health) OR Insurance) | Arabic, English | التهاب دواعم السن | النفقات (Al-Nafaqat) / Expenditure | وزارة الصحة الاتحادية (Federal Ministry of Health) | Insurance |
| Suriname | (Suriname) AND (periodont* OR Parodontitis) AND (cost OR expenditure OR spending OR revenue OR reimbursement OR Uitgaven) AND (health accounts OR ministry of health OR national OR federal OR insurance OR registry OR register OR database OR data OR dataset OR pubmed OR survey OR interview OR panel OR Ministerie van Volksgezondheid OR Insurance) | Dutch | Parodontitis | Uitgaven | Ministerie van Volksgezondheid | Insurance |
| Sweden | (Sweden) AND (periodont* OR Parodontit) AND (cost OR expenditure OR spending OR revenue OR reimbursement OR Utgifter) AND (health accounts OR ministry of health OR national OR federal OR insurance OR registry OR register OR database OR data OR dataset OR pubmed OR survey OR interview OR panel OR Socialdepartementet (Ministry of Health and Social Affairs) OR Försäkringskassan) | Swedish | Parodontit | Utgifter | Socialdepartementet (Ministry of Health and Social Affairs) | Försäkringskassan |
| Switzerland | (Switzerland) AND (periodont* OR Parodontite) AND (cost OR expenditure OR spending OR revenue OR reimbursement OR Ausgaben / Dépenses / Spese / Expensas) AND (health accounts OR ministry of health OR national OR federal OR insurance OR registry OR register OR database OR data OR dataset OR pubmed OR survey OR interview OR panel OR Bundesamt für Gesundheit (Office fédéral de la santé publique / Ufficio federale della sanità pubblica) (Federal Office of Public Health) OR Krankenversicherungsgesetz (KVG/LAMal)) | German, French, Italian, Romansh | Parodontite | Ausgaben / Dépenses / Spese / Expensas | Bundesamt für Gesundheit (Office fédéral de la santé publique / Ufficio federale della sanità pubblica) (Federal Office of Public Health) | Krankenversicherungsgesetz (KVG/LAMal) |
| Syrian Arab Republic | (Syrian Arab Republic) AND (periodont* OR التهاب دواعم السن) AND (cost OR expenditure OR spending OR revenue OR reimbursement OR النفقات (Al-Nafaqat)) AND (health accounts OR ministry of health OR national OR federal OR insurance OR registry OR register OR database OR data OR dataset OR pubmed OR survey OR interview OR panel OR وزارة الصحة (Ministry of Health) OR Insurance) | Arabic | التهاب دواعم السن | النفقات (Al-Nafaqat) | وزارة الصحة (Ministry of Health) | Insurance |
| Tajikistan | (Tajikistan) AND (periodont* OR Пародонтит) AND (cost OR expenditure OR spending OR revenue OR reimbursement OR Хароҷот (Kharojot)) AND (health accounts OR ministry of health OR national OR federal OR insurance OR registry OR register OR database OR data OR dataset OR pubmed OR survey OR interview OR panel OR Вазорати тандурустӣ ва ҳифзи иҷтимоии аҳолӣ (Ministry of Health and Social Protection of the Population) OR Insurance) | Tajik | Пародонтит | Хароҷот (Kharojot) | Вазорати тандурустӣ ва ҳифзи иҷтимоии аҳолӣ (Ministry of Health and Social Protection of the Population) | Insurance |
| Thailand | (Thailand) AND (periodont* OR โรคปริทันต์) AND (cost OR expenditure OR spending OR revenue OR reimbursement OR ค่าใช้จ่าย (Kha Chai Chay)) AND (health accounts OR ministry of health OR national OR federal OR insurance OR registry OR register OR database OR data OR dataset OR pubmed OR survey OR interview OR panel OR กระทรวงสาธารณสุข (Ministry of Public Health) OR สำนักงานหลักประกันสุขภาพแห่งชาติ (NHSO)) | Thai | โรคปริทันต์ | ค่าใช้จ่าย (Kha Chai Chay) | กระทรวงสาธารณสุข (Ministry of Public Health) | สำนักงานหลักประกันสุขภาพแห่งชาติ (NHSO) |
| Timor-Leste | (Timor-Leste) AND (periodont* OR Periodontite) AND (cost OR expenditure OR spending OR revenue OR reimbursement OR Despesa / Gastu) AND (health accounts OR ministry of health OR national OR federal OR insurance OR registry OR register OR database OR data OR dataset OR pubmed OR survey OR interview OR panel OR Ministério da Saúde OR Insurance) | Portuguese, Tetum | Periodontite | Despesa / Gastu | Ministério da Saúde | Insurance |
| Togo | (Togo) AND (periodont* OR Parodontite) AND (cost OR expenditure OR spending OR revenue OR reimbursement OR Dépenses) AND (health accounts OR ministry of health OR national OR federal OR insurance OR registry OR register OR database OR data OR dataset OR pubmed OR survey OR interview OR panel OR Ministère de la Santé et de l’Hygiène publique (Ministry of Health and Public Hygiene) OR Insurance) | French | Parodontite | Dépenses | Ministère de la Santé et de l’Hygiène publique (Ministry of Health and Public Hygiene) | Insurance |
| Tonga | (Tonga) AND (periodont* OR Periodontitis) AND (cost OR expenditure OR spending OR revenue OR reimbursement OR Expenditure / Fakaʻuli) AND (health accounts OR ministry of health OR national OR federal OR insurance OR registry OR register OR database OR data OR dataset OR pubmed OR survey OR interview OR panel OR Ministry of Health OR Insurance) | English, Tongan | Periodontitis | Expenditure / Fakaʻuli | Ministry of Health | Insurance |
| Trinidad and Tobago | (Trinidad and Tobago) AND (periodont* OR Periodontitis) AND (cost OR expenditure OR spending OR revenue OR reimbursement OR Expenditure) AND (health accounts OR ministry of health OR national OR federal OR insurance OR registry OR register OR database OR data OR dataset OR pubmed OR survey OR interview OR panel OR Ministry of Health OR Insurance) | English | Periodontitis | Expenditure | Ministry of Health | Insurance |
| Tunisia | (Tunisia) AND (periodont* OR التهاب دواعم السن) AND (cost OR expenditure OR spending OR revenue OR reimbursement OR النفقات (Al-Nafaqat)) AND (health accounts OR ministry of health OR national OR federal OR insurance OR registry OR register OR database OR data OR dataset OR pubmed OR survey OR interview OR panel OR وزارة الصحة (Ministry of Health) OR Insurance) | Arabic | التهاب دواعم السن | النفقات (Al-Nafaqat) | وزارة الصحة (Ministry of Health) | Insurance |
| Turkey | (Turkey) AND (periodont* OR Periodontitis) AND (cost OR expenditure OR spending OR revenue OR reimbursement OR Harcama) AND (health accounts OR ministry of health OR national OR federal OR insurance OR registry OR register OR database OR data OR dataset OR pubmed OR survey OR interview OR panel OR Sağlık Bakanlığı OR Sosyal Güvenlik Kurumu (SGK)) | Turkish | Periodontitis | Harcama | Sağlık Bakanlığı | Sosyal Güvenlik Kurumu (SGK) |
| Turkmenistan | (Turkmenistan) AND (periodont* OR Periodontitis) AND (cost OR expenditure OR spending OR revenue OR reimbursement OR Çykdajy) AND (health accounts OR ministry of health OR national OR federal OR insurance OR registry OR register OR database OR data OR dataset OR pubmed OR survey OR interview OR panel OR Saglygy goraýyş we derman senagaty ministrligi (Ministry of Health and Medical Industry) OR Insurance) | Turkmen | Periodontitis | Çykdajy | Saglygy goraýyş we derman senagaty ministrligi (Ministry of Health and Medical Industry) | Insurance |
| Tuvalu | (Tuvalu) AND (periodont* OR Periodontitis) AND (cost OR expenditure OR spending OR revenue OR reimbursement OR Expenditure / Fakapaʻaga) AND (health accounts OR ministry of health OR national OR federal OR insurance OR registry OR register OR database OR data OR dataset OR pubmed OR survey OR interview OR panel OR Department of Health OR Insurance) | English, Tuvaluan | Periodontitis | Expenditure / Fakapaʻaga | Department of Health | Insurance |
| Uganda | (Uganda) AND (periodont* OR Пародонтит) AND (cost OR expenditure OR spending OR revenue OR reimbursement OR Expenditure / Matumizi) AND (health accounts OR ministry of health OR national OR federal OR insurance OR registry OR register OR database OR data OR dataset OR pubmed OR survey OR interview OR panel OR Ministry of Health OR Insurance) | English, Swahili | Пародонтит | Expenditure / Matumizi | Ministry of Health | Insurance |
| Ukraine | (Ukraine) AND (periodont* OR Periodontitis) AND (cost OR expenditure OR spending OR revenue OR reimbursement OR Витрати (Vytraty)) AND (health accounts OR ministry of health OR national OR federal OR insurance OR registry OR register OR database OR data OR dataset OR pubmed OR survey OR interview OR panel OR Міністерство охорони здоров'я України (Ministerstvo okhorony zdorov'ya Ukrayiny) (Ministry of Health of Ukraine) OR Insurance) | Ukrainian | Periodontitis | Витрати (Vytraty) | Міністерство охорони здоров'я України (Ministerstvo okhorony zdorov'ya Ukrayiny) (Ministry of Health of Ukraine) | Insurance |
| United Arab Emirates | (United Arab Emirates) AND (periodont* OR التهاب دواعم السن) AND (cost OR expenditure OR spending OR revenue OR reimbursement OR النفقات (Al-Nafaqat)) AND (health accounts OR ministry of health OR national OR federal OR insurance OR registry OR register OR database OR data OR dataset OR pubmed OR survey OR interview OR panel OR وزارة الصحة ووقاية المجتمع (Ministry of Health and Prevention) OR Insurance) | Arabic | التهاب دواعم السن | النفقات (Al-Nafaqat) | وزارة الصحة ووقاية المجتمع (Ministry of Health and Prevention) | Insurance |
| United Kingdom of Great Britain and Northern Ireland | (United Kingdom of Great Britain and Northern Ireland) AND (periodont* OR Periodontitis) AND (cost OR expenditure OR spending OR revenue OR reimbursement OR Expenditure) AND (health accounts OR ministry of health OR national OR federal OR insurance OR registry OR register OR database OR data OR dataset OR pubmed OR survey OR interview OR panel OR Department of Health and Social Care OR National Health Service (NHS)) | English | Periodontitis | Expenditure | Department of Health and Social Care | National Health Service (NHS) |
| United Republic of Tanzania | (United Republic of Tanzania) AND (periodont* OR Periodontitis) AND (cost OR expenditure OR spending OR revenue OR reimbursement OR Expenditure / Matumizi) AND (health accounts OR ministry of health OR national OR federal OR insurance OR registry OR register OR database OR data OR dataset OR pubmed OR survey OR interview OR panel OR Wizara ya Afya (Ministry of Health) OR National Health Insurance Fund (NHIF)) | English, Swahili | Periodontitis | Expenditure / Matumizi | Wizara ya Afya (Ministry of Health) | National Health Insurance Fund (NHIF) |
| United States of America | (United States of America) AND (periodont* OR Periodontitis) AND (cost OR expenditure OR spending OR revenue OR reimbursement OR Expenditure) AND (health accounts OR ministry of health OR national OR federal OR insurance OR registry OR register OR database OR data OR dataset OR pubmed OR survey OR interview OR panel OR Department of Health and Human Services OR Medicare, Medicaid) | English | Periodontitis | Expenditure | Department of Health and Human Services | Medicare, Medicaid |
| Uruguay | (Uruguay) AND (periodont* OR Periodontitis) AND (cost OR expenditure OR spending OR revenue OR reimbursement OR Gasto) AND (health accounts OR ministry of health OR national OR federal OR insurance OR registry OR register OR database OR data OR dataset OR pubmed OR survey OR interview OR panel OR Ministerio de Salud Pública OR Seguro Nacional de Salud) | Spanish | Periodontitis | Gasto | Ministerio de Salud Pública | Seguro Nacional de Salud |
| Uzbekistan | (Uzbekistan) AND (periodont* OR Пародонтит) AND (cost OR expenditure OR spending OR revenue OR reimbursement OR Xarajatlar) AND (health accounts OR ministry of health OR national OR federal OR insurance OR registry OR register OR database OR data OR dataset OR pubmed OR survey OR interview OR panel OR Sog'liqni Saqlash Vazirligi (Ministry of Health) OR Insurance) | Uzbek | Пародонтит | Xarajatlar | Sog'liqni Saqlash Vazirligi (Ministry of Health) | Insurance |
| Vanuatu | (Vanuatu) AND (periodont* OR Periodontitis) AND (cost OR expenditure OR spending OR revenue OR reimbursement OR Expenditure / Dépenses) AND (health accounts OR ministry of health OR national OR federal OR insurance OR registry OR register OR database OR data OR dataset OR pubmed OR survey OR interview OR panel OR Ministry of Health OR Insurance) | Bislama, English, French | Periodontitis | Expenditure / Dépenses | Ministry of Health | Insurance |
| Venezuela (Bolivarian Republic of) | (Venezuela (Bolivarian Republic of)) AND (periodont* OR Periodontitis) AND (cost OR expenditure OR spending OR revenue OR reimbursement OR Gasto) AND (health accounts OR ministry of health OR national OR federal OR insurance OR registry OR register OR database OR data OR dataset OR pubmed OR survey OR interview OR panel OR Ministerio del Poder Popular para la Salud (Ministry of People's Power for Health) OR Instituto Venezolano de los Seguros Sociales (IVSS)) | Spanish | Periodontitis | Gasto | Ministerio del Poder Popular para la Salud (Ministry of People's Power for Health) | Instituto Venezolano de los Seguros Sociales (IVSS) |
| Viet Nam | (Viet Nam) AND (periodont* OR Viêm nha chu) AND (cost OR expenditure OR spending OR revenue OR reimbursement OR Chi tiêu) AND (health accounts OR ministry of health OR national OR federal OR insurance OR registry OR register OR database OR data OR dataset OR pubmed OR survey OR interview OR panel OR Bộ Y tế OR Bảo hiểm Y tế) | Vietnamese | Viêm nha chu | Chi tiêu | Bộ Y tế | Bảo hiểm Y tế |
| Yemen | (Yemen) AND (periodont* OR التهاب دواعم السن) AND (cost OR expenditure OR spending OR revenue OR reimbursement OR النفقات (Al-Nafaqat)) AND (health accounts OR ministry of health OR national OR federal OR insurance OR registry OR register OR database OR data OR dataset OR pubmed OR survey OR interview OR panel OR وزارة الصحة العامة والسكان (Ministry of Public Health and Population) OR Insurance) | Arabic | التهاب دواعم السن | النفقات (Al-Nafaqat) | وزارة الصحة العامة والسكان (Ministry of Public Health and Population) | Insurance |
| Zambia | (Zambia) AND (periodont* OR Periodontitis) AND (cost OR expenditure OR spending OR revenue OR reimbursement OR Expenditure) AND (health accounts OR ministry of health OR national OR federal OR insurance OR registry OR register OR database OR data OR dataset OR pubmed OR survey OR interview OR panel OR Ministry of Health OR National Health Insurance Scheme) | English | Periodontitis | Expenditure | Ministry of Health | National Health Insurance Scheme |
| Zimbabwe | (Zimbabwe) AND (periodont* OR Periodontitis) AND (cost OR expenditure OR spending OR revenue OR reimbursement OR Expenditure / Mari / Ukusebenzisa) AND (health accounts OR ministry of health OR national OR federal OR insurance OR registry OR register OR database OR data OR dataset OR pubmed OR survey OR interview OR panel OR Ministry of Health and Child Care OR Insurance) | English, Shona, Ndebele | Periodontitis | Expenditure / Mari / Ukusebenzisa | Ministry of Health and Child Care | Insurance |

Supplementary Methods 3: Monte Carlo Simulation Structure

Supplementary Results 1: Search Results for Country Level Expenditure

| Country | Year | Proportion of Dental Expenditure | Income level | Source | Direct/Indirect |
| --- | --- | --- | --- | --- | --- |
| Australia | 2021 | 9.75% | High | Australian Institute of Health and Welfare. (2024). *Oral health and dental care in Australia.* Retrieved from https://www.aihw.gov.au/reports/dental-oral-health/oral-health-and-dental-care-in-australia |  |
| Austria | 2017 | 4.00% | High | Rechnungshof. (2018, April). *Versorgung im Bereich der Zahnmedizin : Bericht des Rechnungshofes (Reihe Bund 2018/24)*. Wien, Österreich: Rechnungshof. [https://www.rechnungshof.gv.at/rh/home/home/Zahnmedizin.pdf](https://www.rechnungshof.gv.at/rh/home/home/Zahnmedizin.pdf?utm_source=chatgpt.com) | Direct |
| Belgium | 2022 | 1.60% | High | Meeus, P., Khalil, A., & Maton, V. (2022). *Health professionals report 2022: Capacity, accessibility and production; specialty of interest: Periodontology.* NNIHDI – Directorate for Research, Development and Quality Promotion. [https://www.healthybelgium.be/images/INAMI/Rapports/HealthcareProviders/HSR2022-Periodontology-Beta.pdf](https://www.healthybelgium.be/images/INAMI/Rapports/HealthcareProviders/HSR2022-Periodontology-Beta.pdf?utm_source=chatgpt.com) | Indirect: based on revenue by practitioner specialty rather than type of treatment |
| Botswana | 2015 | 0.50% | Middle | Menon, V., E. Lang, R. Silva, P. Iyer, and W . Mosime. Estimated Resource Needs for Key Health Interventions Offered Under Botswana’s Essential Health Services Plan 2013–2018. Washington, DC: Futures Group, Health Policy Project. | Direct - NGO estimates |
| Brazil | 2016 | 17.20% | Middle | Trezena S, Oliveira FES, Dias VO, Martelli PJL, Martelli DRB, Martelli Júnior H. Specialized dental care in the Brazilian unified national health system (SUS). Pesqui Bras Odontopediatria Clín Integr. 2023; 23:e220122. | Indirect - based on case mix |
| Cameroon | 2017 | 1.27% | Low | National Technical Group. (2017, December 18). *Establishment of the universal health coverage in Cameroon: Summary of the National Technical Group reflections.* Ministère de la Santé Publique. <https://p4h.world/app/uploads/2023/02/21-Note20SynthC3A8se20des20rC3A9flexions20GTN20CSU20ENGLISH.x23411.docx> | Indirect - based on case mix |
| Canada | 2020 | 0.72% | High | Office of the Parliamentary Budget Officer. (2020, October 7). *Cost estimate of a federal dental care program for uninsured Canadians* (Report No. RP-2021-028-M). Government of Canada. [https://qsarchive-archiveqs.pbo-dpb.ca/web/default/files/Documents/Reports/RP-2021-028-M/RP-2021-028-M_en.pdf](https://qsarchive-archiveqs.pbo-dpb.ca/web/default/files/Documents/Reports/RP-2021-028-M/RP-2021-028-M_en.pdf?utm_source=chatgpt.com) | Direct - based on estimates |
| Chile | 2022 | 4.95% | High | Personal communication on expenditure of public health programs in primary care from Dr Jorge Gamonal Aravena from Universidad de Chile Facultad de Odontologia | Direct |
| Denmark | 2016 | 5.59% | High | Rosing, K., Christensen, L. B., & Damgaard, C. (2021). Periodontal care attendance in Denmark in 2012–2016 – a nationwide register-based study. Acta Odontologica Scandinavica, 80(4), 264–272. https://doi.org/10.1080/00016357.2021.1998611 | Direct |
| Germany | 2023 | 8.50% | High | Kassenzahnärztliche Bundesvereinigung. (2023). *Statistisches Jahrbuch 2023\|2024: Basisdaten zur vertragszahnärztlichen Versorgung; Daten der Jahre 1991–2023* (pp. 40-43, 44). [https://www.kzbv.de/wp-content/uploads/Seite-44-aus-KZBV_JB_2024.pdf](https://www.kzbv.de/wp-content/uploads/Seite-44-aus-KZBV_JB_2024.pdf?utm_source=chatgpt.com) | Direct |
| Ghana | 2022 | 6.20% | Middle | Nimako-Boateng, J., Owusu-Antwi, M., & Nortey, P. (2016). Factors affecting dental diseases presenting at the University of Ghana Hospital. *SpringerPlus*, *5*(1), 1709. https://doi.org/10.1186/s40064-016-3391-y | Indirect - based on case mix |
| Ireland | 2023 | 1.31% | High | Health Service Executive. (2023). *Primary Care Reimbursement Service: Statistical Analysis of Claims and Payments 2023*. Dublin: Health Service Executive. [https://www.hse.ie/eng/staff/pcrs/pcrs-publications/hse-annual-report-2023.pdf](https://www.hse.ie/eng/staff/pcrs/pcrs-publications/hse-annual-report-2023.pdf?utm_source=chatgpt.com) | Indirect: based on case mix |
| Italy | 2017 | 25.80% | High | ISTAT – Istituto Nazionale di Statistica. (2019). *European Health Interview Survey (EHIS) 2019 Italy: Microdata and key results*. Istituto Nazionale di Statistica. [https://www.istat.it/en/non-categorizzato/european-health-interview-survey-ehis/](https://www.istat.it/en/non-categorizzato/european-health-interview-survey-ehis/?utm_source=chatgpt.com) | Indirect: based on proportion of patients receiving specific treatments |
| Japan | 2018 | 13.19% | High | Taira, K., Mori, T., Ishimaru, M., Iwagami, M., Sakata, N., Watanabe, T., Takahashi, H., & Tamiya, N. (2021). Regional Inequality in Dental Care Utilization in Japan: An Ecological Study Using the National Database of Health Insurance Claims. *The Lancet regional health. Western Pacific*, *12*, 100170. <https://doi.org/10.1016/j.lanwpc.2021.100170> | Indirect - based on case mix |
| Mauritius | 2020 | 10.42% | Middle | Ministry of Health & Wellness. (2022, May 24). *National Action Plan for Oral Health 2022‑2027*. Government of Mauritius. [https://health.govmu.org/health/wp-content/uploads/2023/03/National-Action-Plan-for-Oral-Health-2022-2027-1.pdf](https://health.govmu.org/health/wp-content/uploads/2023/03/National-Action-Plan-for-Oral-Health-2022-2027-1.pdf?utm_source=chatgpt.com) | Indirect: case mix |
| Nigeria | 2012 | 17.40% | Middle | Taiwo, Olanrewaju Abdurrazaq; Soyele, Olujide Oladele; Ndubuizu, Godwin Ugochukwu. Pattern of utilization of dental services at Federal Medical Centre, Katsina, Northwest Nigeria. Sahel Medical Journal 17(3):p 108-111, Jul–Sep 2014. \| DOI: 10.4103/1118-8561.140294 | Indirect - based on case mix |
| Norway | 2023 | 27.56% | High | Helsedirektoratet & HELFO. (2024). *Helserefusjonsdata: Landet – fagdistrikt TH – 2024* . Opne‑data. <https://opne-data.helserefusjon.no/?omraade=landet&tidsperiodeType=aar&tidsperiodeFra=2024&tidsperiodeTil=2024&fagomraade=TH> | Direct |
| Republic of China (Taiwan) | 2020 | 9.80% | High | Chen, T., Liu, W., Cheng, S., Lin, S., & Lin, W. (2024). Trends of dental service utilization and expenditure in Taiwan from 2000 to 2020. Journal of Dental Sciences. | Direct |
| Republic of Korea | 2017 | 9.47% | High | Shin H, Cho HA, Kim BR. Dental Expenditure by Household Income in Korea over the Period 2008-2017: A Review of the National Dental Insurance Reform. Int J Env | Direct |
| Rwanda | 2014 | 3.20% | Low | Ministry of Health, Rwanda. (2014). *Rwanda Annual Health Statistics Booklet 2014* (Table 29, p. 78: Non‑communicable diseases). Kigali, Rwanda: Ministry of Health. [https://moh.prod.risa.rw/fileadmin/user_upload/Moh/Publications/Health_Data/Rwanda_Annual_Health_Statistics_Booklet_2014_.pdf](https://moh.prod.risa.rw/fileadmin/user_upload/Moh/Publications/Health_Data/Rwanda_Annual_Health_Statistics_Booklet_2014_.pdf?utm_source=chatgpt.com) | Indirect - based on case mix |
| Saudi Arabia | 2018 | 3.60% | High | Alsanea, R., Tawfiq, W. M., Alswailem, O. A., Alali, T., & AlBarkheel, A. R. (2022). Transformation of dental services from a governmental model to a revenue-generation model of operation in a tertiary care hospital: a health economics assessment. *Annals of Saudi medicine*, *42*(6), 351–358. https://doi.org/10.5144/0256-4947.2022.351 | Direct |
| Seychelles | 2023 | 3.29% | High | Ministry of Health. (2024, July). *Annual Health‑Sector Performance Report 2023* [Report]. Republic of Seychelles. [https://www.health.gov.sc/wp-content/uploads/Annual-Health-Sector-Performance-Report-2023.pdf](https://www.health.gov.sc/wp-content/uploads/Annual-Health-Sector-Performance-Report-2023.pdf?utm_source=chatgpt.com) | Indirect - based on case mix |
| Sweden | 2020-2022 | 7.70% | High | SKaPa – Quality Registry for Caries and Periodontal Diseases. (2023). *Årsrapport 2022* (p. 90). Retrieved from <http://www.skapareg.se/wp-content/uploads/2023/06/SKaPa_2022.pdf> | Direct |
| United Arab Emirates | 2015 | 6.07% | High | Ministry of Health and Prevention. (2022, July 25). *Number of dental treatments categorised by visit, follow up, treatment type and medical district, 2015* [Dataset]. UAE Open Data Portal. https://opendata.fcsc.gov.ae/%40ministry-health-and-prevention/dental-treatments-type-and-medical-district/r/Dental%20Treatments%20By%20Type%20and%20Medical%20District? | Direct |
| United Kingdom of Great Britain and Northern Ireland | 2024 | 3.79% | High | NHS Business Services Authority. (2024, August 22). *Dental statistics – England 2023/24: National overview summary tables (Tables 5a & 5c)*. [https://www.nhsbsa.nhs.uk/statistical-collections/dental-england/dental-statistics-england-202324](https://www.nhsbsa.nhs.uk/statistical-collections/dental-england/dental-statistics-england-202324?utm_source=chatgpt.com) | Indirect - based on case mix |
| United Republic of Tanzania | 2018 | 0.60% | Low | Nyamuryekung'e, K. K., Lahti, S., & Tuominen, R. (2019). Costs of dental care and its financial impacts on patients in a population with low availability of services. *Community dental health*, *36*(2), 131–136. https://doi.org/10.1922/CDH_4389Nyamuryekung'e06 | Indirect - based on case mix |
| United States of America | 2018 | 3.35% | High | Tranby, E. P., Frantsve-Hawley, J., Minter-Jordan, M., Thommes, J., Jacob, M., Monopoli, M., Okunev, I., & Boynes, S. G. (2022). A cross-sectional analysis of oral health care spending over the life span in commercial- and Medicaid-insured populations. Journal of the American Dental Association (1939), 153(2), 101–109.e11. https://doi.org/10.1016/j.adaj.2021.07.028 | Direct - claims data |

Supplementary Results 2: Search Results for Country Level Procedure Charges

| Country | Source(s) | Income Level |
| --- | --- | --- |
| Australia | Department of Veterans’ Affairs. (2023). *Fee schedules for medical services, dental and allied health – Australia* (effective 1 July 2023) [Fee schedule]. [https://www.dva.gov.au/providers/fees-claims/fee-schedules-for-gps-and-specialists](https://www.dva.gov.au/providers/fees-claims/fee-schedules-for-gps-and-specialists?utm_source=chatgpt.com)  Bupa Health Services Pty Ltd. (2023, July 1). *Dental Schedule of Fees – ADF Services: Prices effective from 1 July 2023* [Fee schedule]. [https://www.bupa.com.au/-/media/dotcom/files/adf/2023‑2024/adf‑provider_schedule‑of‑fees_dental_2023‑24.pdf](https://www.bupa.com.au/-/media/dotcom/files/adf/20232024/adfprovider_scheduleoffees_dental_202324.pdf)  WorkSafe Victoria. (2024). *Dental services fee schedule* (effective from 1 July 2024) [Fee schedule]. [https://www.worksafe.vic.gov.au/dental-services-fee-schedule](https://www.worksafe.vic.gov.au/dental-services-fee-schedule?utm_source=chatgpt.com) | High |
| Botswana | Botswana Public Officers’ Medical Aid Scheme. (2019). *Scheme & benefit guide 2019: Dentistry benefit table and tariff* [Benefit guide]. <https://www.bpomas.co.bw/sites/default/files/BPOMAS%20Rulebook_24-07-2019.pdf>  PulaMed. (2022). *Pulamed consultation & procedure tariffs 2022-23* [Tariff schedule]. https://www.pulamed.co.bw/sites/default/files/documents/Pulamed_Consultation_and_Procedure_Tariffs_2022_23.pdf | Middle |
| Brazil | Paraná Dental Board. (2018). *CBHPO: Tabela de honorários de odontologia – Paraná* [Fee schedule]. <https://www.cropr.org.br/uploads/arquivo/b11cb64addec1d3225b169135cb47bb9.pdf> Banco Central do Brasil. (2023). *Tabela de reembolso – Procedimentos odontológicos* [Fee schedule]. Retrieved from <https://www.bcb.gov.br/content/acessoinformacao/bcsaude_docs/Tabela_reembolso_procedimentos_odontologicos.pdf>  Programa de Saúde da Polícia Federal – PF SAÚDE. (2021). *Lista de procedimentos odontológicos* [Fee schedule]. [https://www.gov.br/pf/pt-br/assuntos/pf-saude/documentos/lista-de-procedimento-odontologico-pf-saude-21-11-1-1.pdf](https://www.gov.br/pf/pt-br/assuntos/pf-saude/documentos/lista-de-procedimento-odontologico-pf-saude-21-11-1-1.pdf?utm_source=chatgpt.com) | Middle |
| Canada | Ontario Dental Association. (2021, December). *ODA Suggested Fee Guide for General Practitioners 2022* [Fee schedule]. [https://publish.uwo.ca/~kzhou54/downloads/Fee_Guide-2022_General-Practitioners_FINAL_Online.pdf](https://publish.uwo.ca/~kzhou54/downloads/Fee_Guide-2022_General-Practitioners_FINAL_Online.pdf?utm_source=chatgpt.com)  Indigenous Services Canada. (2021). *NIHB regional dental benefit grids — Quebec* [Fee schedule]. [https://nihb-ssna.express-scripts.ca/en/0205140506092019/04/0407](https://nihb-ssna.express-scripts.ca/en/0205140506092019/04/0407?utm_source=chatgpt.com)  British Columbia Dental Association. (2024). *Suggested Fee Guide for Dental Treatment Services Provided by General Practitioners: 2024* [Fee guide]. [https://www.lincolndentalcenter.ca/wp-content/uploads/2025/03/BCDA-2024-GP-Fee-Guide-2.pdf](https://www.lincolndentalcenter.ca/wp-content/uploads/2025/03/BCDA-2024-GP-Fee-Guide-2.pdf?utm_source=chatgpt.com)  Alberta Dental Association & College. (2020, November). *2021 Guide for Dental Fees for General Dentists* [Fee guide]. [https://www.cdsab.ca/wp-content/uploads/2020/11/2021-ADAC-GP-Guide-for-Dental-Fees.pdf](https://www.cdsab.ca/wp-content/uploads/2020/11/2021-ADAC-GP-Guide-for-Dental-Fees.pdf?utm_source=chatgpt.com) | High |
| Chile | Universidad de Chile. (2023). *Arancel 2023 (reajuste)* [Fee schedule]. Facultad de Odontología. [https://odontologia.uchile.cl/dam/jcr%3A6ed3b3d6-3d52-4934-b115-542406f43113/ARANCEL%202023%20%28REAJUSTE%29.pdf](https://odontologia.uchile.cl/dam/jcr%3A6ed3b3d6-3d52-4934-b115-542406f43113/ARANCEL%202023%20%28REAJUSTE%29.pdf?utm_source=chatgpt.com)  IntegraMédica. (2024). *Arancel dental 2024* [Fee schedule]. [https://www.integramedica.cl/integramedica/site/docs/20240308/20240308153304/arancel_dental_2024.pdf](https://www.integramedica.cl/integramedica/site/docs/20240308/20240308153304/arancel_dental_2024.pdf?utm_source=chatgpt.com)  Colmena. (2024). *Arancel Red Salud 2024* [Fee schedule]. [https://www.colmena.cl/source/wp-content/uploads/2024/07/Colmena-Arancel-RedSalud2024.pdf](https://www.colmena.cl/source/wp-content/uploads/2024/07/Colmena-Arancel-RedSalud2024.pdf?utm_source=chatgpt.com) | Middle |
| China | Hunan Provincial People’s Government. (2020). *关于印发湖南省价⾦标准有关事项的通知* [Notice on the implementation of price standards]. [http://www.hunan.gov.cn/hnszf/xxgk/wjk/szbm/szfzsjg_19847/sylbzj/gfxwj_19835//202111/t20211128_21179544.html](http://www.hunan.gov.cn/hnszf/xxgk/wjk/szbm/szfzsjg_19847/sylbzj/gfxwj_19835/202111/t20211128_21179544.html)  Jiangxi Provincial People’s Government. (2023). *[2023 年地方政府收费标准文件]* [Fee schedule]. <https://www.duyaonet.com/_FileUpLoad/20231204/20231204182416.pdf>  Shandong Provincial People’s Government. (2023). *山东省公立医疗机构医疗服务项目价格（2023年版）* [Price schedule]. <https://ybj.shandong.gov.cn/module/download/downfile.jsp?filename=97e0d0a0b8bd4e6aabdcd424e90f1e30.pdf> | Middle |
| France | Assurance Maladie. (2025, June 11). *Tarifs conventionnels — Chirurgien-dentiste* [Fee schedule]. [https://www.ameli.fr/chirurgien-dentiste/exercice-liberal/facturation-remuneration/tarifs-conventionnels/tarifs](https://www.ameli.fr/chirurgien-dentiste/exercice-liberal/facturation-remuneration/tarifs-conventionnels/tarifs?utm_source=chatgpt.com)  Dentego. (2021). *Tarifs des soins dentaires* [Fee schedule]. Retrieved from [https://web.archive.org/web/20210414070644/https://dentego.fr/nos-tarifs/](https://web.archive.org/web/20210414070644/https:/dentego.fr/nos-tarifs/)  Hospices Civils de Lyon. (2023). *Catalogue des tarifs – Exercice 2023* [Price schedule]. Retrieved from [https://www.chu-lyon.fr/sites/default/files/tarif-2023-19-10-2023.pdf](https://www.chu-lyon.fr/sites/default/files/tarif-2023-19-10-2023.pdf?utm_source=chatgpt.com)  Hôpitaux Universitaires de Strasbourg. (2022, February 17). *Tarifs des prestations des actes dentaires* [Fee schedule]. [https://www.chru-strasbourg.fr/information-legale/tarifs-des-prestations-des-actes-dentaires/](https://www.chru-strasbourg.fr/information-legale/tarifs-des-prestations-des-actes-dentaires/?utm_source=chatgpt.com)  Centre Hospitalier Universitaire de Bordeaux. (2025). *Guide de la tarification – en vigueur 2025* [Fee schedule]. <https://www.chu-bordeaux.fr/CHU-de-Bordeaux/Publications-l%C3%A9gales/Guide-de-la-Tarification/GUIDE-DE-LA-TARIFICATION-EN-VIGUEUR-2025.pdf/> | High |
| Germany | PKV‑Verband. (2025, January 29). *Kommentierung der Gebührenordnung für Zahnärzte (GOZ) – Gebührenteil. Stand: 29. Januar 2025* [Commentary]. Retrieved from [https://www.pkv.de/fileadmin/user_upload/PKV/3_PDFs/GOAE-GOZ/GOZ_Gebuehrenteil.pdf](https://www.pkv.de/fileadmin/user_upload/PKV/3_PDFs/GOAE-GOZ/GOZ_Gebuehrenteil.pdf?utm_source=chatgpt.com)  Bundeszahnärztekammer. (2011, December 5). *Gebührenordnung für Zahnärzte (GOZ) – English version* [Fee schedule]. Retrieved from [https://www.bzaek.de/fileadmin/PDFs/GOZ/gebuehrenordnung_fuer_zahnaerzte_2012_EN.pdf](https://www.bzaek.de/fileadmin/PDFs/GOZ/gebuehrenordnung_fuer_zahnaerzte_2012_EN.pdf?utm_source=chatgpt.com) | High |
| Italy | Regione Emilia‑Romagna. (2024, December 30). *Nomenclatore tariffario regionale delle prestazioni di assistenza specialistica ambulatoriale* [Fee schedule]. [https://salute.regione.emilia-romagna.it/ssr/strumenti-e-informazioni/nomenclatore-tariffario-rer/nomenclatore-30-dicembre-2024-1.pdf](https://salute.regione.emilia-romagna.it/ssr/strumenti-e-informazioni/nomenclatore-tariffario-rer/nomenclatore-30-dicembre-2024-1.pdf?utm_source=chatgpt.com)  Azienda Sanitaria Locale Roma 2. (2024). *Tariffario delle prestazioni di specialistica ambulatoriale* [Fee schedule]. Regione Lazio. <https://www.aslroma2.it/attachements/j4/com_content.article/DG1187-tariffario-specialistica-ambulatoriale.pdf>  Ordine dei Medici della Provincia di Como e Lecco. (n.d.). *Nomenclatore e tariffario medio provinciale delle prestazioni odontoiatriche* [Fee schedule]. Retrieved from [https://www.ordinemedicifc.it/wp-content/uploads/2016/10/Nomenclatore-tariffario-prestazioni-odontoiatriche.pdf](https://www.ordinemedicifc.it/wp-content/uploads/2016/10/Nomenclatore-tariffario-prestazioni-odontoiatriche.pdf?utm_source=chatgpt.com)  Blue Assistance. (2019, October 28). *Nomenclatore tariffario odontoiatrico per Cassa Notariato* [Fee schedule]. [https://www.cassanotariato.it/documenti/polizzarealemutua/20191028tariffario_odontoiatrico_per_cassa_notariato.pdf](https://www.cassanotariato.it/documenti/polizzarealemutua/20191028tariffario_odontoiatrico_per_cassa_notariato.pdf?utm_source=chatgpt.com)  INAIL. (2024, June). *Nomenclatore tariffario TAD/EI* [Price list]. Retrieved from <https://www.inail.it/content/dam/inail-hub-site/documenti/protocolli-e-accordi/2024/06/nomenclatore%20tariffario_tadei.pdf> | High |
| Japan | Ministry of Health, Labour and Welfare. (2024). *歯科診療報酬点数表 [Dental Medical Fee Points Table]* [Fee schedule]. [https://www.mhlw.go.jp/content/12404000/000907835.pdf](https://www.mhlw.go.jp/content/12404000/000907835.pdf?utm_source=chatgpt.com) | High |
| Rwanda | Ministry of Health, Rwanda. (2021, March 3). *Dual clinical practice policy* [Policy document]. <https://www.moh.gov.rw/fileadmin/user_upload/Moh/Publications/Policies/DUAL_CLINICAL_PRACTICE_POLICY_APPROVED03112020.pdf> | Low |
| Republic of Korea | Health Insurance Review & Assessment Service. (2022, January 28). *의·치과 한방 수가파일 (‘22.2.1.시행)_전체판 포함* [Fee schedule]. [https://www.hira.or.kr/bbsDummy.do?brdBltNo=9425&brdScnBltNo=4&pgmid=HIRAA020002000100](https://www.hira.or.kr/bbsDummy.do?brdBltNo=9425&brdScnBltNo=4&pgmid=HIRAA020002000100&utm_source=chatgpt.com) | High |
| Spain | Sanitas. (2024). *Precios tratamientos dentales* [Fee schedule]. Retrieved from [https://www.sanitas.es/media/sden/documento/sd‑premium‑servicios‑y‑tarifas.pdf](https://www.sanitas.es/media/sden/documento/sdpremiumserviciosytarifas.pdf)  Adeslas. (2025). *Actos dentales – Adeslas Dental MAX / Adeslas Dental Total* [Fee schedule]. SegurCaixa Adeslas. [https://www.segurcaixaadeslas.es/sites/default/files/2024‑12/ni‑adeslas‑dental‑max‑25.pdf](https://www.segurcaixaadeslas.es/sites/default/files/202412/niadeslasdentalmax25.pdf)  Universidad Rey Juan Carlos. (2023, July 3). *Tarifas Clínica Universitaria URJC* [Fee schedule]. [https://www.scribd.com/document/694000769/TARIFAS‑CLINICA‑UNIVERSITARIA‑URJC‑3‑de‑julio‑2023](https://www.scribd.com/document/694000769/TARIFASCLINICAUNIVERSITARIAURJC3dejulio2023)  Universidad de Sevilla. (n.d.). *Tarifas – Clínica Odontología* [Fee schedule]. [https://odontologia.us.es/zona-pacientes/tarifas](https://odontologia.us.es/zona-pacientes/tarifas?utm_source=chatgpt.com)  Universidad del País Vasco / Euskal Herriko Unibertsitatea. (n.d.). *Tarifas – Clínica Odontológica* [Fee schedule]. [https://www.ehu.eus/es/web/clinica.odontologica/tarifak](https://www.ehu.eus/es/web/clinica.odontologica/tarifak?utm_source=chatgpt.com) | High |
| Sweden | Folktandvården Stockholm. (n.d.). *Prislista – Vår allmäntandvård* [Price list]. Retrieved from [https://www.folktandvardenstockholm.se/behandlingar-och-priser/priser/prislista/](https://www.folktandvardenstockholm.se/behandlingar-och-priser/priser/prislista/?utm_source=chatgpt.com)  Folktandvården Västerbotten. (2024, January 15). *Tandvårdstaxa 2024–01‑15 – 2025‑01‑14* [Fee schedule]. [https://www.1177.se/globalassets/1177/regional/vasterbotten/media/dokument/taxa‑20240115‑tandvard.pdf](https://www.1177.se/globalassets/1177/regional/vasterbotten/media/dokument/taxa20240115tandvard.pdf)  Karolinska Institutet. (2024). *Behandlingar och priser vid Lärarkliniken* [Price list]. Retrieved from [https://ki.se/universitetstandvarden/patient-pa-universitetstandvarden/din‑tandlakare‑nara‑forskningen/behandlingar‑och‑priser‑vid‑lararkliniken](https://ki.se/universitetstandvarden/patient-pa-universitetstandvarden/dintandlakarenaraforskningen/behandlingarochpriservidlararkliniken) | High |
| Switzerland | Swiss Dental Association (SSO). (2021). *Tarifs dentaires – DENTOTAR®* [Fee schedule]. Retrieved from <https://www.sso.ch/it/media/374/download?attachment> | High |
| Republic of China (Taiwan) | National Health Insurance Administration. (2024). *歯科診療給付點數表 （114 年度）* [Dental benefit fee points table (Year 114)]. <https://www.nhi.gov.tw/en/dl‐8‐1899d571eebf4241b538c5411d610168‐2.pdf>  臺北市政府衛生局. (2020, January 17). *臺北市醫療機構牙科收費標準表* [Dental fees schedule for medical institutions in Taipei City]. <https://www-ws.gov.taipei/Download.ashx?icon=..pdf&n=6Ie65YyX5bi6Yar55mC>  臺南市政府衛生局. (2024, July 4). *臺南市醫療機構牙科收費標準表 – 修訂 110/10/20* [Fee schedule]. Retrieved from [https://health.tainan.gov.tw/warehouse/3D00813D-5518-4E9C-998B-AB3088C461C8/%E8%87%BA%E5%8D%97%E5%B8%82%E9%86%AB%E7%99%82%E6%A9%9F%E6%A7%8B%E7%89%99%E7%A7%91%E6%94%B6%E8%B2%BB%E6%A8%99%E6%BA%96%E8%A1%A8-1101020%E4%BF%AE%E8%A8%82.pdf](https://health.tainan.gov.tw/warehouse/3D00813D-5518-4E9C-998B-AB3088C461C8/%E8%87%BA%E5%8D%97%E5%B8%82%E9%86%AB%E7%99%82%E6%A9%9F%E6%A7%8B%E7%89%99%E7%A7%91%E6%94%B6%E8%B2%BB%E6%A8%99%E6%BA%96%E8%A1%A8-1101020%E4%BF%AE%E8%A8%82.pdf?utm_source=chatgpt.com) | High |
| Tanzania | Ndovu Dental Clinic. (2025). *Price list* [Dental procedure fee schedule]. Retrieved from [https://www.ndovudentalclinic.co.tz/price‑list/](https://www.ndovudentalclinic.co.tz/pricelist/)  PEAN Specialized Dental Clinic. (2025). *Price list* [Dental procedure fee schedule]. Retrieved from https://www.peanhealthlance.co.tz/price‑list/ | Low |
| United Kingdom | NHS Business Services Authority. (2024, April). *NHS England dental charges poster – April 2024* [Poster]. Retrieved from [https://www.nhsbsa.nhs.uk/sites/default/files/2024‑04/NHS%20England%20dental%20charges%20poster%20-%20April%202024.pdf](https://www.nhsbsa.nhs.uk/sites/default/files/202404/NHS%20England%20dental%20charges%20poster%20-%20April%202024.pdf)  Bupa Dental Care. (2024). *Treatment and prices* [Dental procedure fee schedule]. Retrieved from <https://www.bupa.co.uk/dental/dental-care/practices/aylesbury/treatment-prices>  Portman Dental & Implant Clinic. (n.d.). *Dental fees* [Fee schedule]. Retrieved from [https://www.scunthorpedentist.co.uk/plans-fees/dental-fees](https://www.scunthorpedentist.co.uk/plans-fees/dental-fees?utm_source=chatgpt.com)  Smile Dental Practice Bromley. (2025, April 1). *Dental treatment fees – effective from 1 April 2025* [Fee schedule]. Retrieved from [https://www.smiledentalpracticebromley.co.uk/dental-fees/](https://www.smiledentalpracticebromley.co.uk/dental-fees/?utm_source=chatgpt.com)  Al‑Fa Perio Clinic. (2020, October). *Al‑Fa Perio Brochure 2020* [Brochure]. https://www.al‑faperio.co.uk/wp‑content/uploads/2020/10/Al‑FaPerio‑Brochure2020.pdf | High |
| United States of America | American Dental Association. (2022). *2022 Survey of Dental Fees* [Data report]. [https://www.scribd.com/document/774116156/Dental-fees-survey](https://www.scribd.com/document/774116156/Dental-fees-survey?utm_source=chatgpt.com)  North Carolina Division of Health Benefits. (2021, April 19). *General Dentist, Oral Surgeon, Pediatric Dentist, Periodontist & Orthodontist Fee Schedule* [Dental fee schedule]. [https://medicaid.ncdhhs.gov/document‑collection/fee‑schedules](https://medicaid.ncdhhs.gov/documentcollection/feeschedules)  Delta Dental of Kentucky. (2021). *Dentist Handbook 2021* [Handbook]. [https://www.deltadentalky.com/content/dam/member-companies/ky/dentist-handbook/Delta%20Dental%20Dentist%20Handbook%202021.pdf](https://www.deltadentalky.com/content/dam/member-companies/ky/dentist-handbook/Delta%20Dental%20Dentist%20Handbook%202021.pdf?utm_source=chatgpt.com) | High |

Supplementary Results 3: Monte Carlo standard errors for super-region total periodontal expenditure estimates (2021)


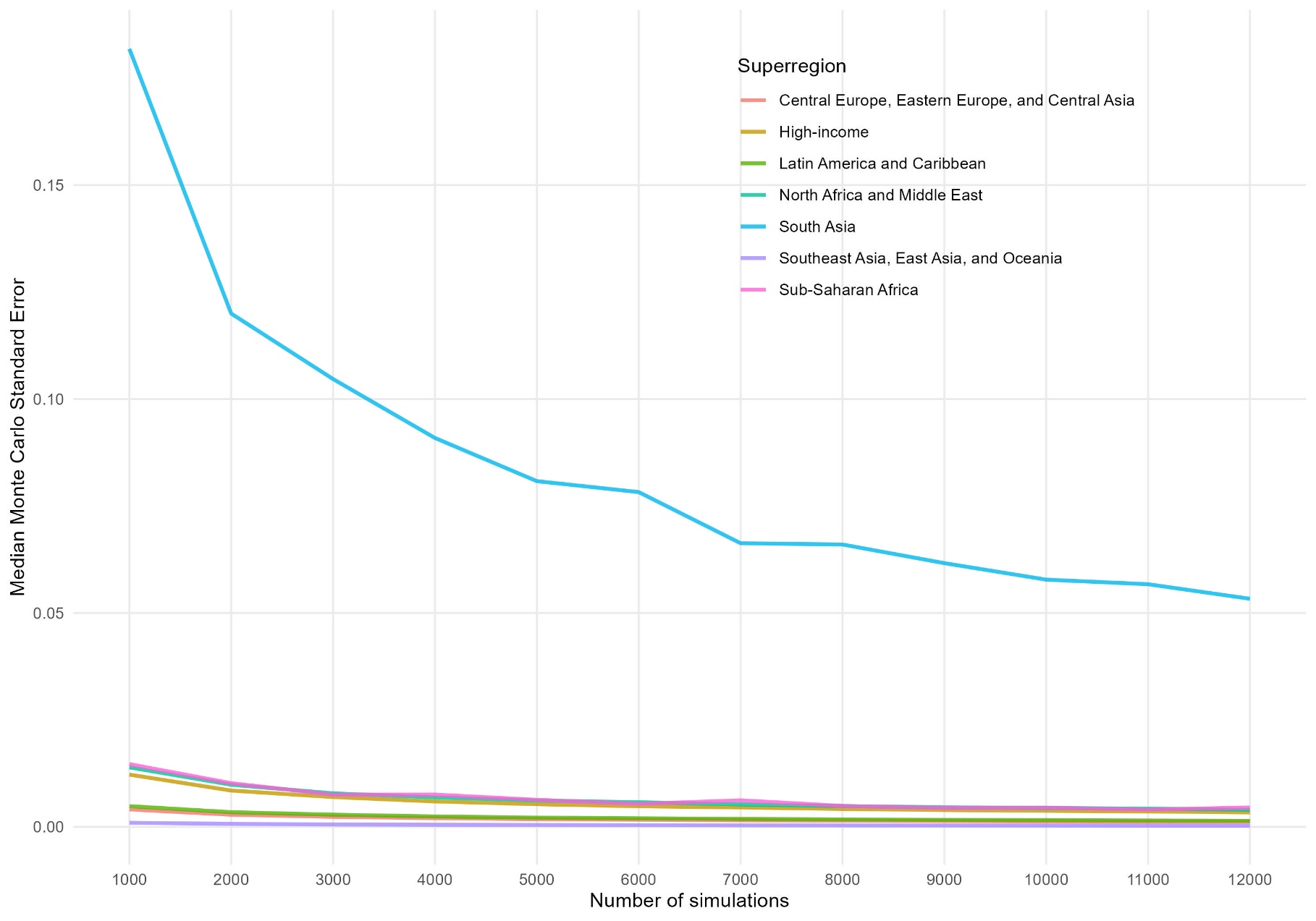


Supplementary Results 4: Sensitivity analysis of global and super-regional total periodontal expenditure in 2021 under alternative expenditure cap assumptions

| Location | 2021 Expenditure (current 75% cap assumption, US$ billion) | Change in Expenditure (60% cap, US$ billion) | Change in Expenditure (60% cap, %) | Change in Expenditure (50% cap, US$ billion) | Change in Expenditure (50% cap, %) |
| --- | --- | --- | --- | --- | --- |
| Global | **168.1** | **-10.32** | **-6.14%** | **-12.86** | **-7.65%** |
| Central Europe, Eastern Europe, and Central Asia | 1.96 | 0 | 0% | -0.12 | -6% |
| High-income | 129.38 | -6.5 | -5.03% | -8.78 | -6.78% |
| Latin America and Caribbean | 3.23 | -2.35 | -72.70% | -2.44 | -75.69% |
| North Africa and Middle East | 3.03 | -1.47 | -48.33% | -1.47 | -48.33% |
| South Asia | 0.87 | 0 | 0% | 0 | 0% |
| Southeast Asia, East Asia, and Oceania | 28.95 | 0 | -0.02% | -0.03 | -0.09% |
| Sub-Saharan Africa | 0.67 | 0 | 0% | -0.03 | -4.28% |

Note: In alternative scenarios, the maximum allowable periodontal expenditure was capped at 60% and 50% of total dental expenditure, compared with the reference assumption of a 75% cap. All other model inputs were held constant. Values are presented in 2025 US$ billions.

Supplementary Results 5: Validation for Monte Carlo Simulation Model Structure based on Known Periodontal Treatment Expenditure


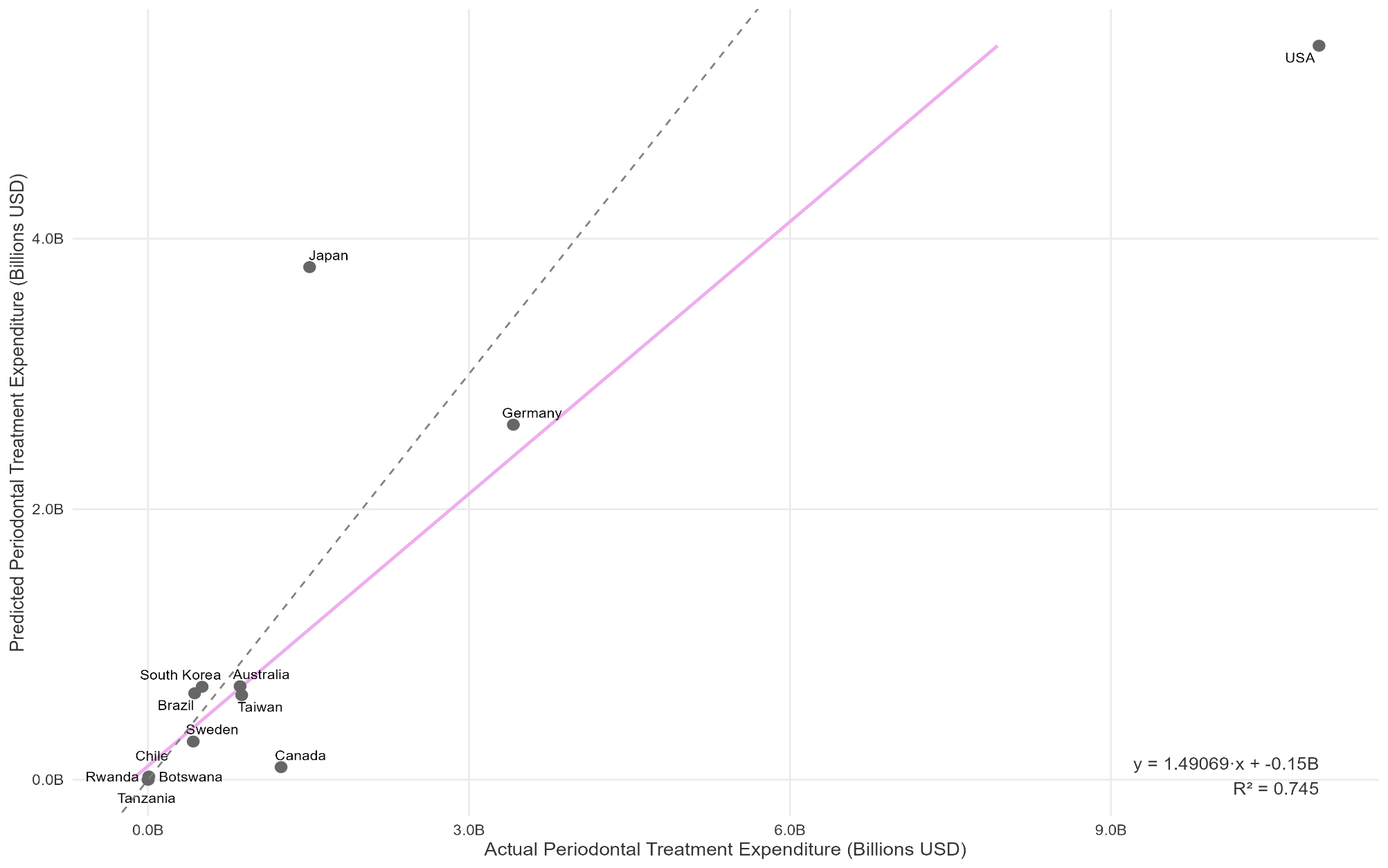


Supplementary Results 6: Sensitivity analysis of 2021 global and super-regional periodontal expenditure under alternative health/gingivitis allocation assumptions

| Location | 2021 Expenditure (current equal-allocation assumption, US$ billion) | Change in Expenditure (60-40 allocation, US$ billion) | Change in Expenditure (60-40 allocation, %) | Change in Expenditure (40-60 allocation, US$ billion) | Change in Expenditure (40-60 allocation, %) |
| --- | --- | --- | --- | --- | --- |
| Global | **168.1** | **-7.56** | **-4.50%** | **7.52** | **4.47%** |
| Central Europe, Eastern Europe, and Central Asia | 1.96 | -0.05 | -2.62% | 0.07 | 3.67% |
| High-income | 129.38 | -6.06 | -4.69% | 5.83 | 4.50% |
| Latin America and Caribbean | 3.23 | -0.11 | -3.35% | 0.04 | 1.12% |
| North Africa and Middle East | 3.03 | -0.08 | -2.57% | 0.09 | 2.95% |
| South Asia | 0.87 | -0.08 | -9% | -0.09 | -9.98% |
| Southeast Asia, East Asia, and Oceania | 28.95 | -1.17 | -4.04% | 1.55 | 5.36% |
| Sub-Saharan Africa | 0.67 | -0.02 | -2.71% | 0.03 | 4.27% |

Note: In the baseline scenario, the dentate population without severe periodontitis was evenly allocated between health/gingivitis and stage I–II periodontitis. Alternative scenarios assigned 60% versus 40%, or 40% versus 60%, to health/gingivitis and stage I–II periodontitis, respectively. All other model inputs were unchanged. Values are presented in 2025 US$ billions.

Supplementary Results 7: Sensitivity analysis of 2021 global and super-regional periodontal expenditure under alternative assumptions on the number of periodontal surgeries by disease stage

| Location | 2021 Expenditure (current assumption, US$ billion) | Change in Expenditure (increased surgery frequency, US$ billion) | Change in Expenditure (increased surgery frequency, %) |
| --- | --- | --- | --- |
| Global | **168.1** | **3.48** | **2.07%** |
| Central Europe, Eastern Europe, and Central Asia | 1.96 | 0.25 | 12.55% |
| High-income | 129.38 | 1.31 | 1.01% |
| Latin America and Caribbean | 3.23 | 0.14 | 4.46% |
| North Africa and Middle East | 3.03 | 0.25 | 8.37% |
| South Asia | 0.87 | 0.76 | 87.39% |
| Southeast Asia, East Asia, and Oceania | 28.95 | 0.55 | 1.91% |
| Sub-Saharan Africa | 0.67 | 0.22 | 32.38% |

Note: In the alternative scenario, the mean number of periodontal surgical procedures per patient was reassigned so that stage III periodontitis and Stage IV case types 1–2 (with higher remaining dentition) received more surgeries than stage IV case types 3–4 (with lower remaining dentition), reversing the baseline assumption. All other model inputs were held constant. Values are presented in 2025 US$ billions.

Supplementary Results 8: Diagnostics for Extrapolation of Prophylaxis Cost


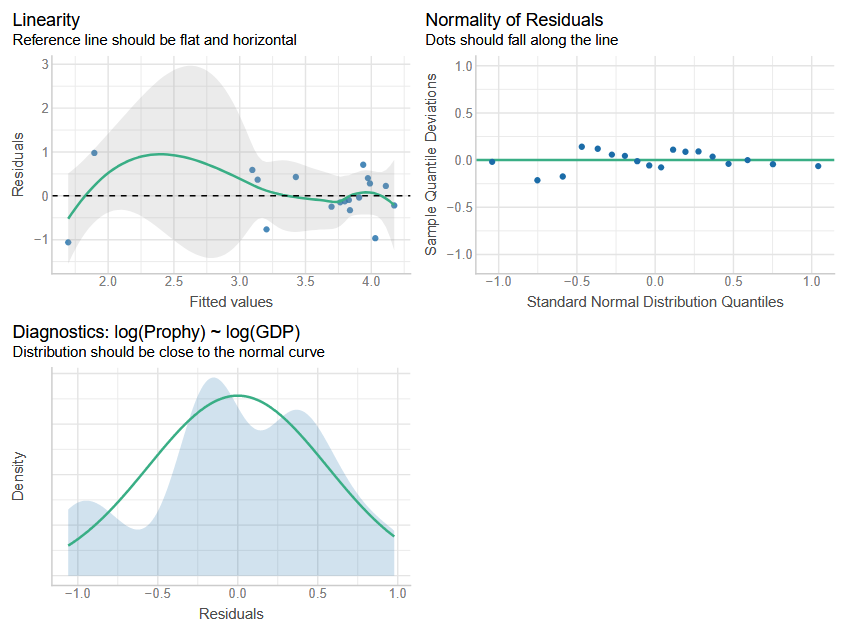


Supplementary Results 9: Diagnostics for Extrapolation of Procedure Costs

| **Consultation (complex)**  **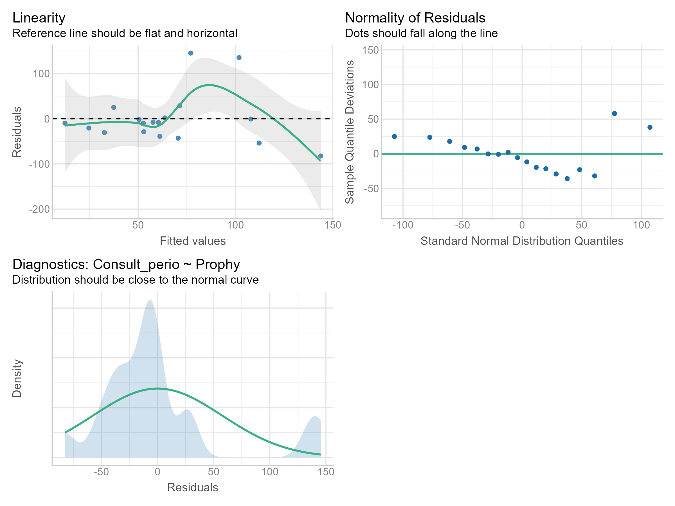** | **Consultation (simple)**  **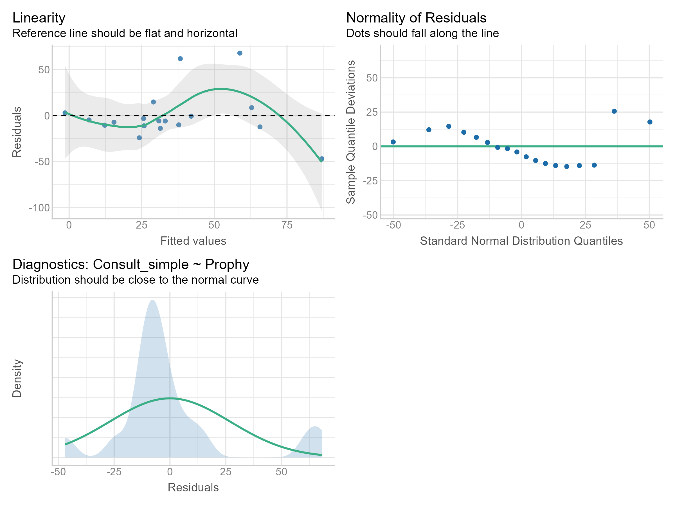** | **Removable prosthesis fabrication**  **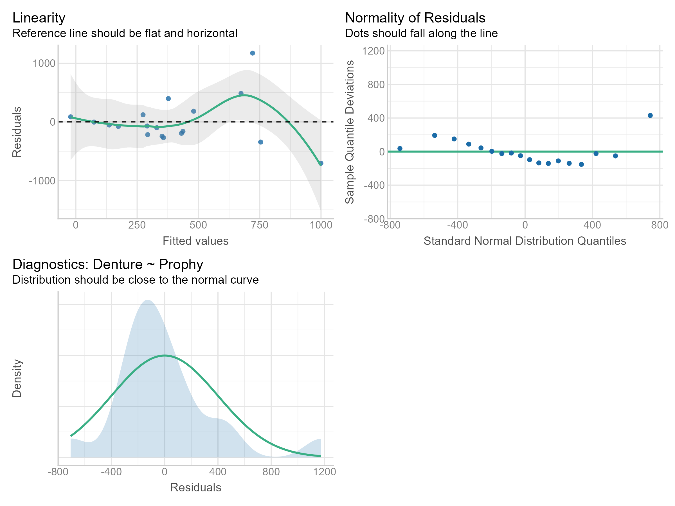** |
| --- | --- | --- |
| **Prosthesis Repair**  **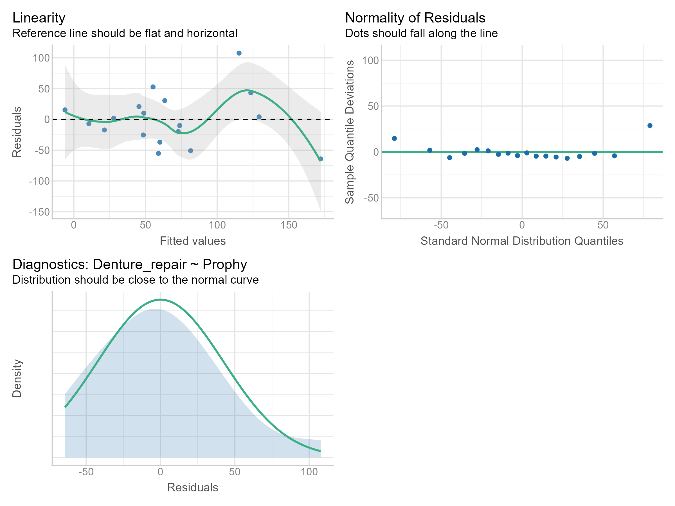** | **Extractions**  **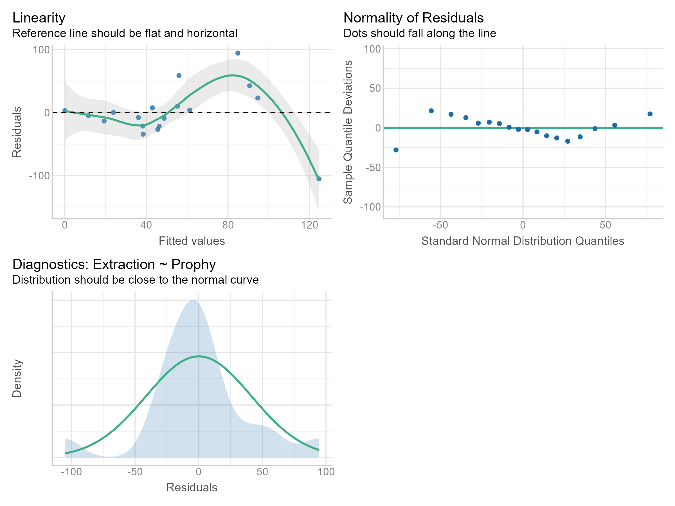** | **Full-arch fixed prosthesis**  **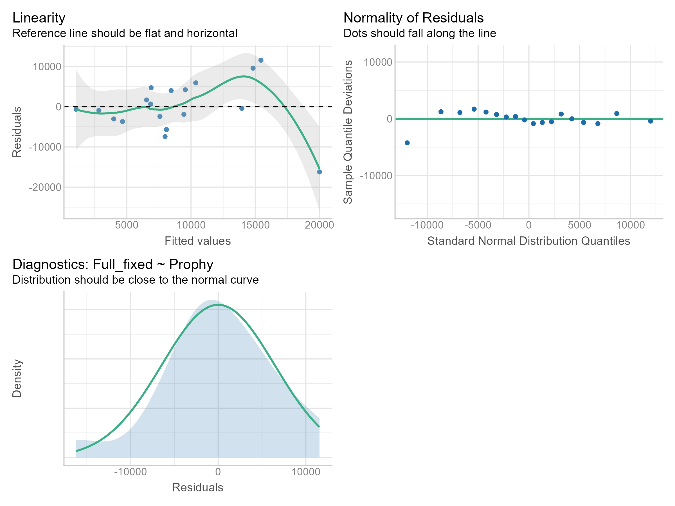** |
| **Periodontal regeneration surgery**  **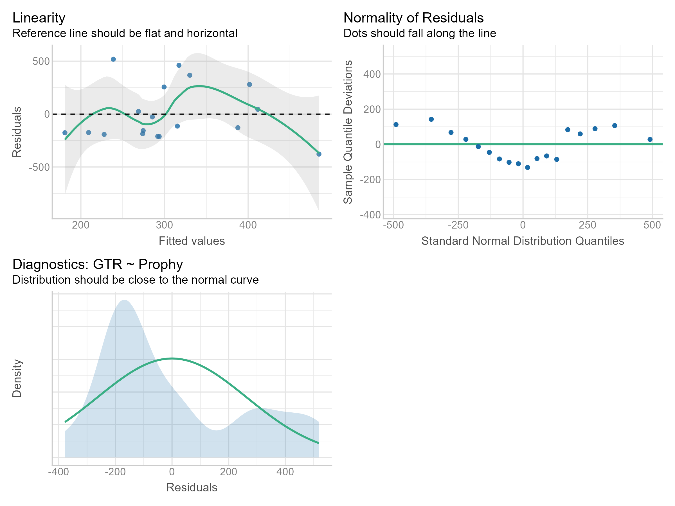** | **Full arch implant surgery**  **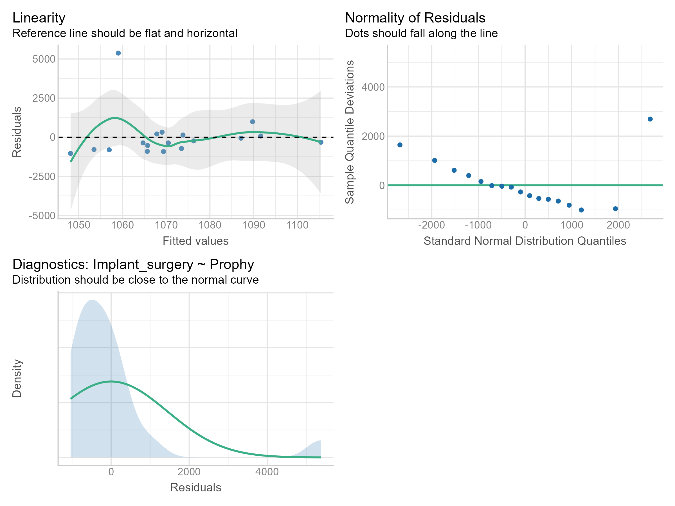** | **Maintenance (complex)**  **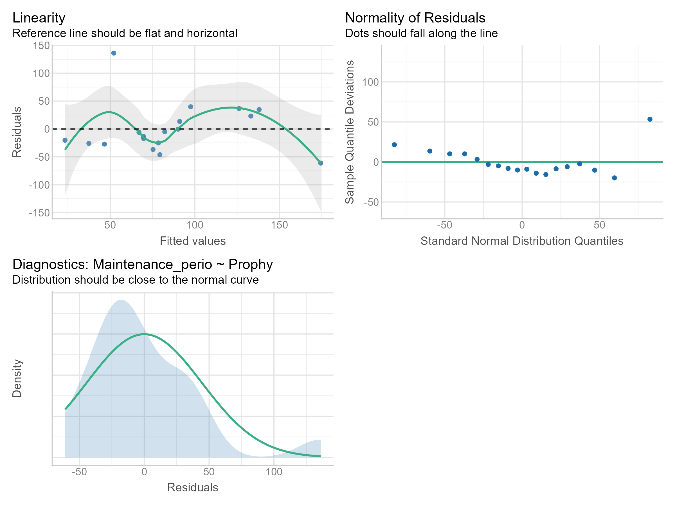** |
| **Maintenance (simple)**  **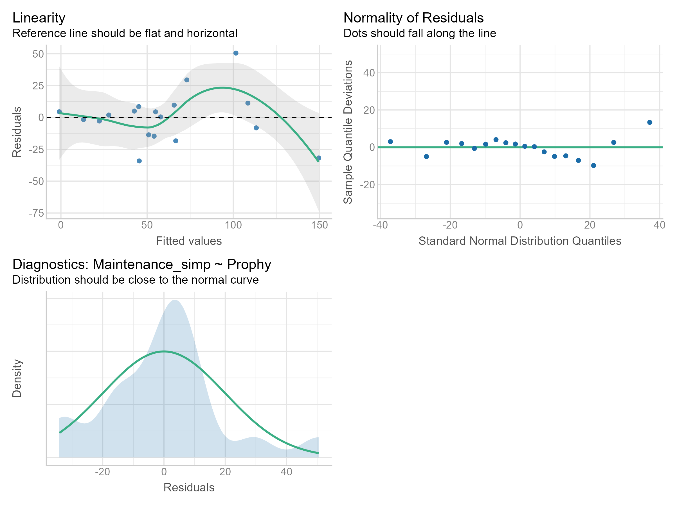** | **Open flap debridement**  **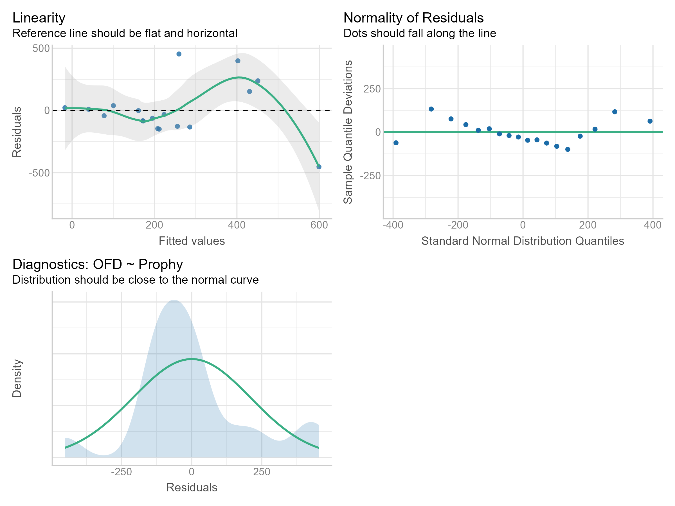** | **Oral hygiene instructions**  **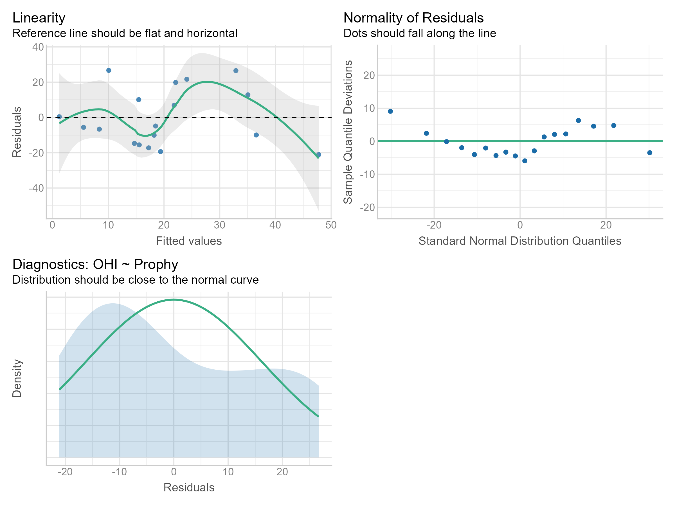** |
| **Panoramic radiograph**  **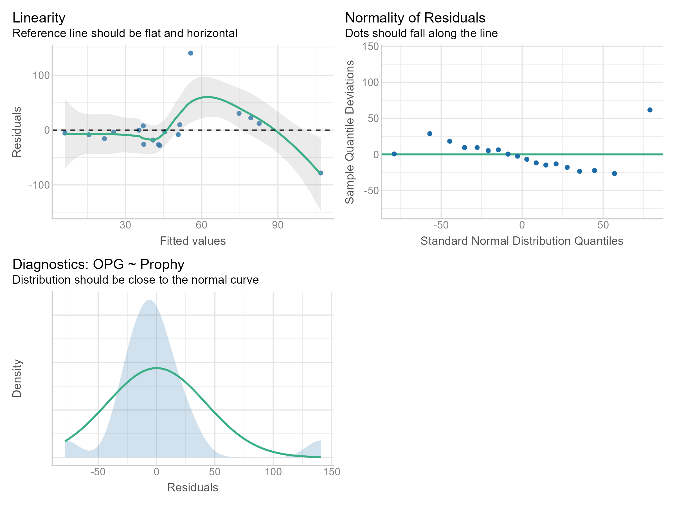** | **Intraoral radiograph**  **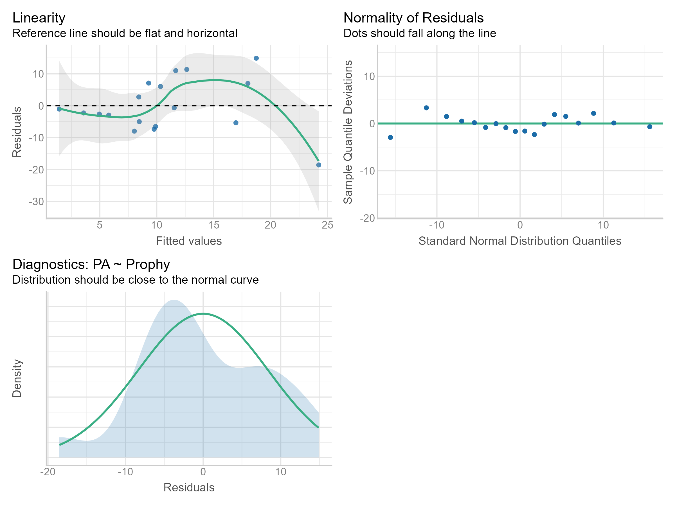** | **Supra- and sub-gingival instrumentation**  **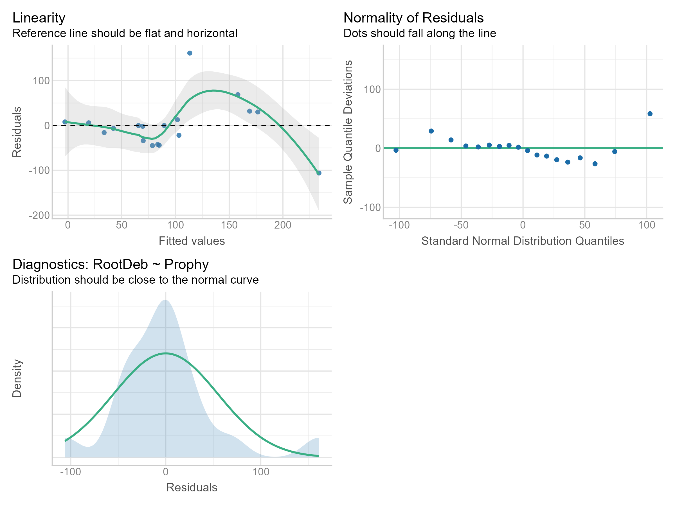** |
| **Single implant and crown**  **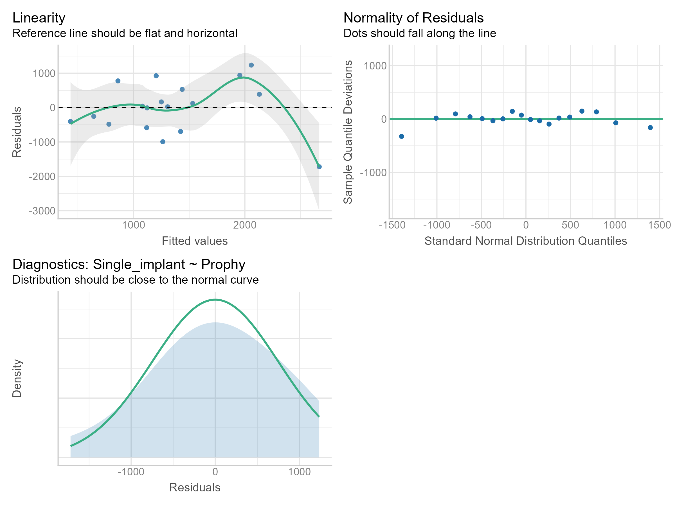** |  |  |

Note: the observed heteroscedasticity and non-normality of residuals reflect the skewed and heavy-tailed nature of cost data and were therefore anticipated. These features do not affect the validity of the simulation outputs, as uncertainty is propagated through repeated stochastic sampling rather than relying on model-based standard errors.

Supplementary Results 10: Validation for Monte Carlo Simulation Model Structure based on Total Dental Expenditure


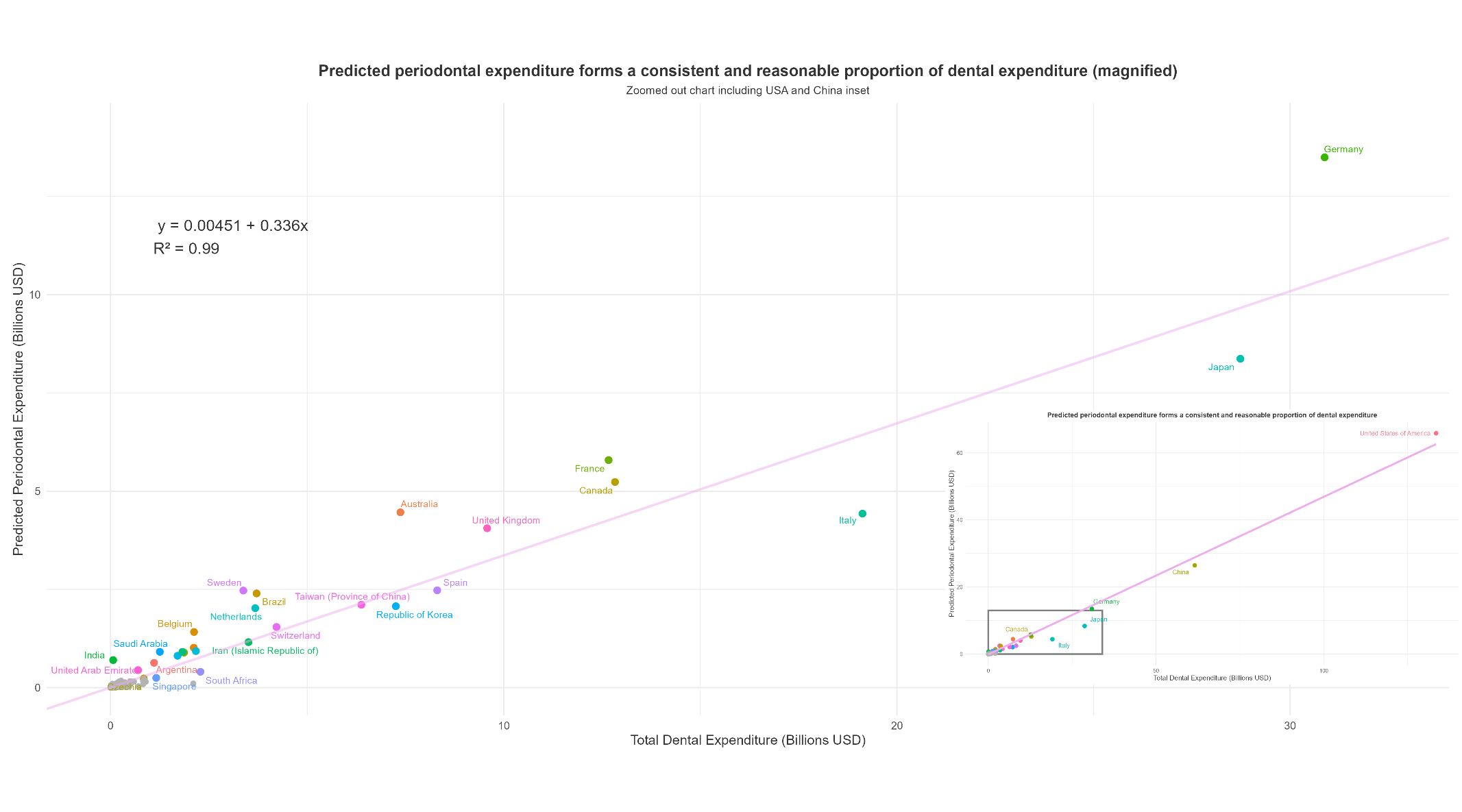


Supplementary Results 11: Country-level total expenditure (A) and per capita expenditure (B) from 2021 to 2050 under the base and WHO target scenarios, highlighting the 5 countries with the highest expenditure in 2050 under the WHO target scenario


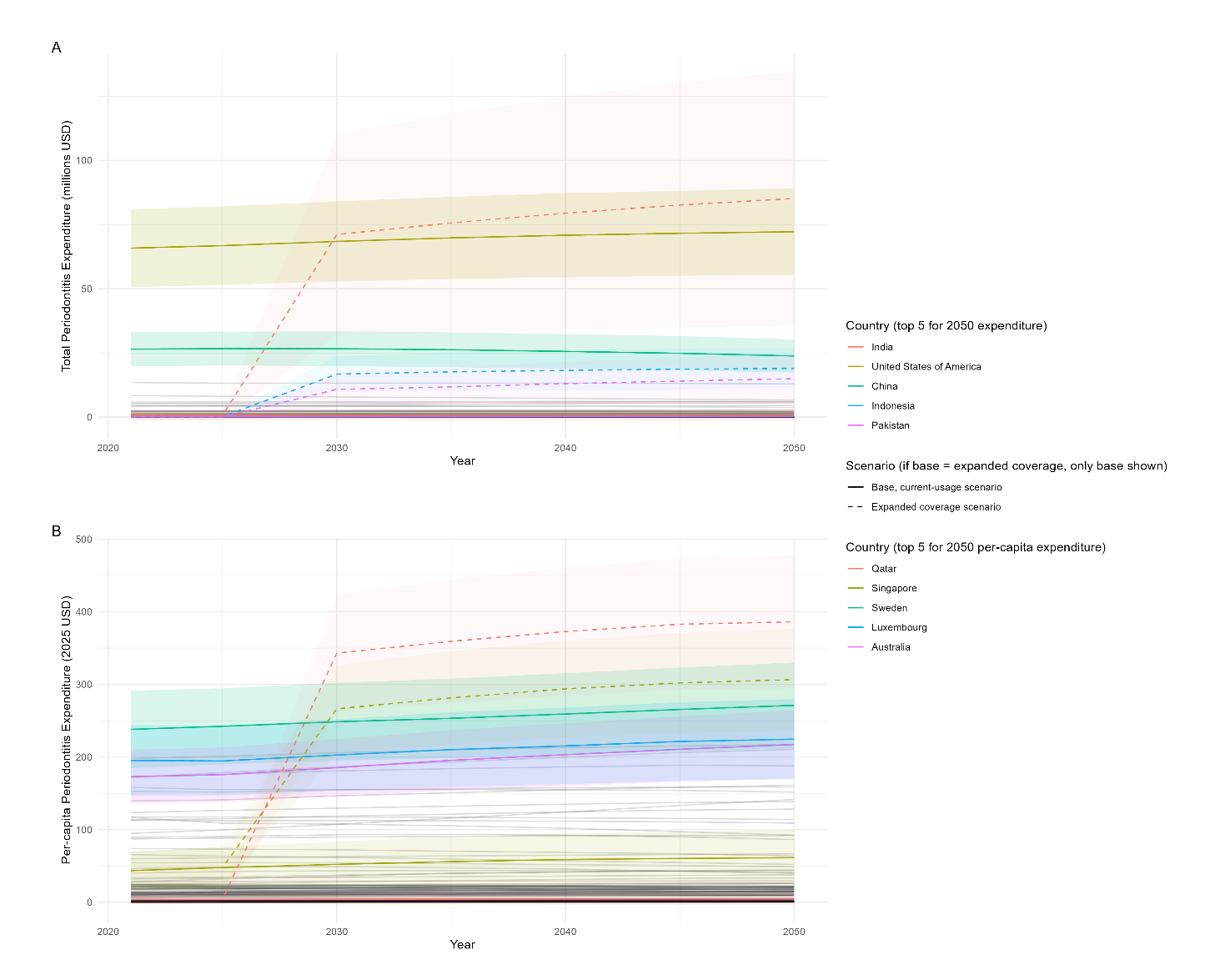

Supplement: Supplementary file 1 — Methods S1: R code for Monte Carlo model (please see Github repository for full code). Methods S2: Detailed search strategy for country‐level expenditure. Methods S3: Search results for country‐level expenditure. Methods S4: Search results for country‐level procedure charges. Methods S5: Monte Carlo standard errors for super‐region total periodontal expenditure estimates (2021). Methods S6: Monte Carlo simulation structure. Methods S7: Validation for Monte Carlo simulation model structure based on known periodontal treatment expenditure. Methods S8: Diagnostics for extrapolation of prophylaxis cost. Methods S9: Diagnostics for extrapolation of procedure costs. Methods S10: Validation for Monte Carlo simulation model structure based on total dental expenditure. Results S1: Country‐level total expenditure (A) and per capita expenditure (B) from 2021 to 2050 under the base and WHO target scenarios, highlighting the five countries with the highest expenditure in 2050 under the WHO target scenario. Results S2: Sensitivity analysis of global and super‐regional total periodontal expenditure in 2021 under alternative expenditure cap assumptions. Results S3: Sensitivity analysis of 2021 global and super‐regional periodontal expenditure under alternative health/gingivitis allocation assumptions. Results S4: Sensitivity analysis of 2021 global and super‐regional periodontal expenditure under alternative assumptions on the number of periodontal surgeries by disease stage. [file JRE-61-518-s001.docx]
